# Adenovirus-Vectored African Swine Fever Virus pp220 Induces Robust Antibody, IFN-γ, and CTL Responses in Pigs

**DOI:** 10.3389/fvets.2022.921481

**Published:** 2022-05-31

**Authors:** Michelle D. Zajac, Neha Sangewar, Shehnaz Lokhandwala, Jocelyne Bray, Huldah Sang, Jayden McCall, Richard P. Bishop, Suryakant D. Waghela, Rakshith Kumar, Tae Kim, Waithaka Mwangi

**Affiliations:** ^1^Department of Diagnostic Medicine/Pathobiology, Kansas State University, Manhattan, KS, United States; ^2^Department of Veterinary Pathobiology, Texas A&M University, College Station, TX, United States; ^3^Department of Veterinary Microbiology and Pathology, Washington State University, Pullman, WA, United States

**Keywords:** African Swine Fever Virus (ASFV), pp220, IgG, IFN-γ, CTL, *SLA-I*, T cell epitopes, subunit vaccine

## Abstract

African Swine Fever Virus (ASFV) poses a serious threat to the pork industry worldwide; however, there is no safe vaccine or treatment available. The development of an efficacious subunit vaccine will require the identification of protective antigens. The ASFV pp220 polyprotein is essential for virus structural integrity. This polyprotein is processed to generate p5, p34, p14, p37, and p150 individual proteins. Immunization of pigs with a cocktail of adenoviruses expressing the proteins induced significant IgG, IFN-γ-secreting cells, and cytotoxic T lymphocyte responses. Four predicted *SLA*-I binding nonamer peptides, namely p34^161−169^, p37^859−867^, p150^1363−1371^, and p150^1463−1471^, recalled strong IFN-γ^+^ PBMC and splenocyte responses. Notably, peptide p34^161−169^ was recognized by PBMCs isolated from 7/10 pigs and by splenocytes isolated from 8/10 pigs. Peptides p37^859−867^ and p150^1363−1371^ stimulated recall IFN-γ^+^ responses in PBMCs and splenocytes isolated from 8/10 pigs, whereas peptide p150^1463−1471^ recalled responses in PBMCs and splenocytes isolated from 7/10 to 9/10 pigs, respectively. The results demonstrate that the pp220 polyprotein contains multiple epitopes that induce robust immune responses in pigs. Importantly, these epitopes are 100% conserved among different ASFV genotypes and were predicted to bind multiple *SLA*-I alleles. The outcomes suggest that pp220 is a promising candidate for inclusion in a prototype subunit vaccine.

## Introduction

High-consequence transboundary animal diseases, such as African Swine Fever [ASF], have an enormous socioeconomic impact on both animal and public health sectors (1–4). The development and deployment of rationally designed treatments and vaccines are crucial in combating and preventing the effects of such diseases (5). Since the introduction of the African Swine Fever Virus [ASFV] into Georgia from Africa, the virus has spread to Europe, Asia, Oceania, and more recently the Dominican Republic and Haiti. In some countries, the disease has become endemic to the extent of endangering food security (6–13). Since there is no vaccine, surveillance by testing with subsequent removal of infected and in-contact animals and enhanced biosecurity measures are the primary control and elimination methods for ASF (5, 14). These methods are moderately effective, but not ideal since they are expensive and labor-intensive. Thus, the development of a safe and efficacious vaccine is high priority (15).

The ASFV is a complex double-stranded DNA virus in the family *Asfarviridae* with the genome encoding more than 150 ORFs. More than 20 ASFV genotypes have been reported so far based on the c-terminal sequence of the gene encoding the p72 capsid protein (16, 17). The virus can infect all members of the Suidae family, but clinical manifestations of a hemorrhagic disease only occur in wild boars and domestic pigs (*Sus scrofa*) of all ages and sexes. The virus has been detected in oral/nasal secretions, blood, feces, and urine, along with raw meat or carcasses of infected pigs (5, 14). Ancestrally, transmission occurs *via* a sylvatic cycle involving the *Ornithodoros* ticks and African wild suids. However, once domestic or feral pigs are infected, transmission occurs primarily by contact and ingestion of contaminated feed, pork products, or soil. Infection with highly virulent ASFV isolates can be lethal to nearly 100% of infected pigs in naïve populations (14, 18–22). Pigs that recover from infection with ASFV of low to moderate virulence and animals vaccinated with attenuated strains or gene-deletion mutants are protected to varying degrees against either homologous or heterologous virulent strains (23–28). The development of a subunit vaccine requires the definition of correlates of protection and identification of cognate antigen(s). Most naïve animals infected with highly virulent ASFV succumb to the disease before the immune system can intervene (29, 30). Macrophages, monocytes, and to some extent DCs support ASFV replication, and the impaired APC functions are potential mechanisms of immune evasion (31–33). Infection occurs *via* the upper respiratory tract where the virus replicates in tonsils and drains to lymph nodes in the head and neck region. Cross-talk between innate and adaptive immune responses is facilitated in the lymph nodes, which makes the regions they drain ideal sites for immunization with ASF vaccines (34–37). The draining lymph nodes are key to the development of mature B cells, cytotoxic T lymphocytes (CTLs), and natural killer (NK) cells, which are involved in the clearance of infected cells (33, 38–41).

Several studies have demonstrated a role for both ASFV-specific antibodies and cellular immunity in protection. However, conflicting data have generated the view that high levels of circulating antibodies do not correlate with protection (42–46). Experiments conducted using porcine PBMCs have demonstrated cross-protection between differing ASFV strains, which is associated with an increase in IFN-γ producing cells (47, 48). In the early stages of infection, clearance of virus-infected cells generally requires CD8^+^ T cell activation (31, 40). Depletion of T cells in pigs immunized with a low-virulence ASFV isolate resulted in the lack of protection following challenge with a virulent strain, which suggests that CD8^+^ T cells are required for protection (49). A connection between IFN-γ secretion and CD8^+^ T-cell activity has been observed in several studies in response to antigenic stimulation or natural infection. Thus, IFN-γ response and CTL activities are logical indicators of immune responses to vaccination (33, 50, 51). However, IFN-γ levels may not reflect protection since the cytokine can be produced by macrophages, CD4^+^, CD8^+^, γδ^+^ T cells, innate B cells, and NK cells on antigen activation (31, 33, 43).

Induction and expansion of CTLs by either high- or low-virulence ASFV strains are still poorly understood. However, CTL responses probably provide the best immune readout for protection induced by ASFV antigens (38, 52–54). Several structural, non-structural, multi-gene-encoded, and uncharacterized ASFV antigens have been evaluated for their potential to elicit protective immunity; however, they did not induce adequate protection to justify development as candidate vaccines, without further research (42, 52, 55–57).

The pp220 (pCP2475L) and pp62 (pCP530R) are two major polyproteins that are cleaved into mature structural proteins for the formation of the core-shell and make up about a third of the ASFV protein mass (58). The pp220 polyprotein is initially processed into a p150 protein and a pp90 preprotein. The latter is cleaved into p34 protein and a precursor pp55 protein, from which p5, p14, and p37 proteins are eventually generated (59, 60). The cytosol of infected cells contains processed forms of pp220 and all the pp220 proteins are also found in the mature virions (60, 61). Both the p14 and p37 proteins have been localized to the cellular nuclei; however, p37 is also found in the cytoplasm, which implies a role in nucleocytoplasmic transport of viral DNA and its protection from DNA sensors of the inflammasomes, which is critical for ASFV replication in the viral factories within the cells (58, 62–68). The abundance of the pp220 antigens in the cytosol means that the antigens are amenable for breakdown by the proteasome, which results in the generation of peptides that could be loaded onto MHC-I molecules for presentation to CD8^+^ T cells. In this study, immunization of pigs with an adenovirus-vectored pp220 polyproteins (ASFV Georgia 2007/1) using two different adjuvants induced antigen-specific antibodies, strong IFN-γ responses, and CTL responses. Lymphocytes from the pigs were used to map T-cell epitopes by screening peptides identified by *in silico* prediction using well-characterized *SLA-I* alleles. Empirical identification and validation of ASFV antigens containing CD8^+^ T cell epitopes, as performed in this study, will be important to inform future subunit vaccine development.

## Materials and Methods

### Plasmid and Virus Construction: p5-34-14-37, p150-I, and p150-II

Three polypeptide sequences from the ASFV pp220 polyprotein (Georgia 2007/1: Genebank Accession FR682468) were designed and used to generate expression constructs. The polypeptide sequences were designated p5-34-14-37, p150-I, and p150-II (p150 was split into two due to its large size). Genes encoding the polypeptides were synthesized (GenScript, NJ, USA) and then cloned into pcDNA3.3-TOPO TA (K8300001, Invitrogen, CA, USA). Following validation of protein expression, the genes were subcloned into pAd/CMV/V5-DEST Gateway (V49320, Invitrogen, CA, USA) for the generation of recombinant adenovirus. The genes were also subcloned into pFastBac HBM TOPO (A11338, Invitrogen, CA, USA) for the generation of baculovirus that was used to produce recombinant proteins needed for immune response readouts. Replication-incompetent adenoviruses encoding ASFV proteins, Ad-p5-34-14-37, Ad-p150-I, and Ad-p150-II were generated using the Invitrogen ViraPower Adenoviral Expression System (K493000, CA, USA). An adenovirus encoding Luciferase (Ad-Luc) was also generated to serve as a negative control. Quality control and validation of protein expression were confirmed by immunocytometric analyses as previously described (54, 57, 69). Viral titers, in infectious focus units per mL (IFU/ml), were determined by immunoassay as previously described (54, 57, 69). To generate recombinant antigens, Bacmids encoding HA-tagged p5-34-14-37, p150-I, or p150-II were transfected into Sf-9 insect cells to produce recombinant baculoviruses and protein expression was confirmed by immunocytometric analyses as previously described (54, 57, 69). A single clone of each recombinant baculovirus was amplified, titrated, and used to infect High-Five cells (B85502, Invitrogen, MO, USA) to express recombinant proteins that were purified by using Anti-HA Agarose affinity purification gel (Sigma, MO, USA).

### Validation of Protein Expression

#### Immunocytometry

Protein expression validation and quality control were assayed by immunocytometric analyses as previously described (54, 57, 69). In brief, duplicate 12-well-plates of Human Embryonic Kidney (HEK) 293A cells were transfected (plasmids), mock transfected (negative controls), or infected (adenoviruses containing each respective construct) with luciferase serving as the negative control for infection. At 48 h post-transfection and 24 h post-infection, the cell monolayers were fixed with cold methanol, rinsed with 1x PBS, blocked for 1 h at room temperature with 1x PBS plus 5% fetal bovine serum (blocking buffer), and then probed with a 1:200 dilution of ASFV-specific convalescent swine serum (E.J. Kramer, Plum Island Animal Disease Center, NY, USA) (69). For the cells probed with the convalescent serum, goat anti-porcine IgG-AP conjugate (Southern Biotech, AL, USA), diluted 1:1000 in blocking buffer, was used as the secondary antibody. Cell staining was visualized with Fast-Red TR/Naphthol AS-MX (Sigma, F4523, MO, USA) AP substrate.

#### Western Blot

HEK 293A cells were transfected as mentioned in Section Immunocytometry and cells were washed at 48 h post-transfection with 1x PBS and then lysed in RIPA buffer supplemented with protease inhibitor (Sigma, MO, USA). Following clarification by centrifugation, supernatants were prepared under reducing conditions in Laemmli buffer (Bio-Rad, CA, USA) containing 10% β-Mercaptoethanol followed by heat denaturation at 95°C for 5 min, fractionated by SDS-PAGE using a 7.5% Acrylamide/bis gel (ProtoGel, National Diagnostics, GA, USA), transferred to PVDF membranes (Amersham, MA, USA), and blocked for 1.5 h at room temperature in SuperBlock (PBS) Blocking Buffer (ThermoFisher, MA, USA). The membranes were incubated with a 1:10,000 dilution of ASFV-specific convalescent swine serum followed by exposure to a 1:8000 dilution of horseradish peroxidase-conjugated swine secondary antibody followed by chemiluminescence development (Pierce Chemiluminescent Plus Substrate Kit, Invitrogen, CA, USA) and detection using the Invitrogen iBright 1500 imaging system. Purified proteins TMSP7 and p62 that were previously validated served as the negative and positive controls, respectively.

### Immunization of Pigs

A cocktail of Ad-pp220 consisting of 10^11^ ifu of Ad-p5-34-14-37, Ad-p150-I, and Ad-p150-II (total 3 x 10^11^ ifu) formulated in an adjuvant was used to immunize pigs intramuscularly as previously described (69). The pigs were boosted with the same dose and *via* the same route 14 weeks post-priming. Control pigs received 3 x 10^11^ ifu of the Ad-Luc virus. Each treatment group contained randomly selected age-matched commercial piglets (*n* = 5) that received either (1) Ad-pp220 cocktail plus ENABL^®^ adjuvant (from BenchMark Biolabs, NE, USA), (2) Ad-pp220 cocktail plus an experimental adjuvant ZTS-01, from Zoetis (NJ, USA), or (3) Ad-Luc plus ENABL^®^ adjuvant (Table 1). The study was terminated after 8 weeks post-boost.

**Table 1:** Swine immunization protocol.

**Groups**	**Swine ID**	**Immunogen (prime-boost dose per pig)**	**Adjuvant**
Ad-pp220-ENABL	34	Ad-pp220 cocktail: Ad-p5-34-14-37 (10^11^ ifu), Ad-p150-I (10^11^ ifu) and Ad-p150-II (10^11^ ifu)	ENABL
	41		
	43		
	46		
	48		
Ad-pp220-ZTS-01	31	Ad-pp220 cocktail: Ad-p5-34-14-37 (10^11^ ifu), Ad-p150-I (10^11^ ifu) and Ad-p150-II (10^11^ ifu)	ZTS-01
	37		
	93		
	94		
	96		
Ad-Luc-ENABL	32	Ad-Luciferase (3 X 10^11^ ifu)	ENABL
	38		
	39		
	44		
	45		

### Sample Collection

During acclimatization of piglets, skin biopsies were collected using 4-mm tissue punches (3785707; American Screening Corp., LA, USA) and processed to generate skin fibroblasts for use as autologous CTL targets. Blood was collected in EDTA-treated or untreated vacutainer tubes once before immunization and then weekly post-prime and post-boost for isolation of peripheral blood mononuclear cells (PBMCs) and serum, respectively. Spleens were collected for isolation of splenocytes beginning on day 8 post-boost (**Figure 2**) as previously described (69).

### Enzyme-Linked Immunosorbent Assay

Antigen-specific IgG responses were evaluated by ELISA using Costar (3590), WA, USA, 96-well-plates coated with 2.5 μg/mL of the affinity-purified antigens in 0.5% bicarbonate buffer as previously described (54, 69). The antigen-coated wells were blocked with 10% nonfat dry milk in PBST (1x PBS + 0.1% Tween 20) before the addition of 1:100 diluted serum samples for the screening assay and two-fold serial dilution for endpoint titer determination. Plates were incubated for 1 h at 37°C, washed 6x using PBST before adding 1:5000 dilution of anti-porcine IgG-POD (peroxidase) antibody (114-035-003, Jackson Immuno-Research, PA, USA) to each well. The plates were further incubated at 37°C for 1 h and then washed 6x with PBST and 3x with PBS. Peroxidase activity was measured by adding Sure Blue tetramethylbenzidine (TMB) substrate (53-00-02, KPL, MA, USA). The reaction was stopped with 1N HCl and the optical density (OD) was measured at 430 nm using a spectrophotometer (BioTek Epoch, VT, USA). End-point titers were calculated by comparing the mean OD of the post-boost serum to that of the baseline at day zero post-immunization (DPI) for each animal. A positive result was determined by selecting the mean value which was higher than the cognate DPI 0 plus 3x the standard deviation (SD).

### Immunohistochemistry

Paraffin-embedded formalin-fixed spleen tissue from a high titer (as determined by qPCR) animal receiving ASFV challenge from a subsequent study was used to ensure that the positive signal represented was authentic. Naïve and ASFV-infected tissues were used for IHC following two 5-min dewaxing in Xylene (Sigma, MO, USA) and rehydration using gradient ethanol (2x 100, 90, 80%) followed by distilled water (all 5 min each). Antigen retrieval was achieved using a 0.1% Protease solution (Sigma, MO, USA) at 37°C for 30 min followed by washing (2x distilled water, 3x 0.1% PBST). Slides were blocked using 5% goat sera (diluted in 0.1% PBST) incubated at room temperature for 40 min. Following a quick wash in 0.1% PBST, a 0.5% solution of Bovine Serum Albumin (BSA) was added for a secondary blocking step at room temperature for 30 min. Primary antibody was tested at 1:200–1:10,000 with the most ideal dilution being 1:2500 for ASFV-specific convalescent sera and 1:250 for swine sera from immunized pigs (0.5% BSA also used as mock for FITC only control). A primary antibody was added following a quick wash (0.1% PBST) and incubated overnight at 4°C. Slides were washed 3x for 7 min each in 0.1% PBST. The secondary antibody was diluted at 1:200 in 0.5% BSA (goat anti-swine IgG-FITC, Jackson ImmunoResearch, PA, USA) and incubated for 1 h at room temperature. Ten-minute washes in 0.1% PBST were repeated a total of 3x following incubation of secondary Ab. DAPI staining and mounting were performed as per the manufacturer's instructions for the VectaTrueVIEW Autofluorescence Quenching Kit w/DAPI (VectorLabs; SP-8500, CA, USA). The use of the Autofluorescence Quenching Kit aided in reducing the potential for autofluorescence and background for clear IFA readouts. Slides were visualized and images were acquired using an Olympus fluorescent microscope paired with CellSens software.

### Peptide Prediction and Selection

NetMHCpan version 2.8 database (http://www.cbs.dtu.dk/services/NetMHCpan-2.8/) was initially used for *in silico* prediction of nonamer peptides from the ASFV pp220 polyprotein that can bind strongly (percent rank <0.5; the default setting for targeting MHC class I binders) to all available Swine Leukocyte Antigen class I (*SLA*-I) alleles within this comprehensive software database to generate a peptide library as previously described (70). After sorting the predicted nonamers based on their predicted scores, a total of 88 putative epitopes were selected and synthesized (Peptide 2.0, Inc, VA, USA). Conservation of the putative epitopes among ASFV genotypes was determined by multi-sequence alignment of the available pp220 polypeptide sequences. The crude nonamer peptides were reconstituted in ultrapure sterile water with 25% DMSO at 10 mg/mL concentration and stored in aliquots at −80°C until use in EliSpot assays.

### IFN-γ EliSpot Assay

The number of IFN-γ-secreting T-cells was determined by the Porcine IFN-γ EliSpot BASIC kit (3130-2A, MabTech, OH, USA) using PBMCs and splenocytes pulsed with peptides as previously described (54, 69). In brief, each sample was assayed in triplicate in MultiScreen-HA 96-well-plates (MAIPS4510, Millipore, MO, USA) with 2.5 x 10^5^ cells/mL cells pulsed with 2.5 μg/mL of each peptide in cRPMI 1640 media. Peptide screening was carried out using four pools [A-D] containing 18 9-mer peptides and a final pool [E] contained the remaining 16 peptides (Table 2). Reactive pools were then tested at the individual peptide level at the same concentration indicated earlier. For each test, positive and negative controls were Phytohemagglutinin (PHA) mitogen at a concentration of 5 μg/mL and media alone, respectively. After a 48-h incubation at 37°C in 5% CO_2_ atmosphere, plates were developed as per the MabTech protocol, the membranes air-dried in the dark, and spots were detected using EliSpot reader (MabTech, OH, USA) and AID software (version 3.4; AutoImmun Diagnostica, Strasburg, Germany). Data are presented as Spot Forming Cells (SFC)/10^6^ PBMCs or splenocytes based upon the mean number of peptide-specific IFN-γ producing cells after subtracting the negative control mean counts as background.

**Table 2:** Predicted *SLA*-I binding peptides from ASFV pp220 (Georgia 2007/1).

**Pool A**		**Pool B**		**Pool C**		**Pool D**		**Pool E**	
Peptide ID	**Sequence**	**Peptide ID**	**Sequence**	**Peptide ID**	**Sequence**	**Peptide ID**	**Sequence**	**Peptide ID**	**Sequence**
1	AINTFMYYY	19	SQWDLVQKF	37	INMRHHTSY	55	YSFEEIACL	73	YVYKTPRWL
2	QIYKTLLEY	20	YGIQNNRSM	38	KSMAAKIFI	56	RRLLNEQNL	74	VSAENIAEF
3	RVFSRLVFY	21	IGMNAVYSL	39	LTTETLFAW	57	LRLRLNLEL	75	FYTHAIQAL
4	SLYPTQFDY	22	SLSNFQALK	40	ETEDVFFTF	58	ASICRQIVL	76	EAMQWFMTM
5	IADAINQEF	23	YTHAIQALR	41	NTLSYWDNI	59	EQYGRVFSR	77	IAASVANKI
6	SAMEVLHEL	24	FIINIRSFK	42	KEIALTPNI	60	RRFYRALEG	78	MAAKIFIVL
7	RLDRKHILM	25	GMNAVYSLR	43	RQMVPMSPL	61	TRLIRNLIF	79	AVNLLRQTF
8	ALDLSLIGF	26	LTHGLRAEY	44	FEHFYTHAI	62	NALMRSIPL	80	KLIQGSESL
9	YTDIVQKKY	27	IYQHFNLEY	45	REFMLKLLI	63	RLLRLRLNL	81	GLISLIDSL
10	TVSAIELEY	28	SYWDNIALR	46	SYEENYATI	64	RYRLYGSDY	82	YYYYVAQIY
11	HIDKNIIQY	29	AGYMSRIFR	47	VMMYNENTF	65	SRLLQIIDF	83	VFNQLIASY
12	LLSKGNAGY	30	LMADTKYFL	48	RTMNDFGMM	66	FYWLEEHLI	84	IYLNLINAF
13	KTLQDVISF	31	MMMVFNQLI	49	IQNNRSMMM	67	YDPLLYPNL	85	NYRANLPLF
14	AGAQLTALF	32	STQAYNDFL	50	TLAQVFESF	68	ITKTFVNNI	86	NYDYSFEEI
15	SLMADTKYF	33	NTFMYYYYV	51	SMMMVFNQL	69	ALIHFVNEI	87	LYDSCSRLL
16	AQEENTLSY	34	TLFAWIVPY	52	NIYNYDYSF	70	LIASYITRF	88	LMPFSLSLY
17	MPFSLSLYY	35	AVMEMGYAH	53	YATILGDAI	71	YINSLTHGL		
18	YTENSVLTY	36	INMRLSMVY	54	YPDPTTEAA	72	YVAQIYSNL		

### CTL Chromium Release Assay

Lytic activity of antigen-specific cells was determined by using the traditional ^51^Cr release assay as previously described (69). To generate effectors, PBMCs collected at 4 weeks post-boost were seeded at a density of 4 x 10^6^ cells/mL per well in a 24-well-plate in 1 ml RPMI 1640 medium (12-167Q, Lonza, IN, USA) containing 45% Click's medium (9195, Irvine Scientific, CA, USA), 10% fetal bovine serum (FBS), 50 mM Mercaptoethanol, 200 mM GlutaMAX (35050061, Gibco, OK, USA), 50 μg/mL Gentamicin, and Penicillin (100 IU/mL)/Streptomycin (100 μg/mL). The PBMCs were stimulated with each adenovirus at an MOI of 1000. Ten days post-stimulation, the cells were harvested, viable cells were purified by Ficoll-Histopaque centrifugation, washed with 1x PBS, and then resuspended in complete RPMI for use as effector cells. For the generation of target cells, skin punch biopsies were minced using a sterile technique to generate primary skin fibroblasts which were cultured in 1 mL of Dulbecco's modified Eagle's medium (DMEM) with 10% FBS, 200 mM GlutaMAX, 50 μg/mL Gentamicin, and Penicillin (100 IU/mL)/Streptomycin (100 μg/mL) per well in 12-well-plates, as previously described (69). Twenty-four hours before the ^51^Cr release assay, autologous skin fibroblasts were transfected with the plasmid construct encoding target antigen using Gene-In transfection reagent (GST-1000, MTI-Global Stem, MD, USA) as per manufacturer's instructions. To prepare the transfected fibroblasts as target cells, the fibroblasts were detached with Accutase, rinsed 3x with DMEM supplemented with 10% FBS, and then labeled with 100 μCi of Na_2_^51^CrO_4_ (Perkin Elmer, MA, USA) per 10^6^ cells for 1 h at 37°C in 5% CO_2_. The labeled fibroblasts were washed 3x and resuspended in a cRPMI 1640 medium. The ^51^Cr release assay was performed in duplicates at effector-to-target (E:T) ratios of 25:1 and 50:1 in a final volume of 100 μL/well using a round-bottom 96-well-plate. Following a 6-h incubation at 37°C in 5% CO_2_, the cells were centrifuged for 4 min at 1,000 rpm and supernatants were collected to measure chromium release. Spontaneous (targets without effectors) and maximum chromium release (lysis with 5% Triton-X detergent solution) were also measured for all target cells. A plasmid construct encoding a Foot and Mouth Disease Virus (FMDV) VP1 and 3D polymerase chimeric antigen was used as a negative control. Chromium release percent-specific lysis values were determined as previously described (71).

### Statistical Analysis

GraphPad Prism, version 6.05, with a significance (*P*-value) of 0.05 was used to analyze all data. A one-way ANOVA followed by Tukey's multiple-comparison test was used to compare the IgG titers of each immunization group. The IFN-γ responses between the treatment group and the negative control group were determined by one-way ANOVA followed by Bonferroni's multiple-comparison test.

### Ethics Statement

Texas A&M University Institutional Animal Care and Use Committee (IACUC) (permit# 2009067) approved Animal Use Protocol 2012-59 that follows the regulations, policies, and guidelines put forth by the Animal Welfare Act, United States Department of Agriculture (USDA) Animal Care Resource Guide, and the Public Health Service (PHS) Policy on Humane Care and Use of Laboratory Animals. All protocols outlined in this document were followed including the use of clinical scoring for daily monitoring and assessment of animal health. Termination was performed using a lethal dose of sodium pentobarbital and confirmation of euthanasia by lack of heartbeat.

## Results

### Design, Expression, and Validation of pp220 Constructs

**Figure 1:**
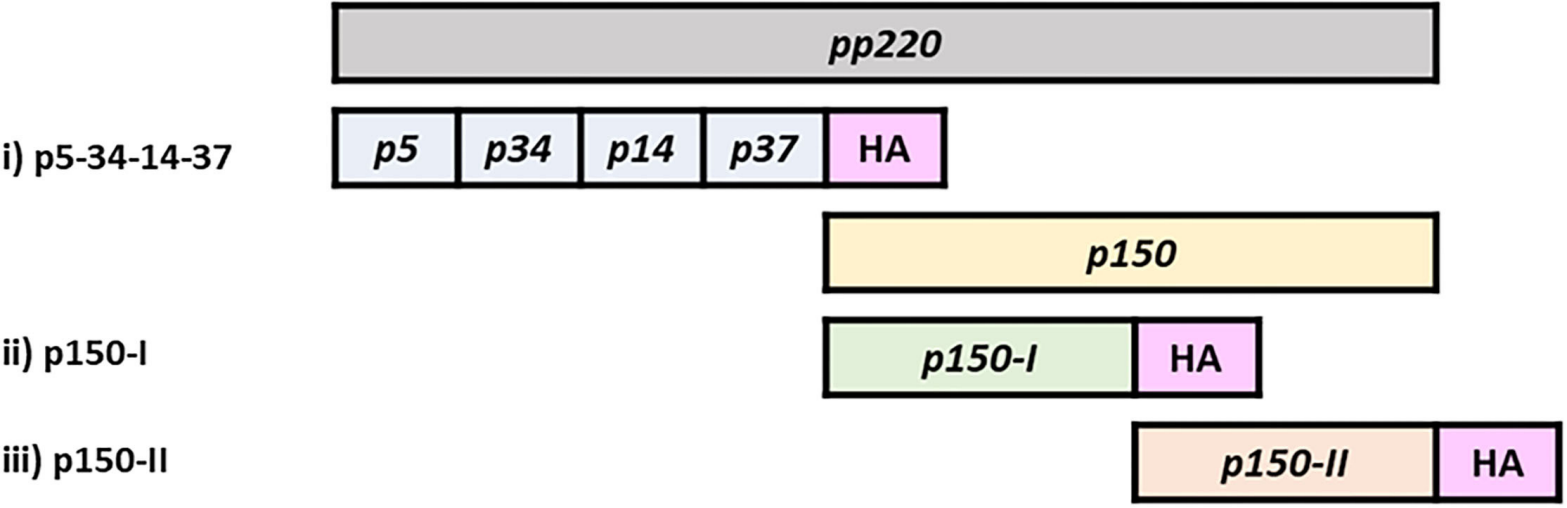
ASFV pp220 expression constructs. Illustration of synthetic genes encoding ASFV pp220 polyprotein. p5-34-14-37 constitute genes encoding structural proteins p5, p34, p14, and p37. Due to its large size, the sequence encoding p150 was split into two genes: p150-I; and p150-II. Synthetic genes had an HA tag added in-frame at the 3' end for tracking protein expression.

**Figure 2:**
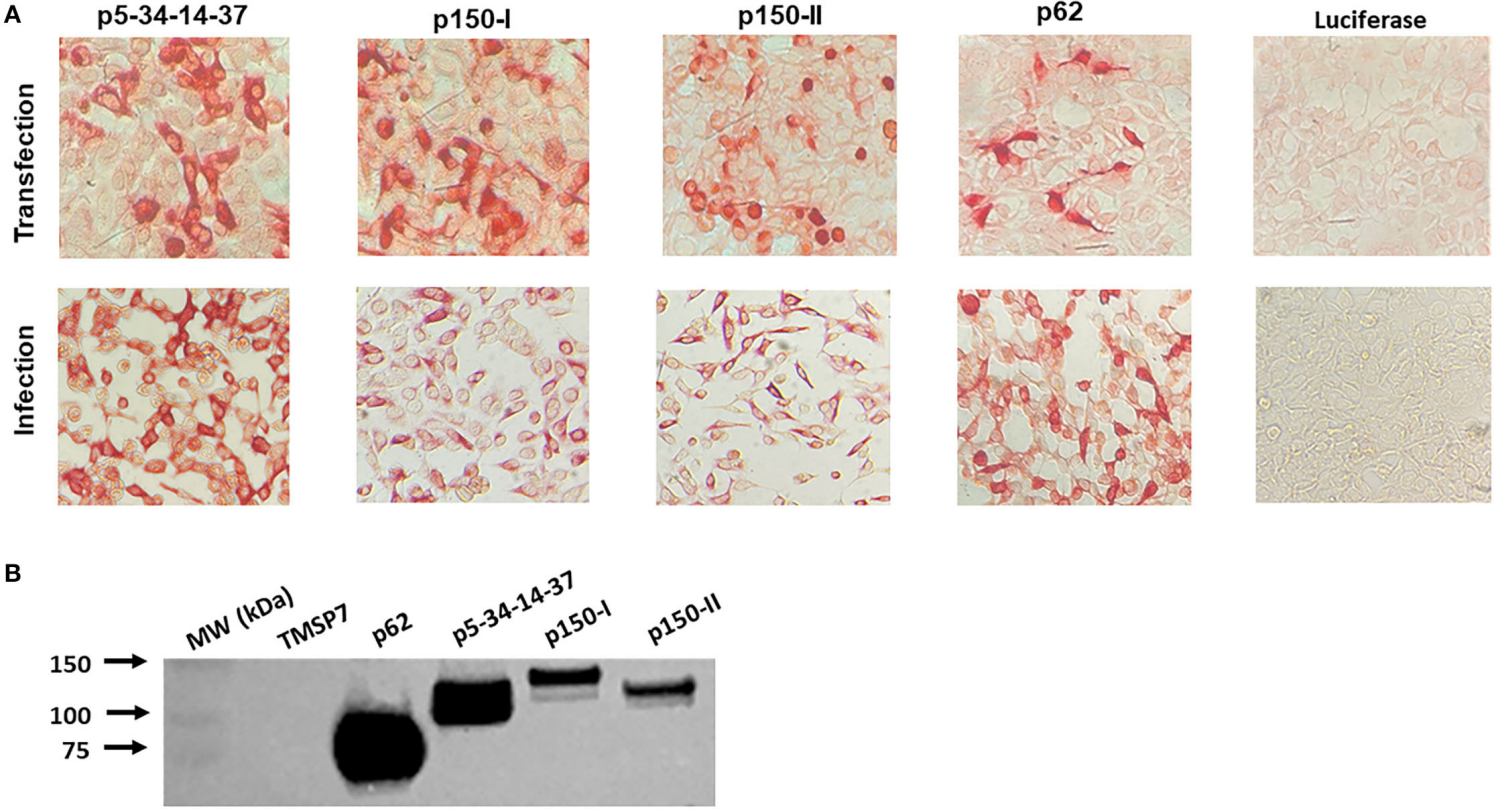
ASFV pp220 construct antigen expression. **(A)** Antigen expression was evaluated by immunostaining of HEK 293A cells transfected with pcDNA or infected with adenovirus encoding each pp220 construct, and **(B**) Western Blot, using proteins produced by transfected HEK 293A cells, probed with ASFV-specific convalescent porcine serum. Recombinant ASFV p62 antigen served as a positive control, whereas an irrelevant recombinant antigen, TMSP7, served as a negative control.

Three recombinant plasmid and adenovirus constructs encoding the components of the pp220 polyprotein from the ASFV Georgia 2007/1 isolate, designated p5-p34-p14-p37, p150-I, and p150-II (each combined encode for the entire pp220 antigen) (Figure 1), were validated for protein expression in transfected and adenovirus-infected HEK 293A cells using ASFV-specific convalescent swine serum (Figure 2A). The authenticity of the antigens was validated by Western Blot using the ASFV-specific convalescent serum. Previously validated purified ASFV p62 antigen served as a positive control, whereas an irrelevant antigen, TMSP7, was used as a negative control (Figure 2B).

### Ad-pp220 Cocktail Primed Strong IgG Responses

**Figure 3:**
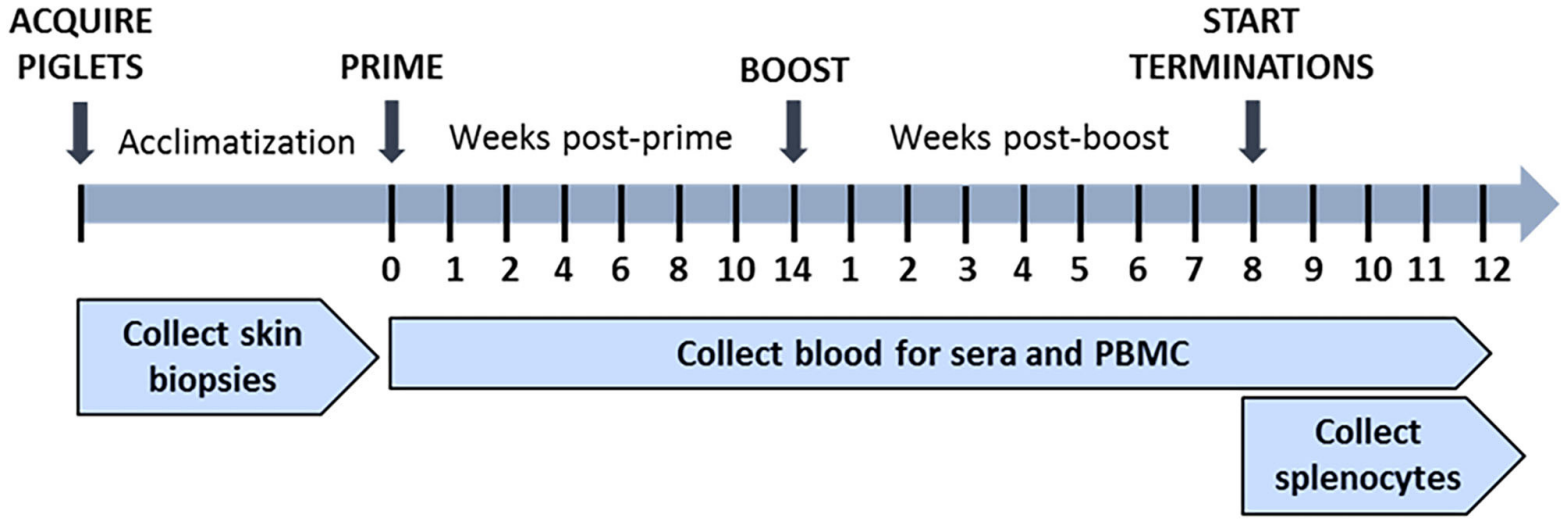
*In vivo* study timeline. Piglets were acclimatized, and skin biopsies were collected prior to immunization. Piglets in treatment groups were primed at week 0 and then boosted at week 14 post-prime with the Ad-pp220 cocktail as shown in Table 1. Negative control piglets were similarly primed and boosted, but with Ad-Luciferase. Pigs from all the groups were terminated after week 8 post-boost. Blood samples were collected weekly post-prime and post-boost for PBMCs and sera. During termination, blood samples were collected, and spleens were harvested.

**Figure 4:**
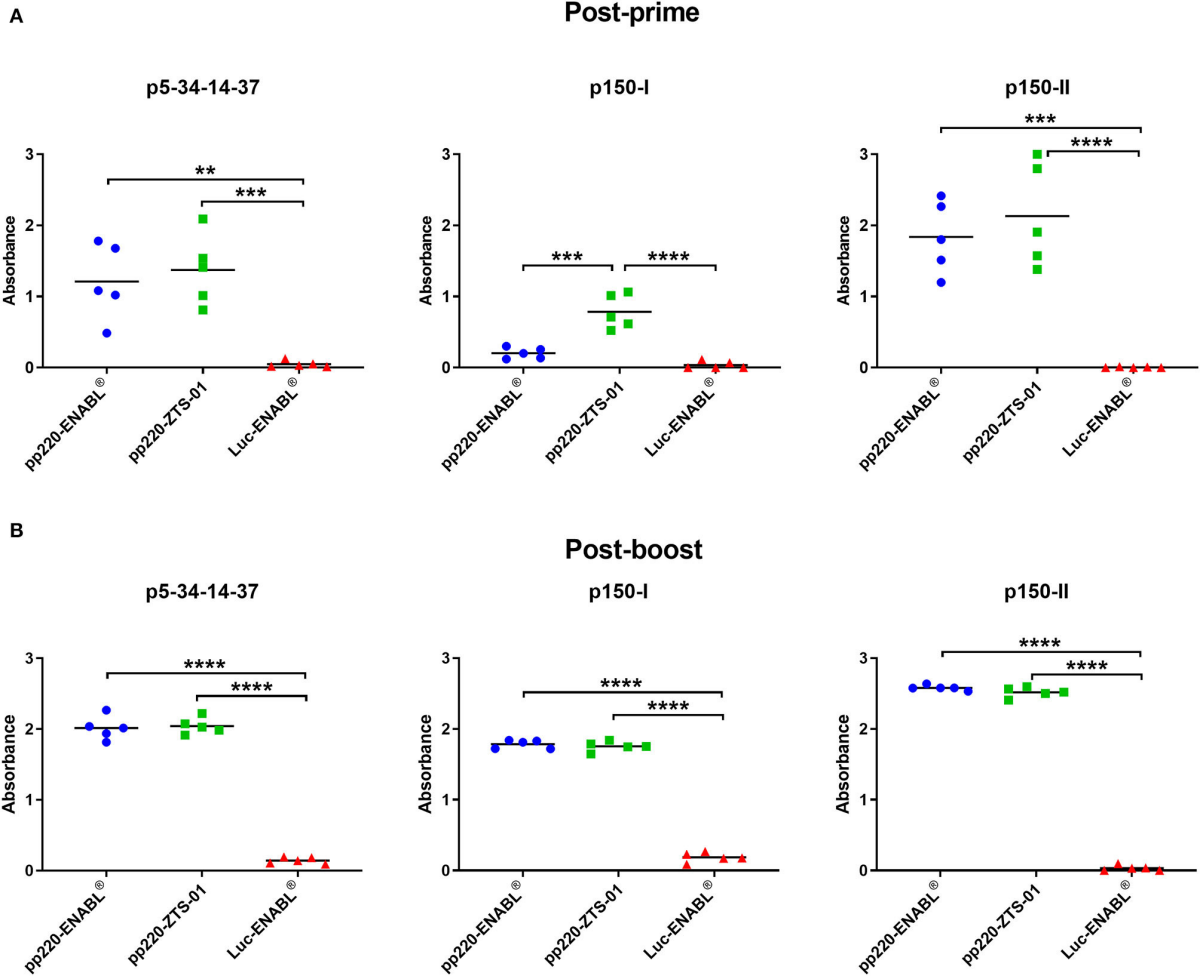
Antibody responses against pp220 antigens. IgG responses against p5-34-14-37, p150-I and p150-II in **(A)** week 4 post-prime sera; and **(B)** week 1 post-boost sera were evaluated by ELISA. Mean responses for the groups are denoted by bars and statistically significant differences between groups is denoted by asterisks (**p < 0.01, ***p < 0.001 and ****p < 0.0001).

Following prime-boost immunization (Table 1), pp220-specific immune responses were evaluated in pigs at defined time points (Figure 3). All pigs immunized with the Ad-pp220 cocktail seroconverted and had detectable post-prime pp220-specific IgG responses (Figure 4A). The highest mean IgG responses against all the three pp220 antigens were observed in pigs immunized with the Ad-pp220 cocktail formulated in ZTS-01 adjuvant (Figure 4A). Both treatment groups, pp220-ENABL^®^ (*p* < 0.01) and pp220-ZTS-01 (*p* < 0.001), had significantly higher p5-p34-p14-p37-specific IgG responses than the negative control group, Ad-Luc-ENABL^®^ (Figure 4A). The Ad-pp220 cocktail formulated in ENABL^®^ adjuvant elicited low levels of post-prime IgG responses against p150-I in pigs (Figure 4A). However, the Ad-pp220-ZTS-01 treatment group had significantly higher mean IgG responses against p150-I compared to the Ad-pp220-ENABL^®^ (*p* < 0.001) treatment group and the Ad-Luc-ENABL^®^ control group (*p* < 0.0001) (Figure 4A). Similar to the p5-p34-p14-p37-specific responses, significantly higher p150-II-specific mean IgG responses were primed in the Ad-pp220-ENABL^®^ (*p* < 0.001) and the Ad-pp220-ZTS-01 (*p* < 0.0001) treatment groups compared to those in the Ad-Luc-ENABL^®^ negative control group (Figure 4A). Following boosting, significantly higher (*p* < 0.0001) IgG responses were recalled against all the three pp220 antigens in pigs from both the treatment groups compared to the negative controls (Figure 4B).

**Figure 5:**
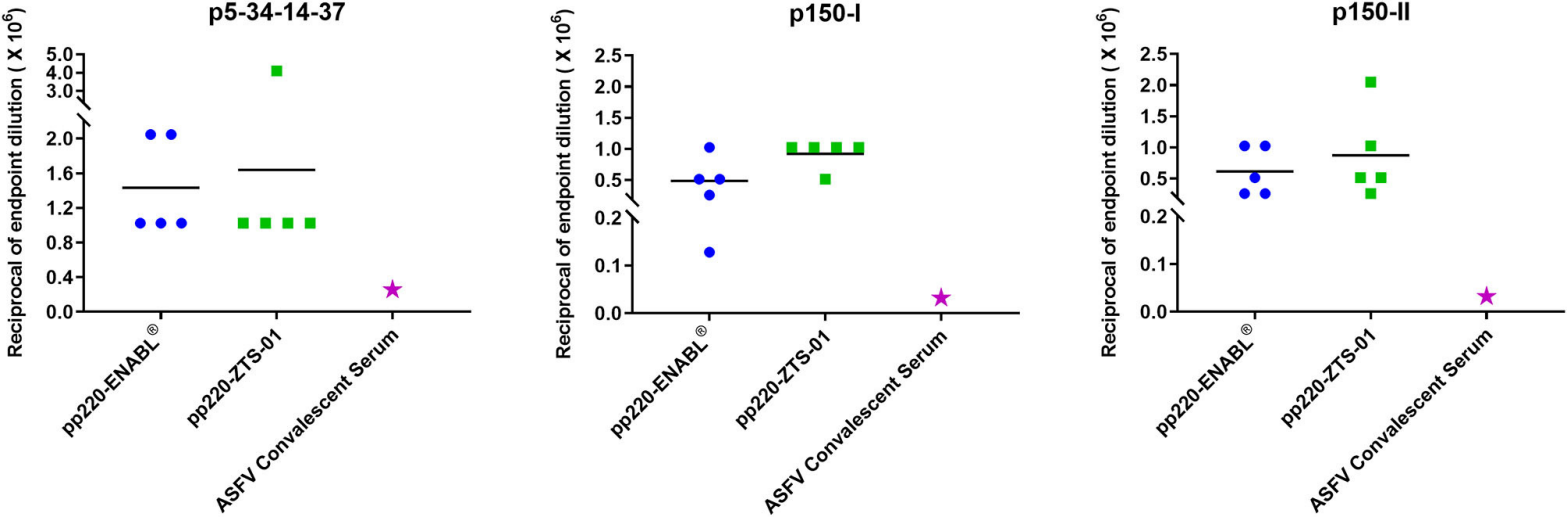
Antibody titers for pp220 antigens. IgG end-point titers were determined by ELISA for p5-34-14-37, p150-I, and p150-II in sera from week 1 post-boost and in ASFV-specific convalescent porcine serum. Mean IgG titers for the groups are denoted by bars, whereas IgG titers in the convalescent serum are denoted by the pink star.

Post-boost, pp220-specific IgG end-point titers elicited in the pigs primed with the Ad-pp220-ENABL^®^ and the Ad-pp220-ZTS-01 formulations were higher than the IgG titers detected in the ASFV-specific convalescent porcine serum (Figure 5). All pigs in both treatment groups developed high levels of IgG titers, in the range of 0.1 x 10^6^ to 4.0 x 10^6^, against p5-p34-p14-p37, p150-I, and p150-II antigens (Figure 5). In comparison, IgG titers detected in the ASFV-specific convalescent serum for p5-p34-p14-p37, p150-I, and p150-II antigens were 1: 2.5 x 10^5^, 1: 3.2 x 10^4^, and 1: 3.2 x 10^4^, respectively (Figure 5).

### Antibodies Induced by the Ad-pp220 Cocktail Recognize Wildtype ASFV

**Figure 6:**
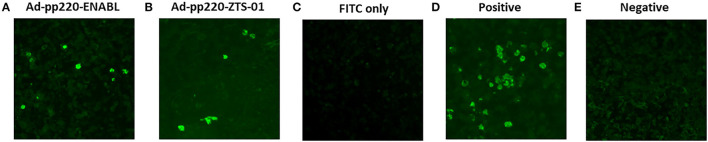
Validation of Induced anti-pp220 antibodies: Authenticity of the antibodies elicited by the adenovirus-vectored pp220 antigens was confirmed by Immunohistochemistry. Formalin-fixed ASFV (Georgia 2007/1) infected swine spleen tissues were probed with sera that was obtained from each group two weeks post-boost: **(A)** Ad-pp220-ENABL; **(B)** Ad-pp220-ZTS-01; **(C)** Secondary (FITC) antibody control: secondary antibody is probed in the absence of primary sera, **(D)** Positive control serum: ASFV specific convalescent swine serum, and **(E)** Negative control serum: Ad-Luc-ENABL.

Sera from the pigs immunized with the adenovirus-vectored pp220 antigens (Ad-pp220-ENABL and Ad-pp220-ZTS-01) recognized cells infected with wildtype ASFV (Georgia 2007/1) following immunohistochemical analysis of ASFV-infected spleen tissue slides using sera obtained 2 weeks post-boost (Figure 6). The ASFV-specific convalescent serum served as a positive control, whereas negative control sera from the mock-immunized pigs as well as secondary FITC controls did not result in antigen detection. The IHC outcome confirmed that immunization with the adenovirus-vectored pp220 antigens elicited ASFV-specific antibody responses (Figure 6).

### Ad-pp220 Cocktail Induced IFN-γ Responses

**Figure 7:**
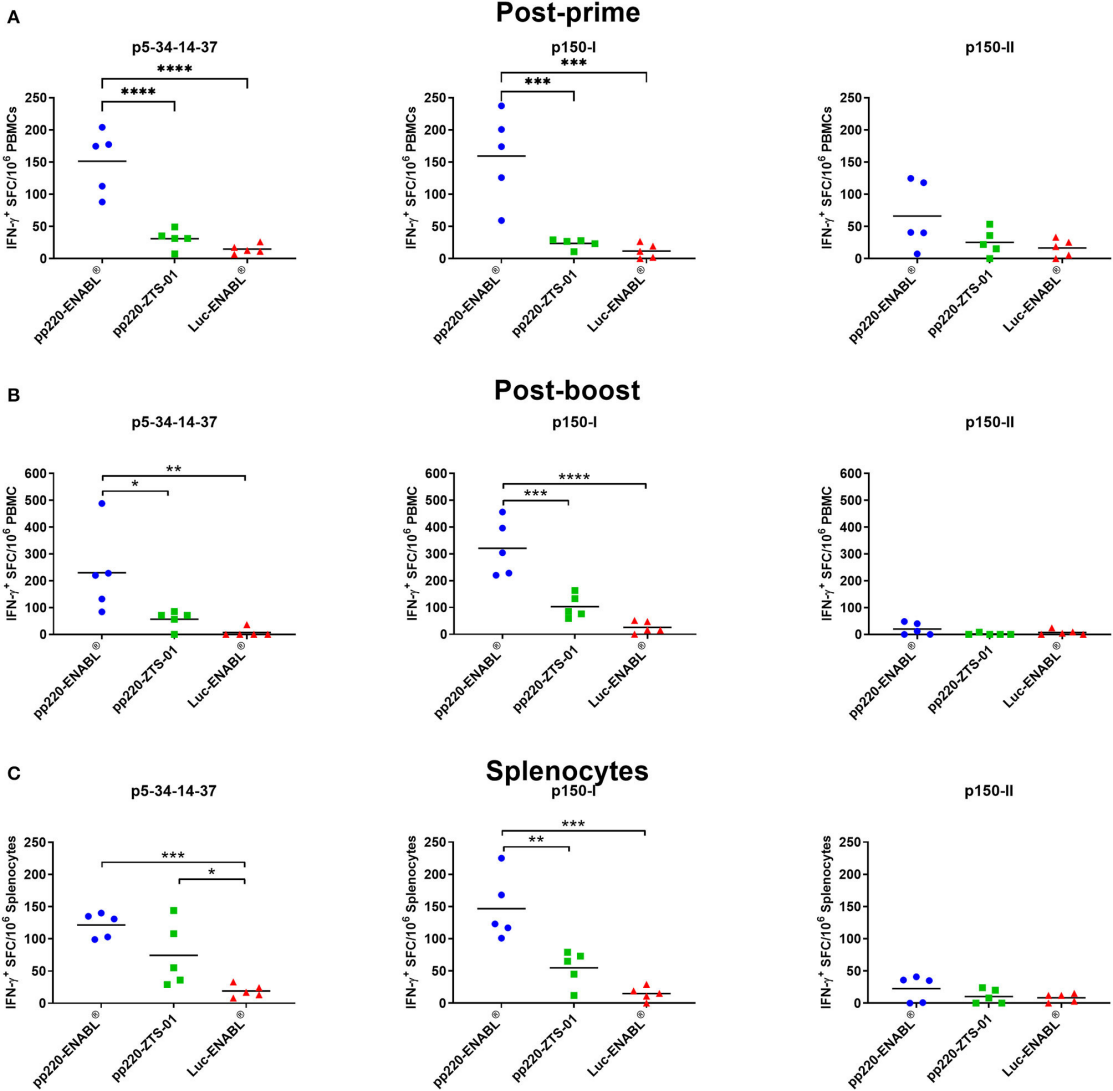
IFN-γ responses against pp220 antigens. p5-34-14-37, p150-I- and p150-II-specific IFN-γ responses were detected by EliSpot assay using PBMCs from **(A)** Two weeks post-priming; **(B)** One-week post-boost; and **(C)** in splenocytes. Data is presented as Spot Forming Cells (SFC)/10^6^ PBMCs or splenocytes. Medium alone served as the negative control, and the data shown is minus media background counts. Mean responses for the groups are denoted by bars, and statistically significant differences between groups are denoted by asterisks (*p < 0.05, **p < 0.01, ***p < 0.001 and ****p < 0.0001).

The Ad-pp220 cocktail formulated in ENABL^®^ adjuvant elicited the highest cellular IFN-γ responses against pp220 antigens in pigs (Figure 7). Post-prime, the mean p5-p34-p14-p37- (*p* < 0.0001) and p150-I-specific (*p* < 0.001) IFN-γ responses detected in PBMCs from the Ad-pp220-ENABL^®^ treatment group were significantly higher than those detected in the Ad-pp220-ZTS-01 treatment group and the Ad-Luc-ENABL^®^ control group (Figure 7A). Pigs in the Ad-pp220-ENABL^®^ treatment group also had the highest post-prime mean IFN-γ response detected in PBMCs against the p150-II antigen. However, no significant differences were detected between the treatment and negative control groups (Figure 7A).

After boosting, p5-p34-p14-p37- (*p* < 0.05) and p150-I-specific (*p* < 0.001) IFN-γ responses in PBMCs were significantly expanded in the Ad-pp220-ENABL^®^-immunized pigs compared to the IFN-γ responses in the Ad-pp220-ZTS-01-immunized pigs (Figure 7B). Mean IFN-γ responses against p5-p34-p14-p37 (*p* < 0.01) and p150-I (*p* < 0.0001) antigens in the Ad-pp220-ENABL^®^ treatment group were also significantly higher than the responses in the negative control Ad-Luc-ENABL^®^ group (Figure 7B). Surprisingly, very low levels of post-boost IFN-γ responses against the p150-II antigen were detected in PBMCs from the Ad-pp220-ENABL^®^-immunized pigs, suggesting that the post-prime responses did not amplify after boosting (Figure 7B). Pigs in the Ad-pp220-ZTS-01 treatment group had no detectable post-boost p150-II-specific responses in PBMCs (Figure 7B).

Consistent recall IFN-γ responses against the p5-p34-p14-p37 and p150-I antigens were detected in the splenocytes from the Ad-pp220-ENABL^®^- and the Ad-pp220-ZTS-01-immunized pigs (Figure 7C). Significantly higher mean p5-p34-p14-p37-specific IFN-γ^+^ splenocytes were recalled in pigs from the Ad-pp220-ENABL^®^ (*p* < 0.001) and the Ad-pp220-ZTS-01 (*p* < 0.05) treatment groups compared to the pigs in the negative control Ad-Luc-ENABL^®^ group (Figure 7C). Mean IFN-γ response recalled in splenocytes against the p150-I antigen in the Ad-pp220-ENABL^®^-immunized pigs was significantly higher than the responses detected in the pigs from the Ad-pp220-ZTS-01 (*p* < 0.01) and the Ad-Luc-ENABL^®^ (*p* < 0.001) groups (Figure 7C). Pigs in the Ad-pp220-ZTS-01 treatment group also had p150-I-specific recall IFN-γ^+^ splenocytes; however, this response was not significantly higher than that detected in the Ad-Luc-ENABL^®^-immunized pigs (Figure 7C). Similar to the post-boost responses detected in PBMCs, very low levels of p150-II-specific IFN-γ^+^ splenocytes were detected in the two treatment groups (Figure 7C).

### Cytotoxic T-Lymphocytes Responses Were Elicited Against pp220 Antigens

**Figure 8:**
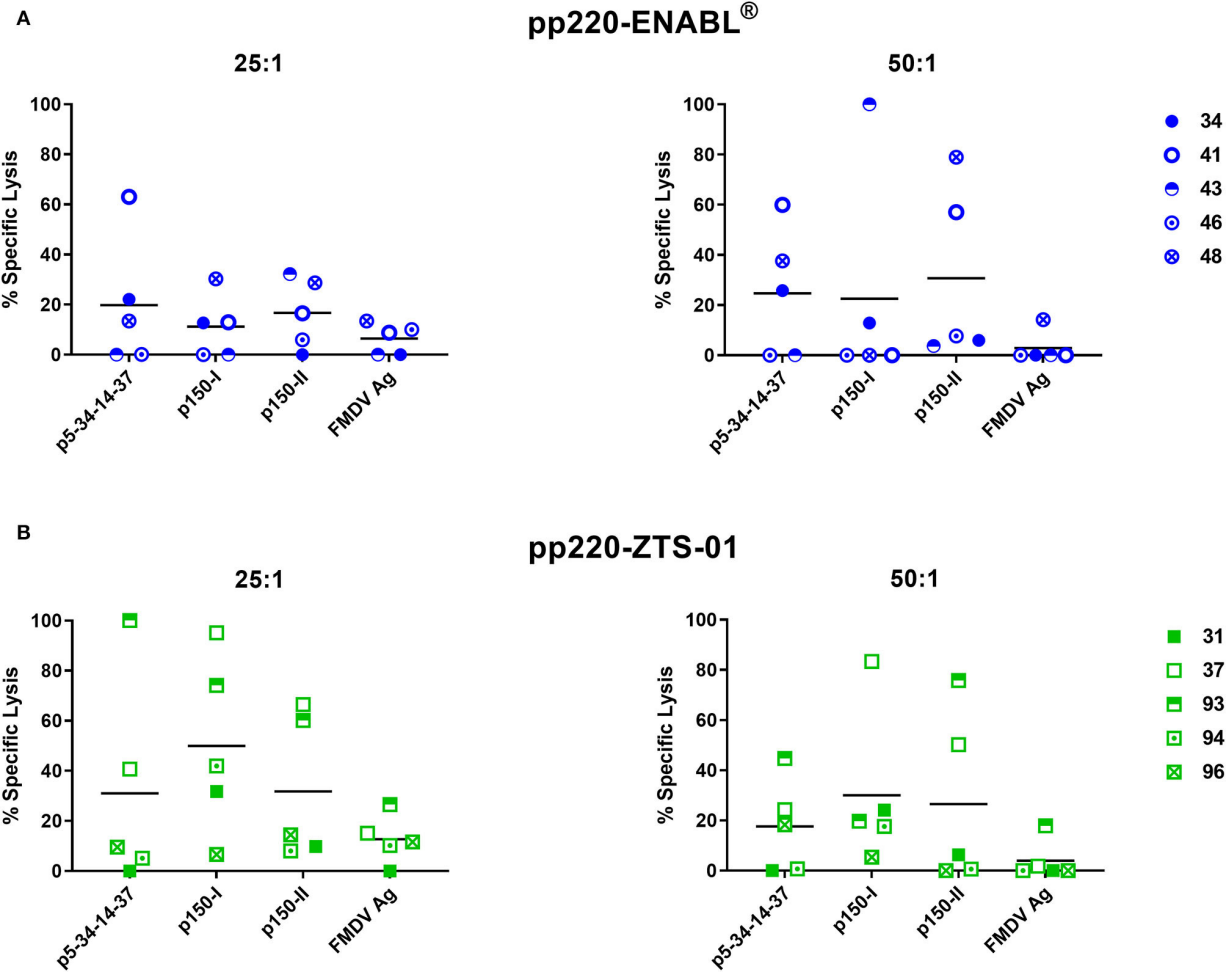
Cytotoxic T-lymphocyte (CTL) responses against the pp220 antigens. At four weeks post-boost, p5-34-14-37-, p150-I-, and p150-II-specific CTL responses in PBMCs collected from **(A)** Ad-pp220-ENABL^®^-immunized; or **(B)** Ad-pp220-ZTS-01-immunized pigs were evaluated at effector to target ratios of 25:1 and 50:1 using the standard ^51^Cr release assay. Data are represented as the percent specific lysis against each antigen and a negative-control FMDV antigen (Ag). Mean responses for each antigen are denoted by bars.

Post-boost, PBMCs collected from the pigs immunized with the Ad-pp220-ENABL^®^ and the Ad-pp220-ZTS-01 formulations showed strong lytic activities against autologous skin fibroblasts expressing the pp220 antigens (Figure 8). Mean background lytic activity against the negative control FMDV antigen in both the treatment groups was at or below 20% (Figure 8). In the Ad-pp220-ENABL^®^ group, 3/5 pigs had p5-p34-p14-p37-specific lytic responses that were higher than the FMDV negative control antigen at both tested effector-to-target ratios (25:1 and 50:1), whereas 3/5 and 2/5 pigs had detectable p150-I-specific lytic responses at the 25:1 and the 50:1 ratio, respectively (Figure 8A). One Ad-pp220-ENABL^®^-immunized pig had a 100% specific lytic response against the p150-I antigen at the 50:1 ratio (Figure 8A). Against the p150-II antigen, 3/5 and 2/5 Ad-pp220-ENABL^®^-immunized pigs had lytic activity above the FMDV negative control antigen at the 25:1 and 50:1 ratio, respectively (Figure 8A).

Lytic activity against the p5-p34-p14-p37 antigen was detected in 2/5 and 3/5 pigs from the Ad-pp220-ZTS-01 treatment group, at the 25:1 and 50:1 ratio, respectively (Figure 8B). For the p150-I antigen, high levels of lytic responses were detected in 4/5 pigs from the Ad-pp220-ZTS-01 treatment group at both the effector-to-target ratios used (Figure 8B). Notably, one Ad-pp220-ZTS-01-immunized pig (number 37) had a consistent high response (>80% specific lysis) against the p150-I antigen at the 25:1 and 50:1 ratio (Figure 8B). In the Ad-pp220-ZTS-01-immunized pigs, 2/5 pigs had consistently high p150-II-specific lytic responses at both the effector-to-target ratios used (Figure 8B). Two pigs (numbers 37 and 93) had consistently high lytic responses against all the three antigens at both the effector-to-target ratios tested (Figure 8B). Overall, the Ad-pp220 cocktail formulated in ZTS-01 adjuvant primed stronger and consistent CTL responses in pigs against all the three pp220 antigens that were detectable at the lower effector to target ratio (Figure 8B).

### IFN-γ-Inducing Nonamer Peptides Were Identified Within ASFV pp220

**Figure 9:**
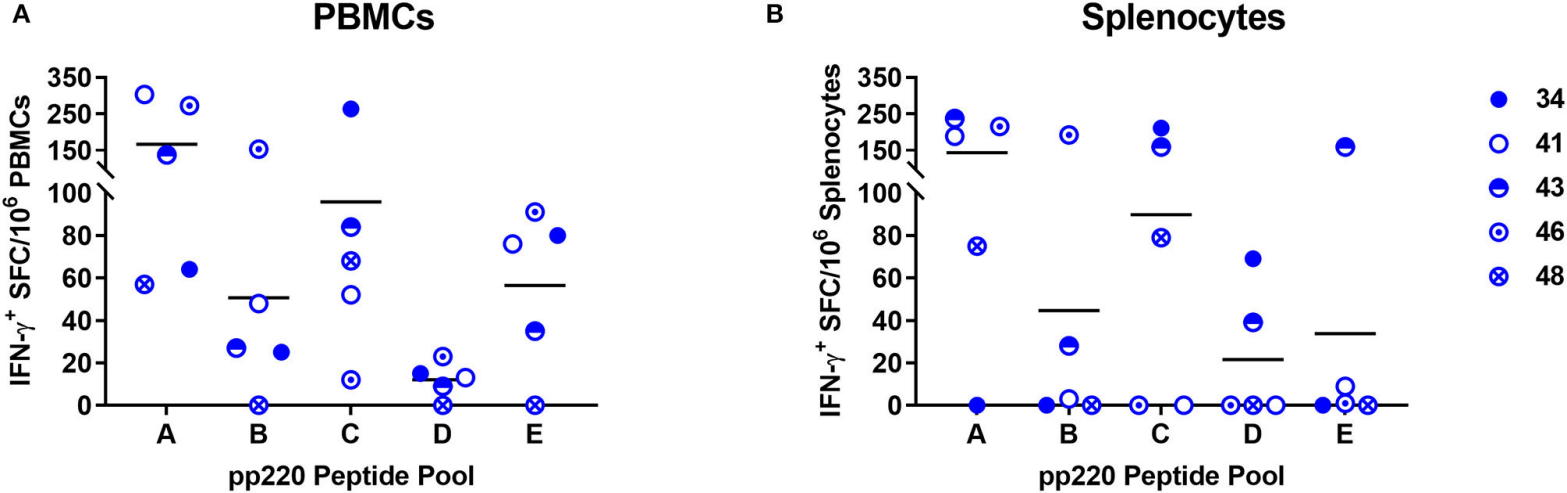
Screening of the predicted pp220 peptide pools. Five pools (Pool A-E) of predicted *SLA I*-binding peptides from the ASFV (Georgia 2007/1) pp220 polyprotein (Table 2) were used to stimulate **(A)** PBMCs or **(B)** splenocytes isolated from pigs immunized with the Ad-pp220-ENABL^®^ formulation, which were then evaluated for antigen-specific recall IFN-γ responses by EliSpot. Data is presented as Spot Forming Cells (SFC)/10^6^ PBMCs or splenocytes for each pig. Medium alone served as the negative control, and the data shown is minus media background counts.

Five pools of predicted *SLA*-I binding nonamer peptides from the ASFV (Georgia 2007/1) pp220 polyprotein (Pools A-E) were screened for their ability to stimulate IFN-γ responses in PBMCs and splenocytes from the pigs immunized with the Ad-pp220-ENABL^®^ formulation since this group had the highest pp220-specific cellular IFN-γ responses (Table 2, Figures 7, 9). The peptide pools A, B, and C stimulated high levels of IFN-γ responses in terminal PBMCs (Figure 9A) as well as splenocytes (Figure 9B) from a majority of the Ad-pp220-ENABL^®^-immunized pigs. Individual peptides from the three selected pools were then evaluated for their ability to stimulate pp220-specific recall IFN-γ^+^ responses in PBMCs and splenocytes from pigs in the Ad-pp220-ENABL^®^ and the Ad-pp220-ZTS-01 treatment groups.

**Figure 10:**
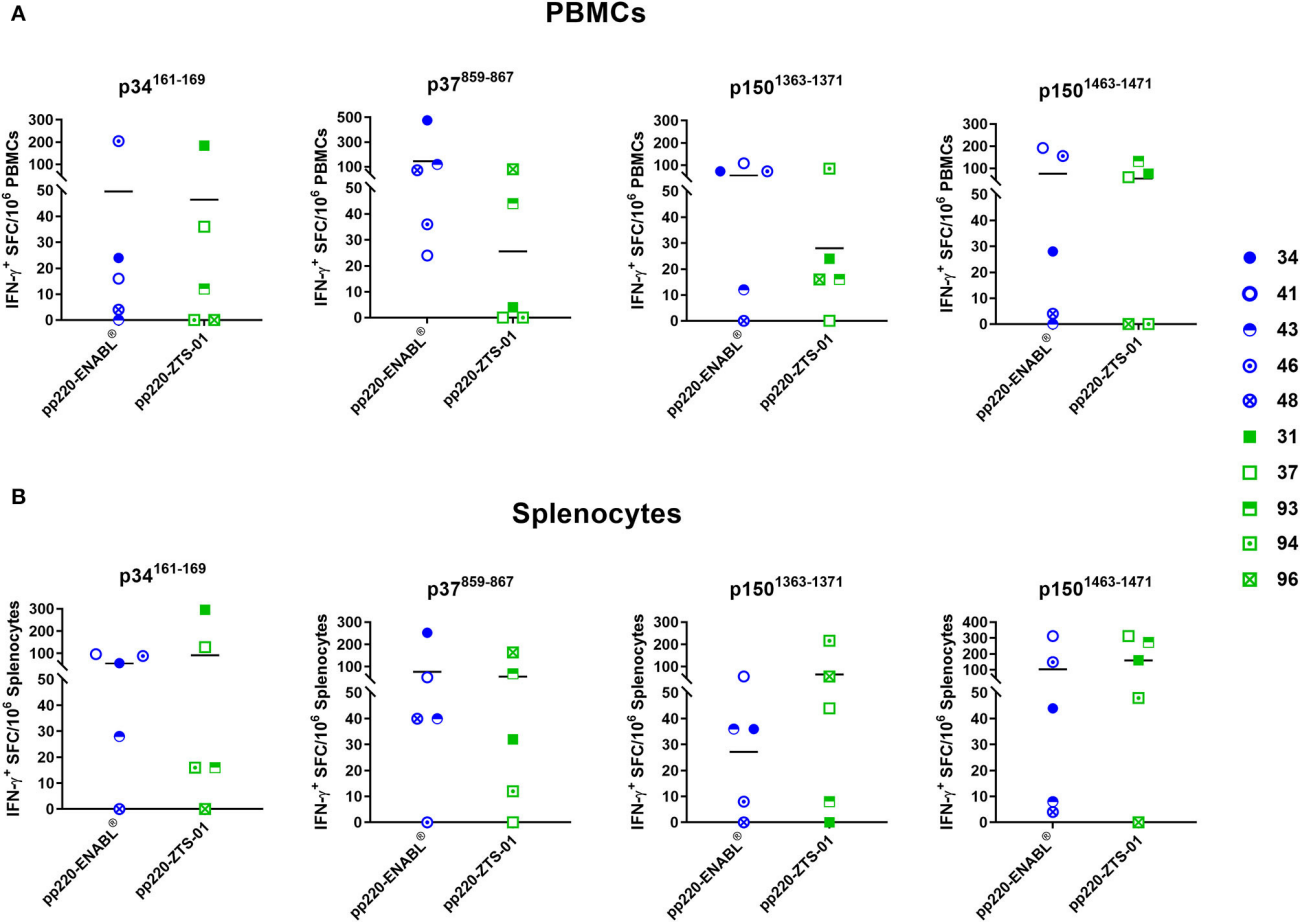
IFN-γ-inducing peptides from ASFV pp220. Four IFN-γ-inducing nanomer peptides from ASFV pp220 that stimulated recall IFN-γ responses in **(A)** PBMCs or **(B)** splenocytes isolated from pigs immunized with the Ad-pp220-ENABL^®^ or the Ad-pp220-ZTS-01 formulation were identified by EliSpot (Table 3). Data for each pig is presented as Spot Forming Cells (SFC)/10^6^ PBMCs or splenocytes. Medium alone served as the negative control and the data shown is minus media background counts. Mean responses for the two groups are denoted by bars.

Four IFN-γ inducing peptides, namely p34^161−169^, p37^859−867^, p150^1363−1371^, and p150^1463−1471^, recalled high numbers of IFN-γ^+^ PBMCs and splenocytes in the pp220-immunized pigs (Figure 10). The first peptide, p34^161−169^, was recognized by PBMCs (Figure 10A) as well as splenocytes (Figure 10B) from 4/5 pigs belonging to the Ad-pp220-ENABL^®^ treatment group. This peptide was also recognized by PBMCs isolated from 3/5 pigs (Figure 10A) and splenocytes from 4/5 pigs (Figure 10B) immunized with the Ad-pp220-ZTS-01 formulation. The second peptide, p37^859−867^, stimulated recall IFN-γ^+^ responses in PBMCs (Figure 10A) and splenocytes (Figure 10B) from 5/5 to 4/5 pigs, respectively, from the Ad-pp220-ENABL^®^ treatment group. This peptide was also recognized by PBMCs from 2/5 pigs (Figure 10A) and splenocytes from 4/5 pigs (Figure 10B) immunized with the Ad-pp220-ZTS-01. The third peptide, p150^1363−1371^, recalled IFN-γ^+^ PBMC and splenocyte responses in 4/5 Ad-pp220-ENABL^®^- and Ad-pp220-ZTS-01-immunized pigs (Figure 10), whereas the fourth peptide, p150^1463−1471^, recalled IFN-γ^+^ PBMCs in 4/5 and 3/5 (Figure 10A) and IFN-γ^+^ splenocytes in 5/5 and 4/5 pigs (Figure 10B) in the Ad-pp220-ENABL^®^ and Ad-pp220-ZTS-01 treatment groups, respectively. Interestingly, these four epitopes are 100% conserved among different ASFV genotypes isolated from domestic pigs, wild boars, warthog, and ticks (Supplementary Figure 1 and Supplemental Table 1). In addition, *in silico* analyses showed that the peptides bind strongly to multiple *SLA*-I alleles (Supplementary Table 2).

## Discussion

The development of efficacious ASFV subunit vaccines is hindered by the lack of definition of the correlates of immune protection and identification of protective antigens (72). Since ASFV mutants can confer immune protection (73, 74), identification of the protective determinants will allow the development of rationally designed prototype subunit vaccines. In this study, pigs were immunized with a cocktail of three adenoviruses encoding the pp220 polyprotein (Ad-p5-p34-p14-p37, Ad-p150-I, and Ad-p150-II). The pp220 polyprotein and pp62 are key components of the ASFV core-shell and the processing of these proteins requires the presence of the major capsid protein p72 (58). The adenovirus cocktail was formulated in adjuvant-induced robust pp220 antigen and wildtype ASFV-specific IgG responses (Figures 5, 6). The endpoint titers of the p5-p34-p14-p37, p150-I, and p150-II antigen-specific IgG titers primed and expanded by both adjuvants are unprecedented and were significantly higher than those detected in the convalescent serum (Figure 5). These outcomes were consistent with previous antibody responses against all the three antigens in sera from pigs immunized with an adenovirus cocktail formulated with BioMize (ENABL) adjuvant. However, strong antibody responses were only observed against p5-p34-p14-p37 in the sera from pigs immunized with the cocktail formulated with ZTS-01 adjuvant (57). Whether anti-pp220 antibodies have a protective function is yet to be determined empirically by a challenge. The role of ASFV-specific antibodies in protection is contentious as neutralization of the virus has been reported, but it may not be mutually exclusive for protection, and this may relate to the target antigens or subtype of immunoglobulin being measured (23, 27, 44, 75). A previous study showed that, even though pigs succumbed to the disease following the challenge, the pigs that had significantly lower antigen-specific IgG responses had better survival rates and lesser clinical scores (57). An immune-mediated enhancement (ADE) of the disease may explain the higher clinical scores observed in the pigs that had high antibody responses than those of the control pigs. Other studies have reported similar findings, and no alternative explanation of the underlying mechanism for the enhanced disease has been outlined (69, 76, 77).

**Table 3:** IFN-γ-inducing nonamer peptides from ASFV pp220 (Georgia 2007/1).

**Peptide ID**	**pp220 Peptide**	**Sequence**	**Predicted *SLA*-I Allele**
26	p34^161−169^	LTHGLRAEY	SLA-2*01:01
38	p37^859−867^	KSMAAKIFI	SLA-2*05:01
11	p150^1363−1371^	HIDKNIIQY	SLA-1*04:01
3	p150^1463−1471^	RVFSRLVFY	SLA-1*02:01

The adenovirus cocktail was formulated in adjuvant-induced strong IFN-γ-secreting cells following intramuscular immunization of pigs. The ENABL-adjuvanted recombinant adenovirus cocktail generated a significantly higher mean number of antigen-specific IFN-γ secreting cells than the ZTS-01 adjuvanted adenovirus cocktail in response to the p5-p34-p14-p37 and the p150-I antigens. However, both adjuvants elicited poor IFN-γ responses against the p150-II antigen. This trend was observed in PBMCs post-priming and post-boost, as well as in splenocytes at study termination (Figure 7). The outcome suggests that the p5-p34-p14-p37 and the p150-I antigens are rich in IFN-γ-inducing epitopes. Strong recall of IFN-γ^+^ responses to adenovirus-vectored ASFV antigens has previously been observed (54, 76). Cytokine response to ASFV infection is highly dependent on the antigen, genotype, level of attenuation, and dose of the virus (27, 28, 78). The level of protection of immunized or recovered pigs following the ASFV challenge is associated with the frequency of ASFV-specific T cells producing IFN-γ (79). The importance of IFN-γ in immune protection is further supported by the demonstration that ASFV antigen-specific CD8^+^ T cells and/or CD4^+^CD8^+^ T cells with cytotoxic ability produce high levels of IFN-γ in response to attenuated virus and can be related to cross-protection between different isolates (24, 42, 49). It has also been shown that immunization of pigs with a pool of eight live-vectored ASFV antigens induced high IFN-γ spot-forming cells and conferred 100% survival of animal post-challenge (80).

Immunization of pigs with the adenovirus cocktail also induced strong CTL responses. However, the cocktail formulated in the ZTS-01 adjuvant primed unprecedented, stronger, and more consistent CTL responses against all the pp220 antigens (Figure 8). This outcome suggests that CTL epitopes are present in the p5-p34-p14-p37, p150-I, and p150-II antigens. This outcome also suggests that these antigens may play a role in eliciting protective immunity, but this will need to be determined empirically. Immunization of pigs with a five-antigen cocktail that included adenoviruses expressing the pp220 antigens conferred protection in 5/9 pigs following challenge (57). Induction of CTLs capable of eliminating infected cells could be the key to complete protection since ASFV-infected cells are cleared specifically by CTLs induced by live-attenuated ASFV (31, 33). It has previously been shown that CD8^+^ cells from pigs that recovered from ASFV infection are cytotoxic to macrophages infected with vaccinia virus expressing the p32 antigen (81). It has also been shown that pigs immunized with an avirulent isolate are immune to challenges with the corresponding virulent strain. However, when such pigs are depleted of CD8^+^ lymphocytes, they develop severe ASF and succumb to the disease upon challenge, suggesting that the CD8^+^ T cells are involved in reducing viremia (49). Several studies support the role of cellular immunity in protection against ASFV, wherein specific T cell responses were present in the absence of measurable antibodies (52, 82).

The IFN-γ EliSpot assay is commonly used to enumerate antigen-specific IFN-γ^+^ T cells following stimulation with one or multiple peptide antigens (83–86), and epitopes presented in the context of MHC I can be identified *ex vivo* (87). A CTL epitope in ASFV p72 antigen was previously mapped using the cumbersome procedure of expressing peptides in a plasmid vector and transfecting target cells (88). Assessment of T-cell responses against CD2v (EP402R) and C-type lectin proteins conducted using 15-mer overlapping peptides showed that 6 of the 132 total predicted peptides resulted in a high frequency of IFN-γ producing cells (89). Bioinformatic platforms in conjunction with EliSpot and CTL assays provide a more practical approach to map key epitopes that may be useful for vaccine development (86, 90–92). The application of *in silico* screening of sequence data combined with experimental methods to develop synthetic vaccines based on defined epitopes presents a theoretical advantage over traditional approaches to vaccine design (93). Multiple IFN-γ^+^-inducing epitopes were identified by screening predicted strong *SLA*-I binding nonamer peptides using the IFN-γ EliSpot assay. Out of the 88 putative epitopes, 4 peptides, namely p34^161−169^, p37^859−867^, p150^1363−1371^, and p150^1463−1471^, recalled very strong IFN-γ^+^ responses in PBMC and splenocytes from pigs immunized with the Ad-pp220 cocktail formulated in either ENABL or ZTS-01 adjuvants (Figure 10). The p34^161−169^ and p37^859−867^ peptides are present in the p5-p34-p14-p37 antigen, whereas the p150^1363−1371^ and p150^1463−1471^ peptides are present in the p150-I antigen, which might explain the poor IFN-γ responses against the p150-II antigen (Figure 7). Thus, multiple T-cell epitopes are present in the pp220 polyprotein that can induce robust IFN-γ^+^ responses in domestic pigs. In addition, the epitopes are 100% conserved among different ASFV genotypes isolated from suids and ticks (Supplementary Figure 1 and Supplementary Table 1). Furthermore, the peptides bind strongly, *in silico*, to multiple *SLA*-I alleles (Supplementary Table 2). Future challenge studies will determine whether these peptides are also CTL epitopes produced from natural infection and whether they play a role in protection. The peptide ITKTFVNNI (number 68 in Table 2) was also previously identified by Bosch-Camos et al. and assessed for immunogenicity in pigs when expressed using a plasmid vector; however, it did not elicit an immune response (94). More recent prediction data indicated peptides IADAINQEF, QIYKTLLEY, and SLYPTQFDY (numbers 2, 4-5 in Table 2) which are highly conserved cytotoxic T-cell epitopes in the ASFV genome (92). The high level of conservation and binding to multiple alleles suggests that the epitopes identified in this study are ideal for inclusion in a prototype subunit vaccine since they have the potential to elicit broad immune responses in outbred pigs. Overall, the use of bioinformatics tools to predict epitopes from the large ASFV proteome followed by empirical identification of relevant determinants that have the potential to contribute to immune protection is a rational subunit vaccine development approach (92, 95).

In conclusion, the results generated in this study demonstrate that the pp220 ASFV polyprotein induced ASFV-specific antibody responses as well as antigen-specific IFN-γ^+^ cellular and CTL responses. These immune responses are important in the clearance of ASFV, given that ASFV-infected cells are cleared by CTLs induced by live-attenuated ASFV and inhibition of IFN-γ has been tied to the persistence and replication of ASFV particles (28, 30, 31, 33, 96). Since attenuated ASFV can confer protection, future studies will entail empirical identification of novel antigens that induce IFN-γ^+^ and CTL responses and evaluation of their protective potential to allow selection of a minimal number of validated antigens for the development of a rationally designed ASFV subunit vaccine.

## Data Availability Statement

The original contributions presented in the study are included in the article/Supplementary Material, further inquiries can be directed to the corresponding authors.

## Ethics Statement

The animal study was reviewed and approved by Texas A&M University Institutional Animal Care and Use Committee (IACUC) (permit# 2009067) approved Animal Use Protocol 2012-59 that follows the regulations, policies, and guidelines put forth by the Animal Welfare Act, United Stated Department of Agriculture (USDA) Animal Care Resource Guide, and the Public Health Service (PHS) Policy on Humane Care and Use of Laboratory Animals.

## Author Contributions

WM, SW, SL, and NS: conceptualization. NS, SL, MZ, HS, JM, and JB: methodology. NS, SL, SW, MZ, RK, TK, and WM: data curation. NS, SL, MZ, RB, SW, and WM: writing, review, and editing. WM: supervision. WM and SW: funding acquisition. All authors have read and agreed to the published version of the manuscript.

## Funding

This research was funded by the Agriculture and Food Research Initiative Competitive Grant No. 2016-67015-25041 from the USDA National Institute of Food and Agriculture and Broad Agency Agreement with the Science and Technology Directorate of the United States Department of Homeland Security under Award Number HSHQDC-11-C-00116, the NBAF Transition Funds, and The APC was funded by the United States Department of Agriculture Animal Plant Health Inspection Service's National Bio- and Agro-defense Facility Scientist Training Program Award Number BG6477.

## Conflict of Interest

The authors declare that the research was conducted in the absence of any commercial or financial relationships that could be construed as a potential conflict of interest.

## Publisher's Note

All claims expressed in this article are solely those of the authors and do not necessarily represent those of their affiliated organizations, or those of the publisher, the editors and the reviewers. Any product that may be evaluated in this article, or claim that may be made by its manufacturer, is not guaranteed or endorsed by the publisher.
