# Supplementary material for: Adenovirus-Vectored African Swine Fever Virus pp220 Induces Robust Antibody, IFN-γ, and CTL Responses in Pigs
Source: Front Vet Sci. 2022 May 31;9:921481. doi: 10.3389/fvets.2022.921481 (PMC9195138; doi:10.3389/fvets.2022.921481)
Supplement: Supplementary file 1 [file Table_1.pdf]

Supplementary Figure 1. Alignment of polyprotein 220 (CP2475) amino acid sequences of the ASFV isolates listed in supplementary Table 1. Conservation of the IFN-γ-inducing nonamer peptides (Table 1) is highlighted in bright green color.

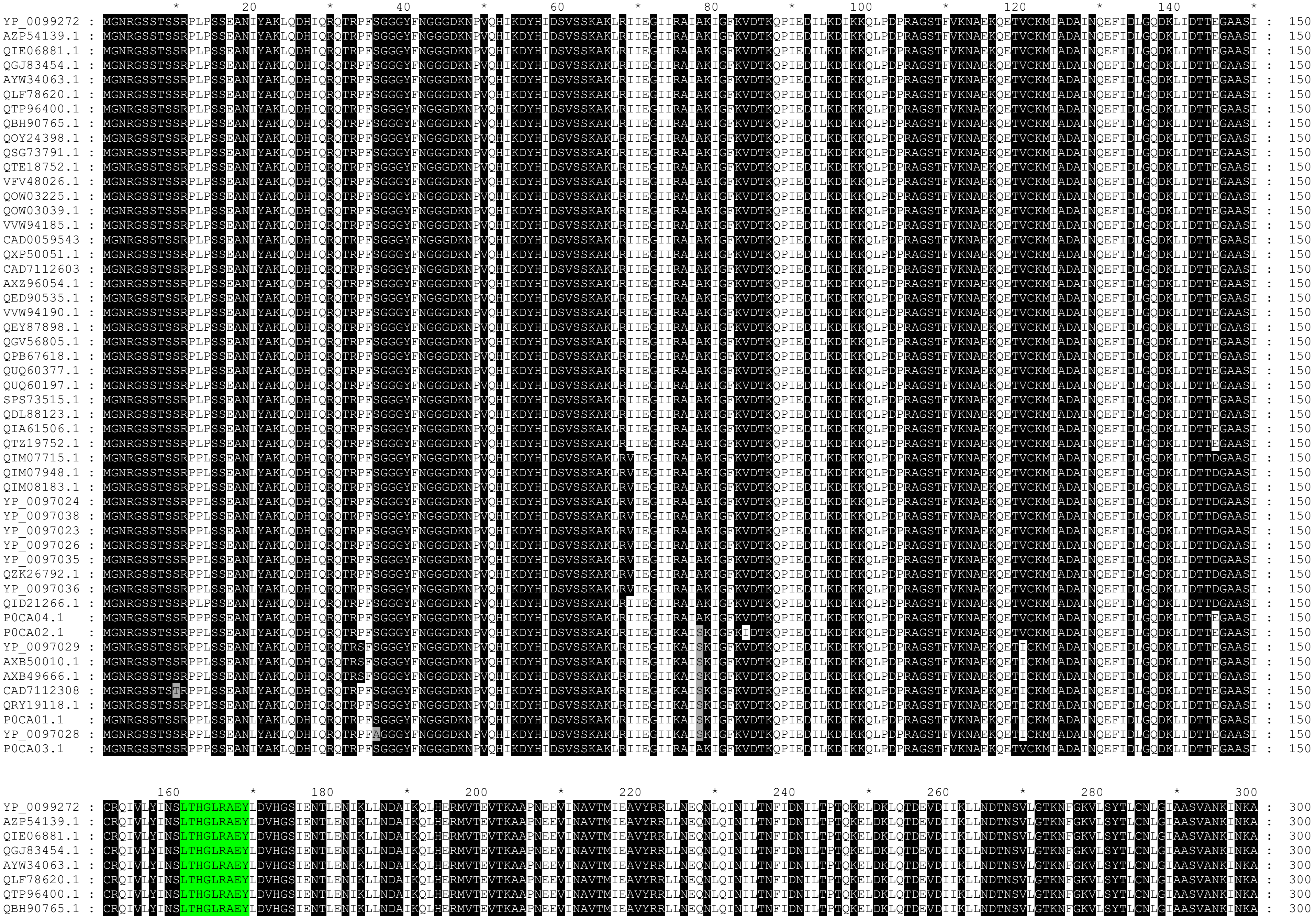

```
QOY24398.1 : CRQIVLYINSLTHGLRAEYLDVHGSIENTLENIKLLNDAIKQLHERMVTEVTKAAPNEEVINAVTMIEAVYRRLLNEQNLQINILTNFIDNILTPTQKELDKLQTDEVDIIKLLNDTNSVLGTKNFGKVLSYTLCNLGIAASVANKINKA : 300
QSG73791.1 : CRQIVLYINSLTHGLRAEYLDVHGSIENTLENIKLLNDAIKQLHERMVTEVTKAAPNEEVINAVTMIEAVYRRLLNEQNLQINILTNFIDNILTPTQKELDKLQTDEVDIIKLLNDTNSVLGTKNFGKVLSYTLCNLGIAASVANKINKA : 300
QTE18752.1 : CRQIVLYINSLTHGLRAEYLDVHGSIENTLENIKLLNDAIKQLHERMVTEVTKAAPNEEVINAVTMIEAVYRRLLNEQNLQINILTNFIDNILTPTQKELDKLQTDEVDIIKLLNDTNSVLGTKNFGKVLSYTLCNLGIAASVANKINKA : 300
VFV48026.1 : CRQIVLYINSLTHGLRAEYLDVHGSIENTLENIKLLNDAIKQLHERMVTEVTKAAPNEEVINAVTMIEAVYRRLLNEQNLQINILTNFIDNILTPTQKELDKLQTDEVDIIKLLNDTNSVLGTKNFGKVLSYTLCNLGIAASVANKINKA : 300
QOW03225.1 : CRQIVLYINSLTHGLRAEYLDVHGSIENTLENIKLLNDAIKQLHERMVTEVTKAAPNEEVINAVTMIEAVYRRLLNEQNLQINILTNFIDNILTPTQKELDKLQTDEVDIIKLLNDTNSVLGTKNFGKVLSYTLCNLGIAASVANKINKA : 300
QOW03039.1 : CRQIVLYINSLTHGLRAEYLDVHGSIENTLENIKLLNDAIKQLHERMVTEVTKAAPNEEVINAVTMIEAVYRRLLNEQNLQINILTNFIDNILTPTQKELDKLQTDEVDIIKLLNDTNSVLGTKNFGKVLSYTLCNLGIAASVANKINKA : 300
VVW94185.1 : CRQIVLYINSLTHGLRAEYLDVHGSIENTLENIKLLNDAIKQLHERMVTEVTKAAPNEEVINAVTMIEAVYRRLLNEQNLQINILTNFIDNILTPTQKELDKLQTDEVDIIKLLNDTNSVLGTKNFGKVLSYTLCNLGIAASVANKINKA : 300
CAD0059543 : CRQIVLYINSLTHGLRAEYLDVHGSIENTLENIKLLNDAIKQLHERMVTEVTKAAPNEEVINAVTMIEAVYRRLLNEQNLQINILTNFIDNILTPTQKELDKLQTDEVDIIKLLNDTNSVLGTKNFGKVLSYTLCNLGIAASVANKINKA : 300
QXP50051.1 : CRQIVLYINSLTHGLRAEYLDVHGSIENTLENIKLLNDAIKQLHERMVTEVTKAAPNEEVINAVTMIEAVYRRLLNEQNLQINILTNFIDNILTPTQKELDKLQTDEVDIIKLLNDTNSVLGTKNFGKVLSYTLCNLGIAASVANKINKA : 300
CAD7112603 : CRQIVLYINSLTHGLRAEYLDVHGSIENTLENIKLLNDAIKQLHERMVTEVTKAAPNEEVINAVTMIEAVYRRLLNEQNLQINILTNFIDNILTPTQKELDKLQTDEVDIIKLLNDTNSVLGTKNFGKVLSYTLCNLGIAASVANKINKA : 300
AXZ96054.1 : CRQIVLYINSLTHGLRAEYLDVHGSIENTLENIKLLNDAIKQLHERMVTEVTKAAPNEEVINAVTMIEAVYRRLLNEQNLQINILTNFIDNILTPTQKELDKLQTDEVDIIKLLNDTNSVLGTKNFGKVLSYTLCNLGIAASVANKINKA : 300
QED90535.1 : CRQIVLYINSLTHGLRAEYLDVHGSIENTLENIKLLNDAIKQLHERMVTEVTKAAPNEEVINAVTMIEAVYRRLLNEQNLQINILTNFIDNILTPTQKELDKLQTDEVDIIKLLNDTNSVLGTKNFGKVLSYTLCNLGIAASVANKINKA : 300
VVW94190.1 : CRQIVLYINSLTHGLRAEYLDVHGSIENTLENIKLLNDAIKQLHERMVTEVTKAAPNEEVINAVTMIEAVYRRLLNEQNLQINILTNFIDNILTPTQKELDKLQTDEVDIIKLLNDTNSVLGTKNFGKVLSYTLCNLGIAASVANKINKA : 300
QEY87898.1 : CRQIVLYINSLTHGLRAEYLDVHGSIENTLENIKLLNDAIKQLHERMVTEVTKAAPNEEVINAVTMIEAVYRRLLNEQNLQINILTNFIDNILTPTQKELDKLQTDEVDIIKLLNDTNSVLGTKNFGKVLSYTLCNLGIAASVANKINKA : 300
QGV56805.1 : CRQIVLYINSLTHGLRAEYLDVHGSIENTLENIKLLNDAIKQLHERMVTEVTKAAPNEEVINAVTMIEAVYRRLLNEQNLQINILTNFIDNILTPTQKELDKLQTDEVDIIKLLNDTNSVLGTKNFGKVLSYTLCNLGIAASVANKINKA : 300
QPB67618.1 : CRQIVLYINSLTHGLRAEYLDVHGSIENTLENIKLLNDAIKQLHERMVTEVTKAAPNEEVINAVTMIEAVYRRLLNEQNLQINILTNFIDNILTPTQKELDKLQTDEVDIIKLLNDTNSVLGTKNFGKVLSYTLCNLGIAASVANKINKA : 300
QUQ60377.1 : CRQIVLYINSLTHGLRAEYLDVHGSIENTLENIKLLNDAIKQLHERMVTEVTKAAPNEEVINAVTMIEAVYRRLLNEQNLQINILTNFIDNILTPTQKELDKLQTDEVDIIKLLNDTNSVLGTKNFGKVLSYTLCNLGIAASVANKINKA : 300
QUQ60197.1 : CRQIVLYINSLTHGLRAEYLDVHGSIENTLENIKLLNDAIKQLHERMVTEVTKAAPNEEVINAVTMIEAVYRRLLNEQNLQINILTNFIDNILTPTQKELDKLQTDEVDIIKLLNDTNSVLGTKNFGKVLSYTLCNLGIAASVANKINKA : 300
SPS73515.1 : CRQIVLYINSLTHGLRAEYLDVHGSIENTLENIKLLNDAIKQLHERMVTEVTKAAPNEEVINAVTMIEAVYRRLLNEQNLQINILTNFIDNILTPTQKELDKLQTDEVDIIKLLNDTNSVLGTKNFGKVLSYTLCNLGIAASVANKINKA : 300
QDL88123.1 : CRQIVLYINSLTHGLRAEYLDVHGSIENTLENIKLLNDAIKQLHERMVTEVTKAAPNEEVINAVTMIEAVYRRLLNEQNLQINILTNFIDNILTPTQKELDKLQTDEVDIIKLLNDTNSVLGTKNFGKVLSYTLCNLGIAASVANKINKA : 300
QIA61506.1 : CRQIVLYINSLTHGLRAEYLDVHGSIENTLENIKLLNDAIKQLHERMVTEVTKAAPNEEVINAVTMIEAVYRRLLNEQNLQINILTNFIDNILTPTQKELDKLQTDEVDIIKLLNDTNSVLGTKNFGKVLSYTLCNLGIAASVANKINKA : 300
QTZ19752.1 : CRQIVLYINSLTHGLRAEYLDVHGSIENTLENIKLLNDAIKQLHERMVTEVTKAAPNEEVINAVTMIEAVYRRLLNEQNLQINILTNFIDNILTPTQKELDKLQTDEVDIIKLLNDTNSVLGTKNFGKVLSYTLCNLGIAASVANKINKA : 300
QIM07715.1 : CRQIVLYINSLTHGLRAEYLDVHGSIENTLENIKLLNDAIKQLHERMVTEVTKAAPNEEVINAVTMIEAVYRRLLNEQNLQINILTNFIDNILTPTQKELDKLQTDEVDIIKLLNDTNSVLGTKNFGKVLSYTLCNLGIAASVANKINKA : 300
QIM07948.1 : CRQIVLYINSLTHGLRAEYLDVHGSIENTLENIKLLNDAIKQLHERMVTEVTKAAPNEEVINAVTMIEAVYRRLLNEQNLQINILTNFIDNILTPTQKELDKLQTDEVDIIKLLNDTNSVLGTKNFGKVLSYTLCNLGIAASVANKINKA : 300
QIM08183.1 : CRQIVLYINSLTHGLRAEYLDVHGSIENTLENIKLLNDAIKQLHERMVTEVTKAAPNEEVINAVTMIEAVYRRLLNEQNLQINILTNFIDNILTPTQKELDKLQTDEVDIIKLLNDTNSVLGTKNFGKVLSYTLCNLGIAASVANKINKA : 300
YP_0097024 : CRQIVLYINSLTHGLRAEYLDVHGSIENTLENIKLLNDAIKQLHERMVTEVTKAAPNEEVINAVTMIEAVYRRLLNEQNLQINILTNFIDNILTPTQKELDKLQTDEVDIIKLLNDTNSVLGTKNFGKVLSYTLCNLGIAASVANKINKA : 300
YP_0097038 : CRQIVLYINSLTHGLRAEYLDVHGSIENTLENIKLLNDAIKQLHERMVTEVTKAAPNEEVINAVTMIEAVYRRLLNEQNLQINILTNFIDNILTPTQKELDKLQTDEVDIIKLLNDTNSVLGTKNFGKVLSYTLCNLGIAASVANKINKA : 300
YP_0097023 : CRQIVLYINSLTHGLRAEYLDVHGSIENTLENIKLLNDAIKQLHERMVTEVTKAAPNEEVINAVTMIEAVYRRLLNEQNLQINILTNFIDNILTPTQKELDKLQTDEVDIIKLLNDTNSVLGTKNFGKVLSYTLCNLGIAASVANKINKA : 300
YP_0097026 : CRQIVLYINSLTHGLRAEYLDVHGSIENTLENIKLLNDAIKQLHERMVTEVTKAAPNEEVINAVTMIEAVYRRLLNEQNLQINILTNFIDNILTPTQKELDKLQTDEVDIIKLLNDTNSVLGTKNFGKVLSYTLCNLGIAASVANKINKA : 300
YP_0097035 : CRQIVLYINSLTHGLRAEYLDVHGSIENTLENIKLLNDAIKQLHERMVTEVTKAAPNEEVINAVTMIEAVYRRLLNEQNLQINILTNFIDNILTPTQKELDKLQTDEVDIIKLLNDTNSVLGTKNFGKVLSYTLCNLGIAASVANKINKA : 300
QZK26792.1 : CRQIVLYINSLTHGLRAEYLDVHGSIENTLENIKLLNDAIKQLHERMVTEVTKAAPNEEVINAVTMIEAVYRRLLNEQNLQINILTNFIDNILTPTQKELDKLQTDEVDIIKLLNDTNSVLGTKNFGKVLSYTLCNLGIAASVANKINKA : 300
YP_0097036 : CRQIVLYINSLTHGLRAEYLDVHGSIENTLENIKLLNDAIKQLHERMVTEVTKAAPNEEVINAVTMIEAVYRRLLNEQNLQINILTNFIDNILTPTQKELDKLQTDEVDIIKLLNDTNSVLGTKNFGKVLSYTLCNLGIAASVANKINKA : 300
QID21266.1 : CRQIVLYINSLTHGLRAEYLDVHGSIENTLENIKLLNDAIKQLHERMVTEVTKAPPNEEVINAVTMIEAVYRRLLNEQNLQINILTNFIDNILTPTQKELDKLQTDEVDIIKLLNDTNSVLGTKNFGKVLSYTLCNLGIAASVANKINKA : 300
P0CA04.1   : CRQIVLYINSLTHGLRAEYLDVHGSIENTLENIKLLNDAIKQLHERMVTEVTKAAPNEEVINAVTMIEAVYRRLLNEQNLQINILTNFIDNILTPTQKELDKLQTDEVDIIKLLNDTNSVLGTKNFGKVLSYTLCNLGIAASVANKINKA : 300
P0CA02.1   : CRQIVLYINSLTHGLRAEYLDVHGSIENTLENIKLLNDAIKQLHERMVTEVTKAAPNEEVINAVTMIEAVYRRLLNEQNLQISILTNFIDNILTPTQKELDKLQTDEVDIIKLLNDTNSVLGTKNFGKVLSYTLCNLGIAASVANKINKA : 300
YP_0097029 : CRQIVLYINSLTHGLRAEYLDVHGSIENTLENIKLLSDAIKQLHERMVTEVTKAAPNEEVINAVTMIEAVYRRLLNEQNLQINILTNFIDNILTPTQKELDKLKTDEVDIIKILNDTNSVLGTKNFGKVLSYTLCNLGIAATVANKINKA : 300
AXB50010.1 : CRQIVLYINSLTHGLRAEYLDVHGSIENTLENIKLLSDAIKQLHERMVTEVTKAAPNEEVINAVTMIEAVYRRLLNEQNLQINILTNFIDNILTPTQKELDKLKTDEVDIIKILNDTNSVLGTKNFGKVLSYTLCNLGIAATVANKINKA : 300
AXB49666.1 : CRQIVLYINSLTHGLRAEYLDVHGSIENTLENIKLLSDAIKQLHERMVTEVTKAAPNEEVINAVTMIEAVYRRLLNEQNLQINILTNFIDNILTPTQKELDKLKTDEVDIIKILNDTNSVLGTKNFGKVLSYTLCNLGIAATVANKINKA : 300
CAD7112308 : CRQIVLYINSLTHGLRAEYLDVHGSIENTLENIKLLSDAIKQLHERMVTEVTKAAPNEEVINAVTMIEAVYRRLLNEQNLQINILTNFIDNILTPTQKELDKLKTDEVDIIKILNDTNSVLGTKNFGKVLSYTLCNLGIAATVANKINKA : 300
QRY19118.1 : CRQIVLYINSLTHGLRAEYLDVHGSIENTLENIKLLSDAIKQLHERMVTEVTKAAPNEEVINAVTMIEAVYRRLLNEQNLQINILTNFIDNILTPTQKELDKLKTDEVDIIKILNDTNSVLGTKNFGKVLSYTLCNLGIAATVANKINKA : 300
P0CA01.1   : CRQIVLYINSLTHGLRAEYLDVHGSIENTLENIKLLSDAIKQLHERMVTEVTKAAPNEEVINAVTMIEAVYRRLLNEQNLQINILTNFIDNILTPTQKELDKLKTDEVDIIKILNDTNSVLGTKNFGKVLSYTLCNLGIAATVANKINKA : 300
YP_0097028 : CRQIVLYINSLTHGLRAEYLDVHGSIENTLENIKLLSDAIKQLHERMVTEVTKAAPNEEVINAVTMIEAVYRRLLNEQNLQINILTNFIDNILTPTQKELDKLKTDEVDIIKILNDTNSVLGTKNFGKVLSYTLCNLGIAATVANKINKA : 300
P0CA03.1   : CRQIVLYINSLTHGLRAEYLDVHGSIENTLENIKLLNDAIKQLHERMVTEVTKAAPNEEVINAVTMIEAVYRRLLNEQNLQINILTNFIDNILTPTQKELDKLQTDEVDIIKLLNDTNSVLGTKNFGKVLSYTLCNLGIAASVANKINKA : 300

                      *       320         *       340         *       360         *       380         *       400         *       420         *       440         *
YP_0099272 : LQKVGLKVEQYLQSKNWAEFDKELDLKRFSGLVSAENIAEFEKAVNLLRQTFNERHKILENSCAKKGGDEEKTPLDRRIEAQRLDRKHILMEFLNKSTQAYNDFLENVKKIGIKLVKEIALTPNITRLRDALSRINDMGTIALDLSLIGF : 450
AZP54139.1 : LQKVGLKVEQYLQSKNWAEFDKELDLKRFSGLVSAENIAEFEKAVNLLRQTFNERHKILENSCAKKGGDEEKTPLDRRIEAQRLDRKHILMEFLNKSTQAYNDFLENVKKIGIKLVKEIALTPNITRLRDALSRINDMGTIALDLSLIGF : 450
QIE06881.1 : LQKVGLKVEQYLQSKNWAEFDKELDLKRFSGLVSAENIAEFEKAVNLLRQTFNERHKILENSCAKKGGDEEKTPLDRRIEAQRLDRKHILMEFLNKSTQAYNDFLENVKKIGIKLVKEIALTPNITRLRDALSRINDMGTIALDLSLIGF : 450
QGJ83454.1 : LQKVGLKVEQYLQSKNWAEFDKELDLKRFSGLVSAENIAEFEKAVNLLRQTFNERHKILENSCAKKGGDEEKTPLDRRIEAQRLDRKHILMEFLNKSTQAYNDFLENVKKIGIKLVKEIALTPNITRLRDALSRINDMGTIALDLSLIGF : 450
AYW34063.1 : LQKVGLKVEQYLQSKNWAEFDKELDLKRFSGLVSAENIAEFEKAVNLLRQTFNERHKILENSCAKKGGDEEKTPLDRRIEAQRLDRKHILMEFLNKSTQAYNDFLENVKKIGIKLVKEIALTPNITRLRDALSRINDMGTIALDLSLIGF : 450
QLF78620.1 : LQKVGLKVEQYLQSKNWAEFDKELDLKRFSGLVSAENIAEFEKAVNLLRQTFNERHKILENSCAKKGGDEEKTPLDRRIEAQRLDRKHILMEFLNKSTQAYNDFLENVKKIGIKLVKEIALTPNITRLRDALSRINDMGTIALDLSLIGF : 450
QTP96400.1 : LQKVGLKVEQYLQSKNWAEFDKELDLKRFSGLVSAENIAEFEKAVNLLRQTFNERHKILENSCAKKGGDEEKTPLDRRIEAQRLDRKHILMEFLNKSTQAYNDFLENVKKIGIKLVKEIALTPNITRLRDALSRINDMGTIALDLSLIGF : 450
QBH90765.1 : LQKVGLKVEQYLQSKNWAEFDKELDLKRFSGLVSAENIAEFEKAVNLLRQTFNERHKILENSCAKKGGDEEKTPLDRRIEAQRLDRKHILMEFLNKSTQAYNDFLENVKKIGIKLVKEIALTPNITRLRDALSRINDMGTIALDLSLIGF : 450
QOY24398.1 : LQKVGLKVEQYLQSKNWAEFDKELDLKRFSGLVSAENIAEFEKAVNLLRQTFNERHKILENSCAKKGGDEEKTPLDRRIEAQRLDRKHILMEFLNKSTQAYNDFLENVKKIGIKLVKEIALTPNITRLRDALSRINDMGTIALDLSLIGF : 450
QSG73791.1 : LQKVGLKVEQYLQSKNWAEFDKELDLKRFSGLVSAENIAEFEKAVNLLRQTFNERHKILENSCAKKGGDEEKTPLDRRIEAQRLDRKHILMEFLNKSTQAYNDFLENVKKIGIKLVKEIALTPNITRLRDALSRINDMGTIALDLSLIGF : 450
QTE18752.1 : LQKVGLKVEQYLQSKNWAEFDKELDLKRFSGLVSAENIAEFEKAVNLLRQTFNERHKILENSCAKKGGDEEKTPLDRRIEAQRLDRKHILMEFLNKSTQAYNDFLENVKKIGIKLVKEIALTPNITRLRDALSRINDMGTIALDLSLIGF : 450
VFV48026.1 : LQKVGLKVEQYLQSKNWAEFDKELDLKRFSGLVSAENIAEFEKAVNLLRQTFNERHKILENSCAKKGGDEEKTPLDRRIEAQRLDRKHILMEFLNKSTQAYNDFLENVKKIGIKLVKEIALTPNITRLRDALSRINDMGTIALDLSLIGF : 450
QOW03225.1 : LQKVGLKVEQYLQSKNWAEFDKELDLKRFSGLVSAENIAEFEKAVNLLRQTFNERHKILENSCAKKGGDEEKTPLDRRIEAQRLDRKHILMEFLNKSTQAYNDFLENVKKIGIKLVKEIALTPNITRLRDALSRINDMGTIALDLSLIGF : 450
QOW03039.1 : LQKVGLKVEQYLQSKNWAEFDKELDLKRFSGLVSAENIAEFEKAVNLLRQTFNERHKILENSCAKKGGDEEKTPLDRRIEAQRLDRKHILMEFLNKSTQAYNDFLENVKKIGIKLVKEIALTPNITRLRDALSRINDMGTIALDLSLIGF : 450
VVW94185.1 : LQKVGLKVEQYLQSKNWAEFDKELDLKRFSGLVSAENIAEFEKAVNLLRQTFNERHKILENSCAKKGGDEEKTPLDRRIEAQRLDRKHILMEFLNKSTQAYNDFLENVKKIGIKLVKEIALTPNITRLRDALSRINDMGTIALDLSLIGF : 450
CAD0059543 : LQKVGLKVEQYLQSKNWAEFDKELDLKRFSGLVSAENIAEFEKAVNLLRQTFNERHKILENSCAKKGGDEEKTPLDRRIEAQRLDRKHILMEFLNKSTQAYNDFLENVKKIGIKLVKEIALTPNITRLRDALSRINDMGTIALDLSLIGF : 450
QXP50051.1 : LQKVGLKVEQYLQSKNWAEFDKELDLKRFSGLVSAENIAEFEKAVNLLRQTFNERHKILENSCAKKGGDEEKTPLDRRIEAQRLDRKHILMEFLNKSTQAYNDFLENVKKIGIKLVKEIALTPNITRLRDALSRINDMGTIALDLSLIGF : 450
CAD7112603 : LQKVGLKVEQYLQSKNWAEFDKELDLKRFSGLVSAENIAEFEKAVNLLRQTFNERHKILENSCAKKGGDEEKTPLDRRIEAQRLDRKHILMEFLNKSTQAYNDFLENVKKIGIKLVKEIALTPNITRLRDALSRINDMGTIALDLSLIGF : 450
AXZ96054.1 : LQKVGLKVEQYLQSKNWAEFDKELDLKRFSGLVSAENIAEFEKAVNLLRQTFNERHKILENSCAKKGGDEEKTPLDRRIEAQRLDRKHILMEFLNKSTQAYNDFLENVKKIGIKLVKEIALTPNITRLRDALSRINDMGTIALDLSLIGF : 450
QED90535.1 : LQKVGLKVEQYLQSKNWAEFDKELDLKRFSGLVSAENIAEFEKAVNLLRQTFNERHKILENSCAKKGGDEEKTPLDRRIEAQRLDRKHILMEFLNKSTQAYNDFLENVKKIGIKLVKEIALTPNITRLRDALSRINDMGTIALDLSLIGF : 450
VVW94190.1 : LQKVGLKVEQYLQSKNWAEFDKELDLKRFSGLVSAENIAEFEKAVNLLRQTFNERHKILENSCAKKGGDEEKTPLDRRIEAQRLDRKHILMEFLNKSTQAYNDFLENVKKIGIKLVKEIALTPNITRLRDALSRINDMGTIALDLSLIGF : 450
QEY87898.1 : LQKVGLKVEQYLQSKNWAEFDKELDLKRFSGLVSAENIAEFEKAVNLLRQTFNERHKILENSCAKKGGDEEKTPLDRRIEAQRLDRKHILMEFLNKSTQAYNDFLENVKKIGIKLVKEIALTPNITRLRDALSRINDMGTIALDLSLIGF : 450
```

```
QGV56805.1 : LQKVGLKVEQYLQSKNWAEFDKELDLKRFSGLVSAENIAEFEKAVNLLRQTFNERHKILENSCAKKGGDEEKTPLDRRIEAQRLDRKHILMEFLNKSTQAYNDFLENVKKIGIKLVKEIALTPNITRLRDALSRINDMGTIALDLSLIGF :  450
QPB67618.1 : LQKVGLKVEQYLQSKNWAEFDKELDLKRFSGLVSAENIAEFEKAVNLLRQTFNERHKILENSCAKKGGDEEKTPLDRRIEAQRLDRKHILMEFLNKSTQAYNDFLENVKKIGIKLVKEIALTPNITRLRDALSRINDMGTIALDLSLIGF :  450
QUQ60377.1 : LQKVGLKVEQYLQSKNWAEFDKELDLKRFSGLVSAENIAEFEKAVNLLRQTFNERHKILENSCAKKGGDEEKTPLDRRIEAQRLDRKHILMEFLNKSTQAYNDFLENVKKIGIKLVKEIALTPNITRLRDALSRINDMGTIALDLSLIGF :  450
QUQ60197.1 : LQKVGLKVEQYLQSKNWAEFDKELDLKRFSGLVSAENIAEFEKAVNLLRQTFNERHKILENSCAKKGGDEEKTPLDRRIEAQRLDRKHILMEFLNKSTQAYNDFLENVKKIGIKLVKEIALTPNITRLRDALSRINDMGTIALDLSLIGF :  450
SPS73515.1 : LQKVGLKVEQYLQSKNWAEFDKELDLKRFSGLVSAENIAEFEKAVNLLRQTFNERHKILENSCAKKGGDEEKTPLDRRIEAQRLDRKHILMEFLNKSTQAYNDFLENVKKIGIKLVKEIALTPNITRLRDALSRINDMGTIALDLSLIGF :  450
QDL88123.1 : LQKVGLKVEQYLQSKNWAEFDKELDLKRFSGLVSAENIAEFEKAVNLLRQTFNERHKILENSCAKKGGDEEKTPLDRRIEAQRLDRKHILMEFLNKSTQAYNDFLENVKKIGIKLVKEIALTPNITRLRDALSRINDMGTIALDLSLIGF :  450
QIA61506.1 : LQKVGLKVEQYLQSKNWAEFDKELDLKRFSGLVSAENIAEFEKAVNLLRQTFNERHKILENSCAKKGGDEEKTPLDRRIEAQRLDRKHILMEFLNKSTQAYNDFLENVKKIGIKLVKEIALTPNITRLRDALSRINDMGTIALDLSLIGF :  450
QTZ19752.1 : LQKVGLKVEQYLQSKNWAEFDKELDLKRFSGLVSAENIAEFEKAVNLLRQTFNERHKILENSCAKKGGDEEKTPLDRRIEAQRLDRKHILMEFLNKSTQAYNDFLENVKKIGIKLVKEIALTPNITRLRDALSRINDMGTIALDLSLIGF :  450
QIM07715.1 : LQKVGLKVEQYLQSKNWAEFDKELDLKRFSGLVSAENIAEFEKAVNLLRQTFNERHKILENSCAKKGGDEEKTPLDRRIEAQRLDRKHILMEFLNKSTQAYNDFLENVKKIGIKLVKEIALTPNITRLRDALSRINDMGTIALDLSLIGF :  450
QIM07948.1 : LQKVGLKVEQYLQSKNWAEFDKELDLKRFSGLVSAENIAEFEKAVNLLRQTFNERHKILENSCAKKGGDEEKTPLDRRIEAQRLDRKHILMEFLNKSTQAYNDFLENVKKIGIKLVKEIALTPNITRLRDALSRINDMGTIALDLSLIGF :  450
QIM08183.1 : LQKVGLKVEQYLQSKNWAEFDKELDLKRFSGLVSAENIAEFEKAVNLLRQTFNERHKILENSCAKKGGDEEKTPLDRRIEAQRLDRKHILMEFLNKSTQAYNDFLENVKKIGIKLVKEIALTPNITRLRDALSRINDMGTIALDLSLIGF :  450
YP_0097024 : LQKVGLKVEQYLQSKNWAEFDKELDLKRFSGLVSAENIAEFEKAVNLLRQTFNERHKILENSCAKKGGDEEKTPLDRRIEAQRLDRKHILMEFLNKSTQAYNDFLENVKKIGIKLVKEIALTPNITRLRDALSRINDMGTIALDLSLIGF :  450
YP_0097038 : LQKVGLKVEQYLQSKNWAEFDKELDLKRFSGLVSAENIAEFEKAVNLLRQTFNERHKILENSCAKKGGDEEKTPLDRRIEAQRLDRKHILMEFLNKSTQAYNDFLENVKKIGIKLVKEIALTPNITRLRDALSRINDMGTIALDLSLIGF :  450
YP_0097023 : LQKVGLKVEQYLQSKNWAEFDKELDLKRFSGLVSAENIAEFEKAVNLLRQTFNERHKILENSCAKKGGDEEKTPLDRRIEAQRLDRKHILMEFLNKSTQAYNDFLENVKKIGIKLVKEIALTPNITRLRDALSRINDMGTIALDLSLIGF :  450
YP_0097026 : LQKVGLKVEQYLQSKNWAEFDKELDLKRFSGLVSAENIAEFEKAVNLLRQTFNERHKILENSCAKKGGDEEKTPLDRRIEAQRLDRKHILMEFLNKSTQAYNDFLENVKKIGIKLVKEIALTPNITRLRDALSRINDMGTIALDLSLIGF :  450
YP_0097035 : LQKVGLKVEQYLQSKNWAEFDKELDLKRFSGLVSAENIAEFEKAVNLLRQTFNERHKILENSCAKKGGDEEKTPLDRRIEAQRLDRKHILMEFLNKSTQAYNDFLENVKKIGIKLVKEIALTPNITRLRDALSRINDMGTIALDLSLIGF :  450
QZK26792.1 : LQKVGLKVEQYLQSKNWAEFDKELDLKRFSGLVSAENIAEFEKAVNLLRQTFNERHKILENSCAKKGGDEEKTPLDRRIEAQRLDRKHILMEFLNKSTQAYNDFLENVKKIGIKLVKEIALTPNITRLRDALSRINDMGTIALDLSLIGF :  450
YP_0097036 : LQKVGLKVEQYLQSKNWAEFDKELDLKRFSGLVSAENIAEFEKAVNLLRQTFNERHKILENSCAKKGGDEEKTPLDRRIEAQRLDRKHILMEFLNKSTQAYNDFLENVKKIGIKLVKEIALTPNITRLRDALSRINDMGTIALDLSLIGF :  450
QID21266.1 : LQKVGLKVEQYLQSKNWAEFDKELDLKRFSGLVSAENIAEFEKAVNLLRQTFNERHKILENSCAKKGGDEEKTPLDRRIEAQRLDRKHILMEFLNKSTQAYNDFLENVKKIGIKLVKEIALTPNITRLRDALSRINDMGTIALDLSLIGF :  450
P0CA04.1   : LQKVGLKVEQYLQSKNWAEFDKELDLKRFSGLVSAENIAEFEKAVNLLRQTFNERHKILENSCAKKGGDEEKTPLDRRIEAQRLDRKHILMEFLNKSTQAYNDFLENVKKIGIKLVKEIALTPNITRLRDALSRINDMGTIALDLSLIGF :  450
P0CA02.1   : LQRVGLKVEQYLQSKNWAEFDKELDLKRFSGLVSAENIAEFEKAVNLLRQTFNERHKILENNCAKKGGDEVKTPLDKRIEAQRLDRKHILMEFLNKSTQAYNDFLENVKKIGIKLVKEIALTPNITRLRDALSRINDMGTIALDLSLIGF :  450
YP_0097029 : LQRVGLKVEQYLHSKNWAEFDKELDLKRFSGLVSAENIAEFEKAVNLLRQTFNERHKILENNCAKKGGDEEKTPLDKRMEAQRLDRKHILMEFLNKSTQAYNDFLENVKKIGMKLVKEIALTPNITKLRDALSRINDMGTIALDLSLIGF :  450
AXB50010.1 : LQRVGLKVEQYLHSKNWAEFDKELDLKRFSGLVSAENIAEFEKAVNLLRQTFNERHKILENNCAKKGGDEEKTPLDKRMEAQRLDRKHILMEFLNKSTQAYNDFLENVKKIGMKLVKEIALTPNITKLRDALSRINDMGTIALDLSLIGF :  450
AXB49666.1 : LQRVGLKVEQYLHSKNWAEFDKELDLKRFSGLVSAENIAEFEKAVNLLRQTFNERHKILENNCAKKGGDEEKTPLDKRMEAQRLDRKHILMEFLNKSTQAYNDFLENVKKIGMKLVKEIALTPNITKLRDALSRINDMGTIALDLSLIGF :  450
CAD7112308 : LQRVGLKVEQYLHSKNWAEFDKELDLKRFSGLVSAENIAEFEKAVNLLRQTFNERHKILENNCAKKGGDEEKTPLDKRMEAQRLDRKHILMEFLNKSTQAYNDFLENVKKIGMKLVKEIALTPNITKLRDALSRINDMGTIALDLSLIGF :  450
QRY19118.1 : LQRVGLKVEQYLHSKNWAEFDKELDLKKFSGLVSAENIAEFEKAVNLLRQTFNERHKILENNCAKKGGDEEKTPLDKRMEAQRLDRKHILMEFLNKSTQAYNDFLENVKKIGMKLVKEIALTPNITKLRDALSRINDMGTIALDLSLIGF :  450
P0CA01.1   : LQRVGLKVEQYLHSKNWAEFDKELDLKRFSGLVSAENIAEFEKAVNLLRQTFNERHKILENNCAKKGGDGEKTPLDKRMEAQRLDRKHILMEFLNKSTQAYNDFLENVKKIGMKLVKEIALTPNITKLRDALSRINDMGTIALDLSLIGF :  450
YP_0097028 : LQRVGLKVEQYLHSKNWAEFDKELDLKRFSGLVSAENIAEFEKAVNLLRQTFNERHKILENNCAKKGGDEEKTPLDKRMEAQRLDRKHILMEFLNKSTQAYNDFLENVKKIGMKLVKEIALTPNITKLRDALSRINDMGTIALDLSLIGF :  450
P0CA03.1   : LQKVGLKVEQYLQSKNWEEFDKELDLKRFSGLVSAENIAEFEKAVNLLRQTFNERHKILENSCAKKGGDEEKTPLDRRIEAQRLDRKHILMEFLNKSTQAYNDFLENVKKIGIKLVKEIALTPNITRLRDALSRINDMGTIALDLSLIGF :  450
                                                                                                                                                      l

                     460         *       480         *       500         *       520         *       540         *       560         *       580         *       600
YP_0099272 : YTNAAAREERETFLTQLTLVKNVLEEISKTDPNFKNLYDSCSRLLQIIDFYTDIVQKKYGGEEDCECTRVGGAALTVEELGLSKAARSQVDLNQAINTFMYYYYVAQIYSNLTHNKQEFQSYEENYATILGDAIAGRLMQLDTEKNARIN :  600
AZP54139.1 : YTNAAAREERETFLTQLTLVKNVLEEISKTDPNFKNLYDSCSRLLQIIDFYTDIVQKKYGGEEDCECTRVGGAALTVEELGLSKAARSQVDLNQAINTFMYYYYVAQIYSNLTHNKQEFQSYEENYATILGDAIAGRLMQLDTEKNARIN :  600
QIE06881.1 : YTNAAAREERETFLTQLTLVKNVLEEISKTDPNFKNLYDSCSRLLQIIDFYTDIVQKKYGGEEDCECTRVGGAALTVEELGLSKAARSQVDLNQAINTFMYYYYVAQIYSNLTHNKQEFQSYEENYATILGDAIAGRLMQLDTEKNARIN :  600
QGJ83454.1 : YTNAAAREERETFLTQLTLVKNVLEEISKTDPNFKNLYDSCSRLLQIIDFYTDIVQKKYGGEEDCECTRVGGAALTVEELGLSKAARSQVDLNQAINTFMYYYYVAQIYSNLTHNKQEFQSYEENYATILGDAIAGRLMQLDTEKNARIN :  600
AYW34063.1 : YTNAAAREERETFLTQLTLVKNVLEEISKTDPNFKNLYDSCSRLLQIIDFYTDIVQKKYGGEEDCECTRVGGAALTVEELGLSKAARSQVDLNQAINTFMYYYYVAQIYSNLTHNKQEFQSYEENYATILGDAIAGRLMQLDTEKNARIN :  600
QLF78620.1 : YTNAAAREERETFLTQLTLVKNVLEEISKTDPNFKNLYDSCSRLLQIIDFYTDIVQKKYGGEEDCECTRVGGAALTVEELGLSKAARSQVDLNQAINTFMYYYYVAQIYSNLTHNKQEFQSYEENYATILGDAIAGRLMQLDTEKNARIN :  600
QTP96400.1 : YTNAAAREERETFLTQLTLVKNVLEEISKTDPNFKNLYDSCSRLLQIIDFYTDIVQKKYGGEEDCECTRVGGAALTVEELGLSKAARSQVDLNQAINTFMYYYYVAQIYSNLTHNKQEFQSYEENYATILGDAIAGRLMQLDTEKNARIN :  600
QBH90765.1 : YTNAAAREERETFLTQLTLVKNVLEEISKTDPNFKNLYDSCSRLLQIIDFYTDIVQKKYGGEEDCECTRVGGAALTVEELGLSKAARSQVDLNQAINTFMYYYYVAQIYSNLTHNKQEFQSYEENYATILGDAIAGRLMQLDTEKNARIN :  600
QOY24398.1 : YTNAAAREERETFLTQLTLVKNVLEEISKTDPNFKNLYDSCSRLLQIIDFYTDIVQKKYGGEEDCECTRVGGAALTVEELGLSKAARSQVDLNQAINTFMYYYYVAQIYSNLTHNKQEFQSYEENYATILGDAIAGRLMQLDTEKNARIN :  600
QSG73791.1 : YTNAAAREERETFLTQLTLVKNVLEEISKTDPNFKNLYDSCSRLLQIIDFYTDIVQKKYGGEEDCECTRVGGAALTVEELGLSKAARSQVDLNQAINTFMYYYYVAQIYSNLTHNKQEFQSYEENYATILGDAIAGRLMQLDTEKNARIN :  600
QTE18752.1 : YTNAAAREERETFLTQLTLVKNVLEEISKTDPNFKNLYDSCSRLLQIIDFYTDIVQKKYGGEEDCECTRVGGAALTVEELGLSKAARSQVDLNQAINTFMYYYYVAQIYSNLTHNKQEFQSYEENYATILGDAIAGRLMQLDTEKNARIN :  600
VFV48026.1 : YTNAAAREERETFLTQLTLVKNVLEEISKTDPNFKNLYDSCSRLLQIIDFYTDIVQKKYGGEEDCECTRVGGAALTVEELGLSKAARSQVDLNQAINTFMYYYYVAQIYSNLTHNKQEFQSYEENYATILGDAIAGRLMQLDTEKNARIN :  600
QOW03225.1 : YTNAAAREERETFLTQLTLVKNVLEEISKTDPNFKNLYDSCSRLLQIIDFYTDIVQKKYGGEEDCECTRVGGAALTVEELGLSKAARSQVDLNQAINTFMYYYYVAQIYSNLTHNKQEFQSYEENYATILGDAIAGRLMQLDTEKNARIN :  600
QOW03039.1 : YTNAAAREERETFLTQLTLVKNVLEEISKTDPNFKNLYDSCSRLLQIIDFYTDIVQKKYGGEEDCECTRVGGAALTVEELGLSKAARSQVDLNQAINTFMYYYYVAQIYSNLTHNKQEFQSYEENYATILGDAIAGRLMQLDTEKNARIN :  600
VVW94185.1 : YTNAAAREERETFLTQLTLVKNVLEEISKTDPNFKNLYDSCSRLLQIIDFYTDIVQKKYGGEEDCECTRVGGAALTVEELGLSKAARSQVDLNQAINTFMYYYYVAQIYSNLTHNKQEFQSYEENYATILGDAIAGRLMQLDTEKNARIN :  600
CAD0059543 : YTNAAAREERETFLTQLTLVKNVLEEISKTDPNFKNLYDSCSRLLQIIDFYTDIVQKKYGGEEDCECTRVGGAALTVEELGLSKAARSQVDLNQAINTFMYYYYVAQIYSNLTHNKQEFQSYEENYATILGDAIAGRLMQLDTEKNARIN :  600
QXP50051.1 : YTNAAAREERETFLTQLTLVKNVLEEISKTDPNFKNLYDSCSRLLQIIDFYTDIVQKKYGGEEDCECTRVGGAALTVEELGLSKAARSQVDLNQAINTFMYYYYVAQIYSNLTHNKQEFQSYEENYATILGDAIAGRLMQLDTEKNARIN :  600
CAD7112603 : YTNAAAREERETFLTQLTLVKNVLEEISKTDPNFKNLYDSCSRLLQIIDFYTDIVQKKYGGEEDCECTRVGGAALTVEELGLSKAARSQVDLNQAINTFMYYYYVAQIYSNLTHNKQEFQSYEENYATILGDAIAGRLMQLDTEKNARIN :  600
AXZ96054.1 : YTNAAAREERETFLTQLTLVKNVLEEISKTDPNFKNLYDSCSRLLQIIDFYTDIVQKKYGGEEDCECTRVGGAALTVEELGLSKAARSQVDLNQAINTFMYYYYVAQIYSNLTHNKQEFQSYEENYATILGDAIAGRLMQLDTEKNARIN :  600
QED90535.1 : YTNAAAREERETFLTQLTLVKNVLEEISKTDPNFKNLYDSCSRLLQIIDFYTDIVQKKYGGEEDCECTRVGGAALTVEELGLSKAARSQVDLNQAINTFMYYYYVAQIYSNLTHNKQEFQSYEENYATILGDAIAGRLMQLDTEKNARIN :  600
VVW94190.1 : YTNAAAREERETFLTQLTLVKNVLEEISKTDPNFKNLYDSCSRLLQIIDFYTDIVQKKYGGEEDCECTRVGGAALTVEELGLSKAARSQVDLNQAINTFMYYYYVAQIYSNLTHNKQEFQSYEENYATILGDAIAGRLMQLDTEKNARIN :  600
QEY87898.1 : YTNAAAREERETFLTQLTLVKNVLEEISKTDPNFKNLYDSCSRLLQIIDFYTDIVQKKYGGEEDCECTRVGGAALTVEELGLSKAARSQVDLNQAINTFMYYYYVAQIYSNLTHNKQEFQSYEENYATILGDAIAGRLMQLDTEKNARIN :  600
QGV56805.1 : YTNAAAREERETFLTQLTLVKNVLEEISKTDPNFKNLYDSCSRLLQIIDFYTDIVQKKYGGEEDCECTRVGGAALTVEELGLSKAARSQVDLNQAINTFMYYYYVAQIYSNLTHNKQEFQSYEENYATILGDAIAGRLMQLDTEKNARIN :  600
QPB67618.1 : YTNAAAREERETFLTQLTLVKNVLEEISKTDPNFKNLYDSCSRLLQIIDFYTDIVQKKYGGEEDCECTRVGGAALTVEELGLSKAARSQVDLNQAINTFMYYYYVAQIYSNLTHNKQEFQSYEENYATILGDAIAGRLMQLDTEKNARIN :  600
QUQ60377.1 : YTNAAAREERETFLTQLTLVKNVLEEISKTDPNFKNLYDSCSRLLQIIDFYTDIVQKKYGGEEDCECTRVGGAALTVEELGLSKAARSQVDLNQAINTFMYYYYVAQIYSNLTHNKQEFQSYEENYATILGDAIAGRLMQLDTEKNARIN :  600
QUQ60197.1 : YTNAAAREERETFLTQLTLVKNVLEEISKTDPNFKNLYDSCSRLLQIIDFYTDIVQKKYGGEEDCECTRVGGAALTVEELGLSKAARSQVDLNQAINTFMYYYYVAQIYSNLTHNKQEFQSYEENYATILGDAIAGRLMQLDTEKNARIN :  600
SPS73515.1 : YTNAAAREERETFLTQLTLVKNVLEEISKTDPNFKNLYDSCSRLLQIIDFYTDIVQKKYGGEEDCECTRVGGAALTVEELGLSKAARSQVDLNQAINTFMYYYYVAQIYSNLTHNKQEFQSYEENYATILGDAIAGRLMQLDTEKNARIN :  600
QDL88123.1 : YTNAAAREERETFLTQLTLVKNVLEEISKTDPNFKNLYDSCSRLLQIIDFYTDIVQKKYGGEEDCECTRVGGAALTVEELGLSKAARSQVDLNQAINTFMYYYYVAQIYSNLTHNKQEFQSYEENYATILGDAIAGRLMQLDTEKNARIN :  600
QIA61506.1 : YTNAAAREERETFLTQLTLVKNVLEEISKTDPNFKNLYDSCSRLLQIIDFYTDIVQKKYGGEEDCECTRVGGAALTVEELGLSKAARSQVDLNQAINTFMYYYYVAQIYSNLTHNKQEFQSYEENYATILGDAIAGRLMQLDTEKNARIN :  600
QTZ19752.1 : YTNAAAREERETFLTQLTLVKNVLEEISKTDPNFKNLYDSCSRLLQIIDFYTDIVQKKYGGEEDCECTRVGGAALTVEELGLSKAARSQVDLNQAINTFMYYYYVAQIYSNLTHNKQEFQSYEENYATILGDAIAGRLMQLDTEKNARIN :  600
QIM07715.1 : YTNAAAREERETFLTQFMLVKNVLEEQSKIDPNFKNLYDSCSRLLQIIDFYTDIVQKKYGGGEDCECTRVGGAALTVEELGLSKAARSQVDLNQAINTFMYYYYVAQIYSNLTHNKQEFQSYEENYATILGDAIAGRLMQLDTEKNARIN :  600
QIM07948.1 : YTNAAAREERETFLTQFMLVKNVLEEQSKIDPNFKNLYDSCSRLLQIIDFYTDIVQKKYGGGEDCECTRVGGAALTVEELGLSKAARSQVDLNQAINTFMYYYYVAQIYSNLTHNKQEFQSYEENYATILGDAIAGRLMQLDTEKNARIN :  600
QIM08183.1 : YTNAAAREERETFLTQFMLVKNVLEEQSKIDPNFKNLYDSCSRLLQIIDFYTDIVQKKYGGGEDCECTRVGGAALTVEELGLSKAARSQVDLNQAINTFMYYYYVAQIYSNLTHNKQEFQSYEENYATILGDAIAGRLMQLDTEKNARIN :  600
YP_0097024 : YTNAAAREERETFLTQFMLVKNVLEEQSKIDPNFKNLYDSCSRLLQIIDFYTDIVQKKYGGGEDCECTRVGGAALTVEELGLSKAARSQVDLNQAINTFMYYYYVAQIYSNLTHNKQEFQSYEENYATILGDAIAGRLMQLDTEKNARIN :  600
YP_0097038 : YTNAAAREERETFLTQFMLVKNVLEEQSKIDPNFKNLYDSCSRLLQIIDFYTDIVQKKYGGGEDCECTRVGGAALTVEELGLSKAARSQVDLNQAINTFMYYYYVAQIYSNLTHNKQEFQSYEENYATILGDAIAGRLMQLDTEKNARIN :  600
YP_0097023 : YTNAAAREERETFLTQFMLVKNVLEEQSKIDPNFKNLYDSCSRLLQIIDFYTDIVQKKYGGGEDCECTRVGGAALTVEELGLSKAARSQVDLNQAINTFMYYYYVAQIYSNLTHNKQEFQSYEENYATILGDAIAGRLMQLDTEKNARIN :  600
```

```
YP_0097026 : YTNAAAREERETFLTQFMLVKNVLEEQSKIDPNFKNLYDSCSRLLQIIDFYTDIVQKKYGGGEDCECTRVGGAALTVEELGLSKAARSQVDLNQAINTFMYYYYVAQIYSNLTHNKQEFQSYEENYATILGDAIAGRLMQLDTEKNARIN :  600
YP_0097035 : YTNAAAREERETFLTQFMLVKNVLEEQSKIDPNFKNLYDSCSRLLQIIDFYTDIVQKKYGGGEDCECTRVGGAALTVEELGLSKAARSQVDLNQAINTFMYYYYVAQIYSNLTHNKQEFQSYEENYATILGDAIAGRLMQLDTEKNARIN :  600
QZK26792.1 : YTNAAAREERETFLTQLTLVKNVLEEISKTDPNFKNLYDSCSRLLQIIDFYTDIVQKKYGGGEDCECTRVGGAALTVEELGLSKAARSQVDLNQAINTFMYYYYVAQIYSNLTHNKQEFQSYEENYATILGDAIAGRLMQLDTEKNARIN :  600
YP_0097036 : YTNAAAREERETFLTQFMLVKNVLEEQSKIDPNFKNLYDSCSRLLQIIDFYTDIVQKKYGGGEDCECTRVGGAALTVEELGLSKAARSQVDLNQAINTFMYYYYVAQIYSNLTHNKQEFQSYEENYATILGDAIAGRLMQLDTEKNARIN :  600
QID21266.1 : YTNAAAREERETFLTQFMLVKNVLEEQSKIDPNFKNLYDSCSRLLQIIDFYTDIVQKKYGGGEDCECTRVGGAALTVEELGLSKAARSQVDLNQAINTFMYYYYVAQIYSNLTHNKQEFQSYEENYATILGDAIAGRLMQLDTEKNARIN :  600
P0CA04.1   : YTNAAAREERETFLTQFMLVKNVLEEQSKIDPNFKNLYDSCSRLLQIIDFYTDIVQKKYGGGEDCECTRVGGAALTVEELGLSKAARSQVDLNQAINTFMYYYYVAQIYSNLTHNKQEFQSYEENYATILGDAIAGRLMQLDTEKNARIN :  600
P0CA02.1   : YTNAAAREERETFLTQLTLVKNVLEEISKTDPNFKNLYDSCFRLLQIIDFYTDIVQKKYGGGEDCECTRVGGAALTVEELGLSKAARSQVDLNQAINTFMYYYYVAQIYSNLTHNKQEFQSYEENYATILGDAIAGRLMQLDTEKNARIN :  600
YP_0097029 : YNNAAAREERETFLIQLTLVKNVLEELAKTDPNFKNLYDSCFRLLQIIDFYTDIVQKKYGGGEDCECTKVGGAALTVEELGLSKAARSQVDLNQAINTFMYYYYVAQIYSNLTHNKQEFQSYEENYATILGDAIAGRLMQLDTEKNARIN :  600
AXB50010.1 : YNNAAAREERETFLIQLTLVKNVLEELAKTDPNFKNLYDSCFRLLQIIDFYTDIVQKKYGGGEDCECTKVGGAALTVEELGLSKAARSQVDLNQAINTFMYYYYVAQIYSNLTHNKQEFQSYEENYATILGDAIAGRLMQLDTEKNARIN :  600
AXB49666.1 : YNNAAAREERETFLIQLTLVKNVLEELAKTDPNFKNLYDSCFRLLQIIDFYTDIVQKKYGGGEDCECTKVGGAALTVEELGLSKAARSQVDLNQAINTFMYYYYVAQIYSNLTHNKQEFQSYEENYATILGDAIAGRLMQLDTEKNARIN :  600
CAD7112308 : YNNAAAREERETFLIQLTLVKNVLEELAKTDPNFKNLYDSCFRLLQIIDFYTDIVQKKYGGGEDCECTKVGGAALTVEELGLSKAARSQVDLNQAINTFMYYYYVAQIYSNLTHNKQEFQSYEENYATILGDAIAGRLMQLDTEKNARIN :  600
QRY19118.1 : YNNAAAREERETFLIQLTLVKNVLEELAKTDPNFKNLYDSCFRLLQIIDFYTDIVQKKYGGGEDCECTKVGGAALTVEELGLSKAARSQVDLNQAINTFMYYYYVAQIYSNLTHNKQEFQSYEENYATILGDAIAGRLMQLDTEKNARIN :  600
P0CA01.1   : YNNAAAREERETFLIQLTLVKNVLEELAKTDPNFKNLYDSCFRLLQIIDFYTDIVQKKYGGGEDCECTKVGGAALTVEELGLSKAARSQVDLNQAINTFMYYYYVAQIYSNLTHNKQEFQSYEENYATILGDAIAGRLMQLDTEKNARIN :  600
YP_0097028 : YNNAAAREERETFLIQLTLVKNVLEELAKTDPNFKNLYDSCFRLLQIIDFYTDIVQKKYGGGEDCECTKVGGAALTVEELGLSKAARSQVDLNQAINTFMYYYYVAQIYSNLTHNKQEFQSYEENYATILGDAIAGRLMQLDTEKNARIN :  600
P0CA03.1   : YTNAAAREERETFLTQFMLVKNVLEEQSKTDPNFKNLYDSCSRLLQIIDFYTDIVQKKYGGEEDCECTRVGGAALTVEELGLSKAARSQVDLNQAINTFMYYYYVAQIYSNLTHNKQEFQSYEENYATILGDAIAGRLMQLDTEKNARIN :  600

                      *        620         *        640         *        660         *        680         *        700         *        720         *        740         *
YP_0099272 : SPAVDLARGHVGPNPGGAQEVDWKATVSAIELEYDVKRRFYRALEGLDLYLKNITKTFVNNIDSIQTVQQMLDGVRIIGRWFTETTGDTLAQVFESFPTSTGNDSNVFTDNAPAGHYYEKVAAEIQQGRSVGTLRPVRASQAKNIRDLIG :  750
AZP54139.1 : SPAVDLARGHVGPNPGGAQEVDWKATVSAIELEYDVKRRFYRALEGLDLYLKNITKTFVNNIDSIQTVQQMLDGVRIIGRWFTETTGDTLAQVFESFPTSTGNDSNVFTDNAPAGHYYEKVAAEIQQGRSVGTLRPVRASQAKNIRDLIG :  750
QIE06881.1 : SPAVDLARGHVGPNPGGAQEVDWKATVSAIELEYDVKRRFYRALEGLDLYLKNITKTFVNNIDSIQTVQQMLDGVRIIGRWFTETTGDTLAQVFESFPTSTGNDSNVFTDNAPAGHYYEKVAAEIQQGRSVGTLRPVRASQAKNIRDLIG :  750
QGJ83454.1 : SPAVDLARGHVGPNPGGAQEVDWKATVSAIELEYDVKRRFYRALEGLDLYLKNITKTFVNNIDSIQTVQQMLDGVRIIGRWFTETTGDTLAQVFESFPTSTGNDSNVFTDNAPAGHYYEKVAAEIQQGRSVGTLRPVRASQAKNIRDLIG :  750
AYW34063.1 : SPAVDLARGHVGPNPGGAQEVDWKATVSAIELEYDVKRRFYRALEGLDLYLKNITKTFVNNIDSIQTVQQMLDGVRIIGRWFTETTGDTLAQVFESFPTSTGNDSNVFTDNAPAGHYYEKVAAEIQQGRSVGTLRPVRASQAKNIRDLIG :  750
QLF78620.1 : SPAVDLARGHVGPNPGGAQEVDWKATVSAIELEYDVKRRFYRALEGLDLYLKNITKTFVNNIDSIQTVQQMLDGVRIIGRWFTETTGDTLAQVFESFPTSTGNDSNVFTDNAPAGHYYEKVAAEIQQGRSVGTLRPVRASQAKNIRDLIG :  750
QTP96400.1 : SPAVDLARGHVGPNPGGAQEVDWKATVSAIELEYDVKRRFYRALEGLDLYLKNITKTFVNNIDSIQTVQQMLDGVRIIGRWFTETTGDTLAQVFESFPTSTGNDSNVFTDNAPAGHYYEKVAAEIQQGRSVGTLRPVRASQAKNIRDLIG :  750
QBH90765.1 : SPAVDLARGHVGPNPGGAQEVDWKATVSAIELEYDVKRRFYRALEGLDLYLKNITKTFVNNIDSIQTVQQMLDGVRIIGRWFTETTGDTLAQVFESFPTSTGNDSNVFTDNAPAGHYYEKVAAEIQQGRSVGTLRPVRASQAKNIRDLIG :  750
QOY24398.1 : SPAVDLARGHVGPNPGGAQEVDWKATVSAIELEYDVKRRFYRALEGLDLYLKNITKTFVNNIDSIQTVQQMLDGVRIIGRWFTETTGDTLAQVFESFPTSTGNDSNVFTDNAPAGHYYEKVAAEIQQGRSVGTLRPVRASQAKNIRDLIG :  750
QSG73791.1 : SPAVDLARGHVGPNPGGAQEVDWKATVSAIELEYDVKRRFYRALEGLDLYLKNITKTFVNNIDSIQTVQQMLDGVRIIGRWFTETTGDTLAQVFESFPTSTGNDSNVFTDNAPAGHYYEKVAAEIQQGRSVGTLRPVRASQAKNIRDLIG :  750
QTE18752.1 : SPAVDLARGHVGPNPGGAQEVDWKATVSAIELEYDVKRRFYRALEGLDLYLKNITKTFVNNIDSIQTVQQMLDGVRIIGRWFTETTGDTLAQVFESFPTSTGNDSNVFTDNAPAGHYYEKVAAEIQQGRSVGTLRPVRASQAKNIRDLIG :  750
VFV48026.1 : SPAVDLARGHVGPNPGGAQEVDWKATVSAIELEYDVKRRFYRALEGLDLYLKNITKTFVNNIDSIQTVQQMLDGVRIIGRWFTETTGDTLAQVFESFPTSTGNDSNVFTDNAPAGHYYEKVAAEIQQGRSVGTLRPVRASQAKNIRDLIG :  750
QOW03225.1 : SPAVDLARGHVGPNPGGAQEVDWKATVSAIELEYDVKRRFYRALEGLDLYLKNITKTFVNNIDSIQTVQQMLDGVRIIGRWFTETTGDTLAQVFESFPTSTGNDSNVFTDNAPAGHYYEKVAAEIQQGRSVGTLRPVRASQAKNIRDLIG :  750
QOW03039.1 : SPAVDLARGHVGPNPGGAQEVDWKATVSAIELEYDVKRRFYRALEGLDLYLKNITKTFVNNIDSIQTVQQMLDGVRIIGRWFTETTGDTLAQVFESFPTSTGNDSNVFTDNAPAGHYYEKVAAEIQQGRSVGTLRPVRASQAKNIRDLIG :  750
VVW94185.1 : SPAVDLARGHVGPNPGGAQEVDWKATVSAIELEYDVKRRFYRALEGLDLYLKNITKTFVNNIDSIQTVQQMLDGVRIIGRWFTETTGDTLAQVFESFPTSTGNDSNVFTDNAPAGHYYEKVAAEIQQGRSVGTLRPVRASQAKNIRDLIG :  750
CAD0059543 : SPAVDLARGHVGPNPGGAQEVDWKATVSAIELEYDVKRRFYRALEGLDLYLKNITKTFVNNIDSIQTVQQMLDGVRIIGRWFTETTGDTLAQVFESFPTSTGNDSNVFTDNAPAGHYYEKVAAEIQQGRSVGTLRPVRASQAKNIRDLIG :  750
QXP50051.1 : SPAVDLARGHVGPNPGGAQEVDWKATVSAIELEYDVKRRFYRALEGLDLYLKNITKTFVNNIDSIQTVQQMLDGVRIIGRWFTETTGDTLAQVFESFPTSTGNDSNVFTDNAPAGHYYEKVAAEIQQGRSVGTLRPVRASQAKNIRDLIG :  750
CAD7112603 : SPAVDLARGHVGPNPGGAQEVDWKATVSAIELEYDVKRRFYRALEGLDLYLKNITKTFVNNIDSIQTVQQMLDGVRIIGRWFTETTGDTLAQVFESFPTSTGNDSNVFTDNAPAGHYYEKVAAEIQQGRSVGTLRPVRASQAKNIRDLIG :  750
AXZ96054.1 : SPAVDLARGHVGPNPGGAQEVDWKATVSAIELEYDVKRRFYRALEGLDLYLKNITKTFVNNIDSIQTVQQMLDGVRIIGRWFTETTGDTLAQVFESFPTSTGNDSNVFTDNAPAGHYYEKVAAEIQQGRSVGTLRPVRASQAKNIRDLIG :  750
QED90535.1 : SPAVDLARGHVGPNPGGAQEVDWKATVSAIELEYDVKRRFYRALEGLDLYLKNITKTFVNNIDSIQTVQQMLDGVRIIGRWFTETTGDTLAQVFESFPTSTGNDSNVFTDNAPAGHYYEKVAAEIQQGRSVGTLRPVRASQAKNIRDLIG :  750
VVW94190.1 : SPAVDLARGHVGPNPGGAQEVDWKATVSAIELEYDVKRRFYRALEGLDLYLKNITKTFVNNIDSIQTVQQMLDGVRIIGRWFTETTGDTLAQVFESFPTSTGNDSNVFTDNAPAGHYYEKVAAEIQQGRSVGTLRPVRASQAKNIRDLIG :  750
QEY87898.1 : SPAVDLARGHVGPNPGGAQEVDWKATVSAIELEYDVKRRFYRALEGLDLYLKNITKTFVNNIDSIQTVQQMLDGVRIIGRWFTETTGDTLAQVFESFPTSTGNDSNVFTDNAPAGHYYEKVAAEIQQGRSVGTLRPVRASQAKNIRDLIG :  750
QGV56805.1 : SPAVDLARGHVGPNPGGAQEVDWKATVSAIELEYDVKRRFYRALEGLDLYLKNITKTFVNNIDSIQTVQQMLDGVRIIGRWFTETTGDTLAQVFESFPTSTGNDSNVFTDNAPAGHYYEKVAAEIQQGRSVGTLRPVRASQAKNIRDLIG :  750
QPB67618.1 : SPAVDLARGHVGPNPGGAQEVDWKATVSAIELEYDVKRRFYRALEGLDLYLKNITKTFVNNIDSIQTVQQMLDGVRIIGRWFTETTGDTLAQVFESFPTSTGNDSNVFTDNAPAGHYYEKVAAEIQQGRSVGTLRPVRASQAKNIRDLIG :  750
QUQ60377.1 : SPAVDLARGHVGPNPGGAQEVDWKATVSAIELEYDVKRRFYRALEGLDLYLKNITKTFVNNIDSIQTVQQMLDGVRIIGRWFTETTGDTLAQVFESFPTSTGNDSNVFTDNAPAGHYYEKVAAEIQQGRSVGTLRPVRASQAKNIRDLIG :  750
QUQ60197.1 : SPAVDLARGHVGPNPGGAQEVDWKATVSAIELEYDVKRRFYRALEGLDLYLKNITKTFVNNIDSIQTVQQMLDGVRIIGRWFTETTGDTLAQVFESFPTSTGNDSNVFTDNAPAGHYYEKVAAEIQQGRSVGTLRPVRASQAKNIRDLIG :  750
SPS73515.1 : SPAVDLARGHVGPNPGGAQEVDWKATVSAIELEYDVKRRFYRALEGLDLYLKNITKTFVNNIDSIQTVQQMLDGVRIIGRWFTETTGDTLAQVFESFPTSTGNDSNVFTDNAPAGHYYEKVAAEIQQGRSVGTLRPVRASQAKNIRDLIG :  750
QDL88123.1 : SPAVDLARGHVGPNPGGAQEVDWKATVSAIELEYDVKRRFYRALEGLDLYLKNITKTFVNNIDSIQTVQQMLDGVRIIGRWFTETTGDTLAQVFESFPTSTGNDSNVFTDNAPAGHYYEKVAAEIQQGRSVGTLRPVRASQAKNIRDLIG :  750
QIA61506.1 : SPAVDLARGHVGPNPGGAQEVDWKATVSAIELEYDVKRRFYRALEGLDLYLKNITKTFVNNIDSIQTVQQMLDGVRIIGRWFTETTGDTLAQVFESFPTSTGNDSNVFTDNAPAGHYYEKVAAEIQQGRSVGTLRPVRASQAKNIRDLIG :  750
QTZ19752.1 : SPAVDLARGHVGPNPGGAQEVDWKATVSAIELEYDVKRRFYRALEGLDLYLKNITKTFVNNIDSIQTVQQMLDGVRIIGRWFTETTGDTLAQVFESFPTSTGNDSNVFTDNAPAGHYYEKVAAEIQQGRSVGTLRPVRASQAKNIRDLIG :  750
QIM07715.1 : SPAVDLARGHVGPNPGGAQEADWKAAVSAIELEYDVKRRFYRALEGLDLYLKNITKTFVNNIDSIQTVQQMLDGVRIIGRWFTEATGDTLAQVFESFPTSAGNDSNVFTDNAPAGHYYEKVAAEIQQGRSVGTLRPVRASQAKNIRDLIG :  750
QIM07948.1 : SPAVDLARGHVGPNPGGAQEADWKAAVSAIELEYDVKRRFYRALEGLDLYLKNITKTFVNNIDSIQTVQQMLDGVRIIGRWFTEATGDTLAQVFESFPTSAGNDSNVFTDNAPAGHYYEKVAAEIQQGRSVGTLRPVRASQAKNIRDLIG :  750
QIM08183.1 : SPAVDLARGHVGPNPGGAQEADWKAAVSAIELEYDVKRRFYRALEGLDLYLKNITKTFVNNIDSIQTVQQMLDGVRIIGRWFTEATGDTLAQVFESFPTSAGNDSNVFTDNAPAGHYYEKVAAEIQQGRSVGTLRPVRASQAKNIRDLIG :  750
YP_0097024 : SPAVDLARGHVGPNPGGAQEADWKAAVSAIELEYDVKRRFYRALEGLDLYLKNITKTFVNNIDSIQTVQQMLDGVRIIGRWFTEATGDTLAQVFESFPTSAGNDSNVFTDNAPAGHYYEKVAAEIQQGRSVGTLRPVRASQAKNIRDLIG :  750
YP_0097038 : SPAVDLARGHVGPNPGGAQEADWKAAVSAIELEYDVKRRFYRALEGLDLYLKNITKTFVNNIDSIQTVQQMLDGVRIIGRWFTEATGDTLAQVFESFPTSAGNDSNVFTDNAPAGHYYEKVAAEIQQGRSVGTLRPVRASQAKNIRDLIG :  750
YP_0097023 : SPAVDLARGHVGPNPGGAQEADWKAAVSAIELEYDVKRRFYRALEGLDLYLKNITKTFVNNIDSIQTVQQMLDGVRIIGRWFTEATGDTLAQVFESFPTSAGNDSNVFTDNAPAGHYYEKVAAEIQQGRSVGTLRPVRASQAKNIRDLIG :  750
YP_0097026 : SPAVDLARGHVGPNPGGAQEADWKAAVSAIELEYDVKRRFYRALEGLDLYLKNITKTFVNNIDSIQTVQQMLDGVRIIGRWFTEATGDTLAQVFESFPTSAGNDSNVFTDNAPAGHYYEKVAAEIQQGRSVGTLRPVRASQAKNIRDLIG :  750
YP_0097035 : SPAVDLARGHVGPNPGGAQEADWKAAVSAIELEYDVKRRFYRALEGLDLYLKNITKTFVNNIDSIQTVQQMLDGVRIIGRWFTEATGDTLAQVFESFPTSAGNDSNVFTDNAPAGHYYEKVAAEIQQGRSVGTLRPVRASQAKNIRDLIG :  750
QZK26792.1 : SPAVDLARGHVGPNPGGAQEADWKAAVSAIELEYDVKRRFYRALEGLDLYLKNITKTFVNNIDSIQTVQQMLDGVRIIGRWFTEATGDTLAQVFESFPTSAGNDSNVFTDNAPAGHYYEKVAAEIQQGRSVGTLRPVRASQAKNIRDLIG :  750
YP_0097036 : SPAVDLARGHVGPNPGGAQEADWKAAVSAIELEYDVKRRFYRALEGLDLYLKNITKTFVNNIDSIQTVQQMLDGVRIIGRWFTEATGDTLAQVFESFPTSAGNDSNVFTDNAPAGHYYEKVAAEIQQGRSVGTLRPVRASQAKNIRDLIG :  750
QID21266.1 : SPAVDLARGHVGPNPGGAQEVDWKAAVSAIELEYDVKRRFYRALEGLDLYLKNITKTFVNNIDSIQTVQQMLDGVRIIGRWFTEATGDTLAQVFESFPTSAGNDSNVFTDNAPAGHYYEKVAAEIQQGRSVGTLRPVRASQAKNIRDLIG :  750
P0CA04.1   : SPAVDLARGHVGPNPGGAQEEDWKATVSAIELEYDVKRRFYRALEGLDLYLKNITKTFVNNIDSIQTVQQMLDGVRIIGRWFTEATGDTLAQVFESFPTSAGNDSNVFTDNAPAGHYYEKVAAEIQQGRSVGTLRPVRASQAKNIRDLIG :  750
P0CA02.1   : SPAVDLARGHVGPNPGGAQEVDWKAAVSAIELEYDVKRRFYRALEGLDLYLKNITKTFVNNIDSIQTVQQMLDGVRIIGRWFTEATGDTLAQVFESFPTSAGNDSNVFTDNAPAGHYYEKVAAEIQQGRSVGTLRPVRASQAKNIRDLIG :  750
YP_0097029 : SPAVDLARGHVGPNPGGAQEVDWKATISAIELEYDVKRRFYRALEGLDLYLKNITKTFVNNIDSIQTVQQMLDGVRIIGRWFTEATGDTLAQVFESFPTSAGNDSNVFTDNAPAGHYYEKVAAEIQQGRGVGTLRPVRASQAKNIRDLIG :  750
AXB50010.1 : SPAVDLARGHVGPNPGGAQEVDWKATISAIELEYDVKRRFYRALEGLDLYLKNITKTFVNNIDSIQTVQQMLDGVRIIGRWFTEATGDTLAQVFESFPTSAGNDSNVFTDNAPAGHYYEKVAAEIQQGRGVGTLRPVRASQAKNIRDLIG :  750
AXB49666.1 : SPAVDLARGHVGPNPGGAQEVDWKATISAIELEYDVKRRFYRALEGLDLYLKNITKTFVNNIDSIQTVQQMLDGVRIIGRWFTEATGDTLAQVFESFPTSAGNDSNVFTDNAPAGHYYEKVAAEIQQGRGVGTLRPVRASQAKNIRDLIG :  750
CAD7112308 : SPAVDLARGHVGPNPGGAQEVDWKATISAIELEYDVKRRFYRALEGLDLYLKNITKTFVNNIDSIQTVQQMLDGVRIIGRWFTEATGDTLAQVFESFPTSAGNDSNVFTDNAPAGHYYEKVAAEIQQGRGVGTLRPVRASQAKNIRDLIG :  750
QRY19118.1 : SPAVDLARGHVGPNPGGAQEIDWKATISAIELEYDVKRRFYRALEGLDLYLKNITKTFVNNIDSIQTVQQMLDGVRIIGRWFTEATGDTLAQVFESFPTSAGNDSNVFTDNAPAGHYYEKVAAEIQQGRGVGTLRPVRASQAKNIRDLIG :  750
P0CA01.1   : SPAVDLARGHVGPNPGGAQEVDWKATISAIELEYDVKRRFYRALEGLDLYLKNITKTFVNNIDSIQTVQQMLDGVRIIGRWFTEATGDTLAQVFESFPTSAGNDSNVFTDNAPAGHYYEKVAAEIQQGRGVGTLRPVRASQAKNIRDLIG :  750
YP_0097028 : SPAVDLARGHVGPNPGGAQEVDWKATISAIELEYDVKRRFYRALEGLDLYLKNITKTFVNNIDSIQTVQQMLDGVRIIGRWFTEATGDTLAQVFESFPTSAGNDSNVFTDNAPAGHYYEKVAAEIQQGRGVGTLRPVRASQAKNIRDLIG :  750
```

```
P0CA03.1   : SPAVDLARGHVGPNPGGAQEVDWKAAVSAIELEYDVKRRFYRALEGLDLYLKNITKTFVNNIDSIQTVQQMLDGVRIIGRWFTEATGDTLAQVFESFPTSAGNDSNVFTDNAPAGHYYEKVAAEIQQGRSVGTLRPVRASQAKNIRDLIG :  750

                     760         *       780         *       800         *       820         *       840         *       860         *       880         *       900
YP_0099272 : RSLSNFQALKNIINAFARIGDMLGGEELRQMVPMSPLQIYKTLLEYLQHSALSVGLKNLNQSEIGGQRMALAQTAEEAAQRVYLSTVRVNDALSTRWETEDVFFTFMLKSMAAKIFIVLGIYDMFERPEPVYKLIPTRMILGGADELEPE :  900
AZP54139.1 : RSLSNFQALKNIINAFARIGDMLGGEELRQMVPMSPLQIYKTLLEYLQHSALSVGLKNLNQSEIGGQRMALAQTAEEAAQRVYLSTVRVNDALSTRWETEDVFFTFMLKSMAAKIFIVLGIYDMFERPEPVYKLIPTRMILGGADELEPE :  900
QIE06881.1 : RSLSNFQALKNIINAFARIGDMLGGEELRQMVPMSPLQIYKTLLEYLQHSALSVGLKNLNQSEIGGQRMALAQTAEEAAQRVYLSTVRVNDALSTRWETEDVFFTFMLKSMAAKIFIVLGIYDMFERPEPVYKLIPTRMILGGADELEPE :  900
QGJ83454.1 : RSLSNFQALKNIINAFARIGDMLGGEELRQMVPMSPLQIYKTLLEYLQHSALSVGLKNLNQSEIGGQRMALAQTAEEAAQRVYLSTVRVNDALSTRWETEDVFFTFMLKSMAAKIFIVLGIYDMFERPEPVYKLIPTRMILGGADELEPE :  900
AYW34063.1 : RSLSNFQALKNIINAFARIGDMLGGEELRQMVPMSPLQIYKTLLEYLQHSALSVGLKNLNQSEIGGQRMALAQTAEEAAQRVYLSTVRVNDALSTRWETEDVFFTFMLKSMAAKIFIVLGIYDMFERPEPVYKLIPTRMILGGADELEPE :  900
QLF78620.1 : RSLSNFQALKNIINAFARIGDMLGGEELRQMVPMSPLQIYKTLLEYLQHSALSVGLKNLNQSEIGGQRMALAQTAEEAAQRVYLSTVRVNDALSTRWETEDVFFTFMLKSMAAKIFIVLGIYDMFERPEPVYKLIPTRMILGGADELEPE :  900
QTP96400.1 : RSLSNFQALKNIINAFARIGDMLGGEELRQMVPMSPLQIYKTLLEYLQHSALSVGLKNLNQSEIGGQRMALAQTAEEAAQRVYLSTVRVNDALSTRWETEDVFFTFMLKSMAAKIFIVLGIYDMFERPEPVYKLIPTRMILGGADELEPE :  900
QBH90765.1 : RSLSNFQALKNIINAFARIGDMLGGEELRQMVPMSPLQIYKTLLEYLQHSALSVGLKNLNQSEIGGQRMALAQTAEEAAQRVYLSTVRVNDALSTRWETEDVFFTFMLKSMAAKIFIVLGIYDMFERPEPVYKLIPTRMILGGADELEPE :  900
QOY24398.1 : RSLSNFQALKNIINAFARIGDMLGGEELRQMVPMSPLQIYKTLLEYLQHSALSVGLKNLNQSEIGGQRMALAQTAEEAAQRVYLSTVRVNDALSTRWETEDVFFTFMLKSMAAKIFIVLGIYDMFERPEPVYKLIPTRMILGGADELEPE :  900
QSG73791.1 : RSLSNFQALKNIINAFARIGDMLGGEELRQMVPMSPLQIYKTLLEYLQHSALSVGLKNLNQSEIGGQRMALAQTAEEAAQRVYLSTVRVNDALSTRWETEDVFFTFMLKSMAAKIFIVLGIYDMFERPEPVYKLIPTRMILGGADELEPE :  900
QTE18752.1 : RSLSNFQALKNIINAFARIGDMLGGEELRQMVPMSPLQIYKTLLEYLQHSALSVGLKNLNQSEIGGQRMALAQTAEEAAQRVYLSTVRVNDALSTRWETEDVFFTFMLKSMAAKIFIVLGIYDMFERPEPVYKLIPTRMILGGADELEPE :  900
VFV48026.1 : RSLSNFQALKNIINAFARIGDMLGGEELRQMVPMSPLQIYKTLLEYLQHSALSVGLKNLNQSEIGGQRMALAQTAEEAAQRVYLSTVRVNDALSTRWETEDVFFTFMLKSMAAKIFIVLGIYDMFERPEPVYKLIPTRMILGGADELEPE :  900
QOW03225.1 : RSLSNFQALKNIINAFARIGDMLGGEELRQMVPMSPLQIYKTLLEYLQHSALSVGLKNLNQSEIGGQRMALAQTAEEAAQRVYLSTVRVNDALSTRWETEDVFFTFMLKSMAAKIFIVLGIYDMFERPEPVYKLIPTRMILGGADELEPE :  900
QOW03039.1 : RSLSNFQALKNIINAFARIGDMLGGEELRQMVPMSPLQIYKTLLEYLQHSALSVGLKNLNQSEIGGQRMALAQTAEEAAQRVYLSTVRVNDALSTRWETEDVFFTFMLKSMAAKIFIVLGIYDMFERPEPVYKLIPTRMILGGADELEPE :  900
VVW94185.1 : RSLSNFQALKNIINAFARIGDMLGGEELRQMVPMSPLQIYKTLLEYLQHSALSVGLKNLNQSEIGGQRMALAQTAEEAAQRVYLSTVRVNDALSTRWETEDVFFTFMLKSMAAKIFIVLGIYDMFERPEPVYKLIPTRMILGGADELEPE :  900
CAD0059543 : RSLSNFQALKNIINAFARIGDMLGGEELRQMVPMSPLQIYKTLLEYLQHSALSVGLKNLNQSEIGGQRMALAQTAEEAAQRVYLSTVRVNDALSTRWETEDVFFTFMLKSMAAKIFIVLGIYDMFERPEPVYKLIPTRMILGGADELEPE :  900
QXP50051.1 : RSLSNFQALKNIINAFARIGDMLGGEELRQMVPMSPLQIYKTLLEYLQHSALSVGLKNLNQSEIGGQRMALAQTAEEAAQRVYLSTVRVNDALSTRWETEDVFFTFMLKSMAAKIFIVLGIYDMFERPEPVYKLIPTRMILGGADELEPE :  900
CAD7112603 : RSLSNFQALKNIINAFARIGDMLGGEELRQMVPMSPLQIYKTLLEYLQHSALSVGLKNLNQSEIGGQRMALAQTAEEAAQRVYLSTVRVNDALSTRWETEDVFFTFMLKSMAAKIFIVLGIYDMFERPEPVYKLIPTRMILGGADELEPE :  900
AXZ96054.1 : RSLSNFQALKNIINAFARIGDMLGGEELRQMVPMSPLQIYKTLLEYLQHSALSVGLKNLNQSEIGGQRMALAQTAEEAAQRVYLSTVRVNDALSTRWETEDVFFTFMLKSMAAKIFIVLGIYDMFERPEPVYKLIPTRMILGGADELEPE :  900
QED90535.1 : RSLSNFQALKNIINAFARIGDMLGGEELRQMVPMSPLQIYKTLLEYLQHSALSVGLKNLNQSEIGGQRMALAQTAEEAAQRVYLSTVRVNDALSTRWETEDVFFTFMLKSMAAKIFIVLGIYDMFERPEPVYKLIPTRMILGGADELEPE :  900
VVW94190.1 : RSLSNFQALKNIINAFARIGDMLGGEELRQMVPMSPLQIYKTLLEYLQHSALSVGLKNLNQSEIGGQRMALAQTAEEAAQRVYLSTVRVNDALSTRWETEDVFFTFMLKSMAAKIFIVLGIYDMFERPEPVYKLIPTRMILGGADELEPE :  900
QEY87898.1 : RSLSNFQALKNIINAFARIGDMLGGEELRQMVPMSPLQIYKTLLEYLQHSALSVGLKNLNQSEIGGQRMALAQTAEEAAQRVYLSTVRVNDALSTRWETEDVFFTFMLKSMAAKIFIVLGIYDMFERPEPVYKLIPTRMILGGADELEPE :  900
QGV56805.1 : RSLSNFQALKNIINAFARIGDMLGGEELRQMVPMSPLQIYKTLLEYLQHSALSVGLKNLNQSEIGGQRMALAQTAEEAAQRVYLSTVRVNDALSTRWETEDVFFTFMLKSMAAKIFIVLGIYDMFERPEPVYKLIPTRMILGGADELEPE :  900
QPB67618.1 : RSLSNFQALKNIINAFARIGDMLGGEELRQMVPMSPLQIYKTLLEYLQHSALSVGLKNLNQSEIGGQRMALAQTAEEAAQRVYLSTVRVNDALSTRWETEDVFFTFMLKSMAAKIFIVLGIYDMFERPEPVYKLIPTRMILGGADELEPE :  900
QUQ60377.1 : RSLSNFQALKNIINAFARIGDMLGGEELRQMVPMSPLQIYKTLLEYLQHSALSVGLKNLNQSEIGGQRMALAQTAEEAAQRVYLSTVRVNDALSTRWETEDVFFTFMLKSMAAKIFIVLGIYDMFERPEPVYKLIPTRMILGGADELEPE :  900
QUQ60197.1 : RSLSNFQALKNIINAFARIGDMLGGEELRQMVPMSPLQIYKTLLEYLQHSALSVGLKNLNQSEIGGQRMALAQTAEEAAQRVYLSTVRVNDALSTRWETEDVFFTFMLKSMAAKIFIVLGIYDMFERPEPVYKLIPTRMILGGADELEPE :  900
SPS73515.1 : RSLSNFQALKNIINAFARIGDMLGGEELRQMVPMSPLQIYKTLLEYLQHSALSVGLKNLNQSEIGGQRMALAQTAEEAAQRVYLSTVRVNDALSTRWETEDVFFTFMLKSMAAKIFIVLGIYDMFERPEPVYKLIPTRMILGGADELEPE :  900
QDL88123.1 : RSLSNFQALKNIINAFARIGDMLGGEELRQMVPMSPLQIYKTLLEYLQHSALSVGLKNLNQSEIGGQRMALAQTAEEAAQRVYLSTVRVNDALSTRWETEDVFFTFMLKSMAAKIFIVLGIYDMFERPEPVYKLIPTRMILGGADELEPE :  900
QIA61506.1 : RSLSNFQALKNIINAFARIGDMLGGEELRQMVPMSPLQIYKTLLEYLQHSALSVGLKNLNQSEIGGQRMALAQTAEEAAQRVYLSTVRVNDALSTRWETEDVFFTFMLKSMAAKIFIVLGIYDMFERPEPVYKLIPTRMILGGADELEPE :  900
QTZ19752.1 : RSLSNFQALKNIINAFARIGDMLGGEELRQMVPMSPLQIYKTLLEYLQHSALSVGLKNLNQSEIGGQRMALAQTAEEAAQRVYLSTVRVNDALSTRWETEDVFFTFMLKSMAAKIFIVLGIYDMFERPEPVYKLIPTRMILGGADELEPE :  900
QIM07715.1 : RSLSNFQALKNIINAFARIGDMLGGEELRQMVPMSPLQIYKTLLEYIQHSALSVGLKNLNQSEIGGQRVALARTPEEAAQRVYLSTVRVNDALSTRWETEDVFFTFMLKSMAAKIFIVLGIYDMFERPEPVYKLIPTRMILGGADELEPE :  900
QIM07948.1 : RSLSNFQALKNIINAFARIGDMLGGEELRQMVPMSPLQIYKTLLEYIQHSALSVGLKNLNQSEIGGQRVALARTPEEAAQRVYLSTVRVNDALSTRWETEDVFFTFMLKSMAAKIFIVLGIYDMFERPEPVYKLIPTRMILGGADELEPE :  900
QIM08183.1 : RSLSNFQALKNIINAFARIGDMLGGEELRQMVPMSPLQIYKTLLEYIQHSALSVGLKNLNQSEIGGQRVALARTPEEAAQRVYLSTVRVNDALSTRWETEDVFFTFMLKSMAAKIFIVLGIYDMFERPEPVYKLIPTRMILGGADELEPE :  900
YP_0097024 : RSLSNFQALKNIINAFARIGDMLGGEELRQMVPMSPLQIYKTLLEYIQHSALSVGLKNLNQSEIGGQRVALARTPEEAAQRVYLSTVRVNDALSTRWETEDVFFTFMLKSMAAKIFIVLGIYDMFERPEPVYKLIPTRMILGGADELEPE :  900
YP_0097038 : RSLSNFQALKNIINAFARIGDMLGGEELRQMVPMSPLQIYKTLLEYIQHSALSVGLKNLNQSEIGGQRVALARTPEEAAQRVYLSTVRVNDALSTRWETEDVFFTFMLKSMAAKIFIVLGIYDMFERPEPVYKLIPTRMILGGADELEPE :  900
YP_0097023 : RSLSNFQALKNIINAFARIGDMLGGEELRQMVPMSPLQIYKTLLEYIQHSALSVGLKNLNQSEIGGQRVALARTPEEAAQRVYLSTVRVNDALSTRWETEDVFFTFMLKSMAAKIFIVLGIYDMFERPEPVYKLIPTRMILGGADELEPE :  900
YP_0097026 : RSLSNFQALKNIINAFARIGDMLGGEELRQMVPMSPLQIYKTLLEYIQHSALSVGLKNLNQSEIGGQRVALARTPEEAAQRVYLSTVRVNDALSTRWETEDVFFTFMLKSMAAKIFIVLGIYDMFERPEPVYKLIPTRMILGGADELEPE :  900
YP_0097035 : RSLSNFQALKNIINAFARIGDMLGGEELRQMVPMSPLQIYKTLLEYIQHSALSVGLKNLNQSEIGGQRVALARTPEEAAQRVYLSTVRVNDALSTRWETEDVFFTFMLKSMAAKIFIVLGIYDMFERPEPVYKLIPTRMILGGADELEPE :  900
QZK26792.1 : RSLSNFQALKNIINAFARIGDMLGGEELRQTVPMSPLQIYKTLLEYLQHSALSVGLKNLNQSEIGGQRMALAQTAEEAAQRVYLSTVRVNDALSTRWETEDVFFTFMLKSMAAKIFIVLGIYDMFERPEPVYKLIPTRMILGGADELEPE :  900
YP_0097036 : RSLSNFQALKNIINAFARIGDMLGGEELRQMVPMSPLQIYKTLLEYIQHSALSVGLKNLNQSEIGGQRVALARTPEEAAQRVYLSTVRVNDALSTRWETEDVFFTFMLKSMAAKIFIVLGIYDMFERPEPVYKLIPTRMILGGADELEPE :  900
QID21266.1 : RSLSNFQALKNIINAFARIGDMLGGEELRQMVPMSPLQIYKTLLEYLQHSALSVGLKNLNQSEIGGQRVALARTPEEAAQRVYLSTVRVNDALSTRWETEDVFFTFMLKSMAAKIFIVLGIYDMFERPEPVYKLIPTRMILGGADELEPE :  900
P0CA04.1   : RSLSNFQALKNIINAFARIGDMLGGEELRQMVPMSPLQIYKTLLEYIQHSALSVGLKNLNQSEIGGQRVALARTPEEAAQRVYLSTVRVNDALSTRWETEDVFFTFMLKSMAAKIFIVLGIYDMFERPEPVYKLIPTRMILGGADELEPE :  900
P0CA02.1   : RSLSNFQALKNIINAFARIGDMLGGEELRQMVPMSPLQIYKTLLEYLQHSALSVGLKNLNQSEIGGQRMALAQTAEEAAQRVYLSTVRVNDALSSRWETEDMFFTFMLKSMAAKIFIVLGIYDMFERPEPVYKLIPTRMILGGADELEPE :  900
YP_0097029 : RSLSNFQALKNIINAFARIGDMLGGEELRQTVPMSPLQIYKTLLEYIQHSALSVGLKNLNQTQIGGQRVALAQTAEEASQRVYLSTVRVNDALSTRWETEDVFFTFMLKSMAAKIFIVLGIYDMFERPEPVYKLIPTRMILGGADELEPE :  900
AXB50010.1 : RSLSNFQALKNIINAFARIGDMLGGEELRQTVPMSPLQIYKTLLEYIQHSALSVGLKNLNQTQIGGQRVALAQTAEEASQRVYLSTVRVNDALSTRWETEDVFFTFMLKSMAAKIFIVLGIYDMFERPEPVYKLIPTRMILGGADELEPE :  900
AXB49666.1 : RSLSNFQALKNIINAFARIGDMLGGEELRQTVPMSPLQIYKTLLEYIQHSALSVGLKNLNQTQIGGQRVALAQTAEEASQRVYLSTVRVNDALSTRWETEDVFFTFMLKSMAAKIFIVLGIYDMFERPEPVYKLIPTRMILGGADELEPE :  900
CAD7112308 : RSLSNFQALKNIINAFARIGDMLGGEELRQTVPMSPLQIYKTLLEYIQHSALSVGLKNLNQTQIGGQRVALAQTAEEASQRVYLSTVRVNDALSTRWETEDVFFTFMLKSMAAKIFIVLGIYDMFERPEPVYKLIPTRMILGGADELEPE :  900
QRY19118.1 : RSLSNFQALKNIINAFARIGDMLGGEELRQTVPMSPLQIYKTLLEYIQHSALSVGLKNLNQTQIGGQRVALAQTAEEASQRVYLSTVRVNDALSTRWETEDVFFTFMLKSMAAKIFIVLGIYDMFERPEPVYKLIPTRMILGGADELEPE :  900
P0CA01.1   : RSLSNFQALKNIINAFARIGDMLGGEELRQTVPMSPLQIYKTLLEYIQHSALSVGLKNLNQTQIGGQRVALAQTAEEASQRVYLSTVRVNDALSTRWETEDVFFTFMLKSMAAKIFIVLGIYDMFERPEPVYKLIPTRMILGGADELEPE :  900
YP_0097028 : RSLSNFQALKNIINAFARIGDMLGGEELRQTVPMSPLQIYKTLLEYIQHSALSVGLKNLNQTQIGGQRVALAQTAEEASQRVYLSTVRVNDALSTRWETEDVFFTFMLKSMAAKIFIVLGIYDMFERPEPVYKLIPTRMILGGADELEPE :  900
P0CA03.1   : RSLSNFQALKNIINAFARIGDMLGGEELRQMVPMSPLQIYKTLLEYIQHSALSVGLKNLNQSEIGGQRVALARTPEEAAQRVYLSTVRVNDALSTRWETEDVFFTFMLKSMAAKIFIVLGIYDMFERPEPVYKLIPTRMILGGADELEPE :  900

                       *       920         *       940         *       960         *       980         *      1000         *      1020         *      1040         *
YP_0099272 : VIPEAAELYFRLPRLAEFYQKLFSFRDENVQISMLPELEGIFSGLIRIIFMRPIELINIGDYSETEIRQLIKEINVIYQHFNLEYGEQEATKKALIHFVNEINRRFGVITRTEWEKFQRIVQEARTMNDFGMMNQTNYSILPDEDGYTQS : 1050
AZP54139.1 : VIPEAAELYFRLPRLAEFYQKLFSFRDENVQISMLPELEGIFSGLIRIIFMRPIELINIGDYSETEIRQLIKEINVIYQHFNLEYGEQEATKKALIHFVNEINRRFGVITRTEWEKFQRIVQEARTMNDFGMMNQTNYSILPDEDGYTQS : 1050
QIE06881.1 : VIPEAAELYFRLPRLAEFYQKLFSFRDENVQISMLPELEGIFSGLIRIIFMRPIELINIGDYSETEIRQLIKEINVIYQHFNLEYGEQEATKKALIHFVNEINRRFGVITRTEWEKFQRIVQEARTMNDFGMMNQTNYSILPDEDGYTQS : 1050
QGJ83454.1 : VIPEAAELYFRLPRLAEFYQKLFSFRDENVQISMLPELEGIFSGLIRIIFMRPIELINIGDYSETEIRQLIKEINVIYQHFNLEYGEQEATKKALIHFVNEINRRFGVITRTEWEKFQRIVQEARTMNDFGMMNQTNYSILPDEDGYTQS : 1050
AYW34063.1 : VIPEAAELYFRLPRLAEFYQKLFSFRDENVQISMLPELEGIFSGLIRIIFMRPIELINIGDYSETEIRQLIKEINVIYQHFNLEYGEQEATKKALIHFVNEINRRFGVITRTEWEKFQRIVQEARTMNDFGMMNQTNYSILPDEDGYTQS : 1050
QLF78620.1 : VIPEAAELYFRLPRLAEFYQKLFSFRDENVQISMLPELEGIFSGLIRIIFMRPIELINIGDYSETEIRQLIKEINVIYQHFNLEYGEQEATKKALIHFVNEINRRFGVITRTEWEKFQRIVQEARTMNDFGMMNQTNYSILPDEDGYTQS : 1050
QTP96400.1 : VIPEAAELYFRLPRLAEFYQKLFSFRDENVQISMLPELEGIFSGLIRIIFMRPIELINIGDYSETEIRQLIKEINVIYQHFNLEYGEQEATKKALIHFVNEINRRFGVITRTEWEKFQRIVQEARTMNDFGMMNQTNYSILPDEDGYTQS : 1050
QBH90765.1 : VIPEAAELYFRLPRLAEFYQKLFSFRDENVQISMLPELEGIFSGLIRIIFMRPIELINIGDYSETEIRQLIKEINVIYQHFNLEYGEQEATKKALIHFVNEINRRFGVITRTEWEKFQRIVQEARTMNDFGMMNQTNYSILPDEDGYTQS : 1050
QOY24398.1 : VIPEAAELYFRLPRLAEFYQKLFSFRDENVQISMLPELEGIFSGLIRIIFMRPIELINIGDYSETEIRQLIKEINVIYQHFNLEYGEQEATKKALIHFVNEINRRFGVITRTEWEKFQRIVQEARTMNDFGMMNQTNYSILPDEDGYTQS : 1050
QSG73791.1 : VIPEAAELYFRLPRLAEFYQKLFSFRDENVQISMLPELEGIFSGLIRIIFMRPIELINIGDYSETEIRQLIKEINVIYQHFNLEYGEQEATKKALIHFVNEINRRFGVITRTEWEKFQRIVQEARTMNDFGMMNQTNYSILPDEDGYTQS : 1050
```

```
QTE18752.1 : VIPEAAELYFRLPRLAEFYQKLFSFRDENVQISMLPELEGIFSGLIRIIFMRPIELINIGDYSETEIRQLIKEINVIYQHFNLEYGEQEATKKALIHFVNEINRRFGVITRTEWEKFQRIVQEARTMNDFGMMNQTNYSILPDEDGYTQS : 1050
VFV48026.1 : VIPEAAELYFRLPRLAEFYQKLFSFRDENVQISMLPELEGIFSGLIRIIFMRPIELINIGDYSETEIRQLIKEINVIYQHFNLEYGEQEATKKALIHFVNEINRRFGVITRTEWEKFQRIVQEARTMNDFGMMNQTNYSILPDEDGYTQS : 1050
QOW03225.1 : VIPEAAELYFRLPRLAEFYQKLFSFRDENVQISMLPELEGIFSGLIRIIFMRPIELINIGDYSETEIRQLIKEINVIYQHFNLEYGEQEATKKALIHFVNEINRRFGVITRTEWEKFQRIVQEARTMNDFGMMNQTNYSILPDEDGYTQS : 1050
QOW03039.1 : VIPEAAELYFRLPRLAEFYQKLFSFRDENVQISMLPELEGIFSGLIRIIFMRPIELINIGDYSETEIRQLIKEINVIYQHFNLEYGEQEATKKALIHFVNEINRRFGVITRTEWEKFQRIVQEARTMNDFGMMNQTNYSILPDEDGYTQS : 1050
VVW94185.1 : VIPEAAELYFRLPRLAEFYQKLFSFRDENVQISMLPELEGIFSGLIRIIFMRPIELINIGDYSETEIRQLIKEINVIYQHFNLEYGEQEATKKALIHFVNEINRRFGVITRTEWEKFQRIVQEARTMNDFGMMNQTNYSILPDEDGYTQS : 1050
CAD0059543 : VIPEAAELYFRLPRLAEFYQKLFSFRDENVQISMLPELEGIFSGLIRIIFMRPIELINIGDYSETEIRQLIKEINVIYQHFNLEYGEQEATKKALIHFVNEINRRFGVITRTEWEKFQRIVQEARTMNDFGMMNQTNYSILPDEDGYTQS : 1050
QXP50051.1 : VIPEAAELYFRLPRLAEFYQKLFSFRDENVQISMLPELEGIFSGLIRIIFMRPIELINIGDYSETEIRQLIKEINVIYQHFNLEYGEQEATKKALIHFVNEINRRFGVITRTEWEKFQRIVQEARTMNDFGMMNQTNYSILPDEDGYTQS : 1050
CAD7112603 : VIPEAAELYFRLPRLAEFYQKLFSFRDENVQISMLPELEGIFSGLIRIIFMRPIELINIGDYSETEIRQLIKEINVIYQHFNLEYGEQEATKKALIHFVNEINRRFGVITRTEWEKFQRIVQEARTMNDFGMMNQTNYSILPDEDGYTQS : 1050
AXZ96054.1 : VIPEAAELYFRLPRLAEFYQKLFSFRDENVQISMLPELEGIFSGLIRIIFMRPIELINIGDYSETEIRQLIKEINVIYQHFNLEYGEQEATKKALIHFVNEINRRFGVITRTEWEKFQRIVQEARTMNDFGMMNQTNYSILPDEDGYTQS : 1050
QED90535.1 : VIPEAAELYFRLPRLAEFYQKLFSFRDENVQISMLPELEGIFSGLIRIIFMRPIELINIGDYSETEIRQLIKEINVIYQHFNLEYGEQEATKKALIHFVNEINRRFGVITRTEWEKFQRIVQEARTMNDFGMMNQTNYSILPDEDGYTQS : 1050
VVW94190.1 : VIPEAAELYFRLPRLAEFYQKLFSFRDENVQISMLPELEGIFSGLIRIIFMRPIELINIGDYSETEIRQLIKEINVIYQHFNLEYGEQEATKKALIHFVNEINRRFGVITRTEWEKFQRIVQEARTMNDFGMMNQTNYSILPDEDGYTQS : 1050
QEY87898.1 : VIPEAAELYFRLPRLAEFYQKLFSFRDENVQISMLPELEGIFSGLIRIIFMRPIELINIGDYSETEIRQLIKEINVIYQHFNLEYGEQEATKKALIHFVNEINRRFGVITRTEWEKFQRIVQEARTMNDFGMMNQTNYSILPDEDGYTQS : 1050
QGV56805.1 : VIPEAAELYFRLPRLAEFYQKLFSFRDENVQISMLPELEGIFSGLIRIIFMRPIELINIGDYSETEIRQLIKEINVIYQHFNLEYGEQEATKKALIHFVNEINRRFGVITRTEWEKFQRIVQEARTMNDFGMMNQTNYSILPDEDGYTQS : 1050
QPB67618.1 : VIPEAAELYFRLPRLAEFYQKLFSFRDENVQISMLPELEGIFSGLIRIIFMRPIELINIGDYSETEIRQLIKEINVIYQHFNLEYGEQEATKKALIHFVNEINRRFGVITRTEWEKFQRIVQEARTMNDFGMMNQTNYSILPDEDGYTQS : 1050
QUQ60377.1 : VIPEAAELYFRLPRLAEFYQKLFSFRDENVQISMLPELEGIFSGLIRIIFMRPIELINIGDYSETEIRQLIKEINVIYQHFNLEYGEQEATKKALIHFVNEINRRFGVITRTEWEKFQRIVQEARTMNDFGMMNQTNYSILPDEDGYTQS : 1050
QUQ60197.1 : VIPEAAELYFRLPRLAEFYQKLFSFRDENVQISMLPELEGIFSGLIRIIFMRPIELINIGDYSETEIRQLIKEINVIYQHFNLEYGEQEATKKALIHFVNEINRRFGVITRTEWEKFQRIVQEARTMNDFGMMNQTNYSILPDEDGYTQS : 1050
SPS73515.1 : VIPEAAELYFRLPRLAEFYQKLFSFRDENVQISMLPELEGIFSGLIRIIFMRPIELINIGDYSETEIRQLIKEINVIYQHFNLEYGEQEATKKALIHFVNEINRRFGVITRTEWEKFQRIVQEARTMNDFGMMNQTNYSILPDEDGYTQS : 1050
QDL88123.1 : VIPEAAELYFRLPRLAEFYQKLFSFRDENVQISMLPELEGIFSGLIRIIFMRPIELINIGDYSETEIRQLIKEINVIYQHFNLEYGEQEATKKALIHFVNEINRRFGVITRTEWEKFQRIVQEARTMNDFGMMNQTNYSILPDEDGYTQS : 1050
QIA61506.1 : VIPEAAELYFRLPRLAEFYQKLFSFRDENVQISMLPELEGIFSGLIRIIFMRPIELINIGDYSETEIRQLIKEINVIYQHFNLEYGEQEATKKALIHFVNEINRRFGVITRTEWEKFQRIVQEARTMNDFGMMNQTNYSILPDEDGYTQS : 1050
QTZ19752.1 : VIPEAAELYFRLPRLAEFYQKLFSFRDENVQISMLPELEGIFSGLIRIIFMRPIELINIGDYSETEIRQLIKEINVIYQHFNLEYGEQEATKKALIHFVNEINRRFGVITRTEWEKFQRIVQEARTMNDFGMMNQTNYSILPDEDGYTQS : 1050
QIM07715.1 : VIPEAAELYFRLPRLAEFYQKLFSFRDENVQISMLPELEGIFSGLIRIIFMRPIELINIGDYSETEIRQLIKEINVIYQHFNLEYGEQEATKKALIHFVNEINRRFGVITRTEWEKFQRIVQEARTMNDFGMMNQTNYSILPDEDGYTQS : 1050
QIM07948.1 : VIPEAAELYFRLPRLAEFYQKLFSFRDENVQISMLPELEGIFSGLIRIIFMRPIELINIGDYSETEIRQLIKEINVIYQHFNLEYGEQEATKKALIHFVNEINRRFGVITRTEWEKFQRIVQEARTMNDFGMMNQTNYSILPDEDGYTQS : 1050
QIM08183.1 : VIPEAAELYFRLPRLAEFYQKLFSFRDENVQISMLPELEGIFSGLIRIIFMRPIELINIGDYSETEIRQLIKEINVIYQHFNLEYGEQEATKKALIHFVNEINRRFGVITRTEWEKFQRIVQEARTMNDFGMMNQTNYSILPDEDGYTQS : 1050
YP_0097024 : VIPEAAELYFRLPRLAEFYQKLFSFRDENVQISMLPELEGIFSGLIRIIFMRPIELINIGDYSETEIRQLIKEINVIYQHFNLEYGEQEATKKALIHFVNEINRRFGVITRTEWEKFQRIVQEARTMNDFGMMNQTNYSILPDEDGYTQS : 1050
YP_0097038 : VIPEAAELYFRLPRLAEFYQKLFSFRDENVQISMLPELEGIFSGLIRIIFMRPIELINIGDYSETEIRQLIKEINVIYQHFNLEYGEQEATKKALIHFVNEINRRFGVITRTEWEKFQRIVQEARTMNDFGMMNQTNYSILPDEDGYTQS : 1050
YP_0097023 : VIPEAAELYFRLPRLAEFYQKLFSFRDENVQISMLPELEGIFSGLIRIIFMRPIELINIGDYSETEIRQLIKEINVIYQHFNLEYGEQEATKKALIHFVNEINRRFGVITRTEWEKFQRIVQEARTMNDFGMMNQTNYSILPDEDGYTQS : 1050
YP_0097026 : VIPEAAELYFRLPRLAEFYQKLFSFRDENVQISMLPELEGIFSGLIRIIFMRPIELINIGDYSETEIRQLIKEINVIYQHFNLEYGEQEATKKALIHFVNEINRRFGVITRTEWEKFQRIVQEARTMNDFGMMNQTNYSILPDEDGYTQS : 1050
YP_0097035 : VIPEAAELYFRLPRLAEFYQKLFSFRDENVQISMLPELEGIFSGLIRIIFMRPIELINIGDYSETEIRQLIKEINVIYQHFNLEYGEQEATKKALIHFVNEINRRFGVITRTEWEKFQRIVQEARTMNDFGMMNQTNYSILPDEDGYTQS : 1050
QZK26792.1 : VIPEAAELYFRLPRLAEFYQKLFSFRDENVQISMLPELEGIFSGLIRIIFMRPIELINIGDYSETEIRQLIKEINVIYQHFNLEYGEQEATKKALIHFVNEINRRFGVITRTEWEKFQRIVQEARTMNDFGMMNQTNYSILPDEDGYTQS : 1050
YP_0097036 : VIPEAAELYFRLPRLAEFYQKLFSFRDENVQISMLPELEGIFSGLIRIIFMRPIELINIGDYSETEIRQLIKEINVIYQHFNLEYGEQEATKKALIHFVNEINRRFGVITRTEWEKFQRIVQEARTMNDFGMMNQTNYSILPDEDGYTQS : 1050
QID21266.1 : VIPEAAELYFRLPRLAEFYQKLFSFRDENVQISMLPELEGIFSGLIRIIFMRPIELINIGDYSETEIRQLIKEINVIYQHFNLEYGEQEATKKALIHFVNEINRRFGVITRTEWEKFQRIVQEARTMNDFGMMNQTNYSILPDEDGYIQS : 1050
P0CA04.1   : VIPEAAELYFRLPRLAEFYQKLFSFRDENVQISMLPELEGIFSGLIRIIFMRPIELINIGDYSETEIRQLIKEINVIYQHFNLEYGEQEATKKALIHFVNEINRRFGVITRTEWEKFQRIVQEARTMNDFGMMNQTNYSILPDEDGYTQS : 1050
P0CA02.1   : VIPEAAELYFRLPRLAEFYQKLFSFRDGNVQISMLPELEGIFSGLIRIIFMRPIELINIGDYSETEIRQLIKEINVIYQHFNLEYGEQEATKKALIHFVNEINRRFGVITRTEWEKFQRIVQEARTMNDFGMMNQTNYSILPDEDGYTQS : 1050
YP_0097029 : VIPEAAGLYFRLPRLAEFYQKLFSFRDENVQISMLPELEGIFSGLIRVIFMRPIELINIGDYSETEIRQLIKEINVIYQHFNLEYGEQEAVKKALIHFVNEINRRFGVITRTEWEKFQRIVQEARTMNDFGMMNQTNYSILPDEDGYTQS : 1050
AXB50010.1 : VIPEAAGLYFRLPRLAEFYQKLFSFRDENVQISMLPELEGIFSGLIRVIFMRPIELINIGDYSETEIRQLIKEINVIYQHFNLEYGEQEAVKKALIHFVNEINRRFGVITRTEWEKFQRIVQEARTMNDFGMMNQTNYSILPDEDGYTQS : 1050
AXB49666.1 : VIPEAAGLYFRLPRLAEFYQKLFSFRDENVQISMLPELEGIFSGLIRVIFMRPIELINIGDYSETEIRQLIKEINVIYQHFNLEYGEQEAVKKALIHFVNEINRRFGVITRTEWEKFQRIVQEARTMNDFGMMNQTNYSILPDEDGYTQS : 1050
CAD7112308 : VIPEAAGLYFRLPRLAEFYQKLFSFRDENVQISMLPELEGIFSGLIRVIFMRPIELINIGDYSETEIRQLIKEINVIYQHFNLEYGEQEAVKKALIHFVNEINRRFGVITRTEWEKFQRIVQEARTMNDFGMMNQTNYSILPDEDGYTQS : 1050
QRY19118.1 : VIPEAAGLYFRLPRLAEFYQKLFSFRDENVQISMLPELEGIFSGLIRVIFMRPIELINIGDYSETEIRQLIKEINVIYQHFNLEYGEQEAVKKALIHFVNEINRRFGVITRTEWEKFQRIVQEARTMNDFGMMNQTNYSILPDEDGYTQS : 1050
P0CA01.1   : VIPEAAGLYFRLPRLAEFYQKLFSFRDENVQISMLPELEGIFSGLIRVIFMRPIELINIGDYSETEIRQLIKEINVIYQHFNLEYGEQEAVKKALIHFVNEINRRFGVITRTEWEKFQRIVQEARTMNDFGMMNQTNYSILPDEDGYTQS : 1050
YP_0097028 : VIPEAAGLYFRLPRLAEFYQKLFSFRDENVQISMLPELEGIFSGLIRVIFMRPIELINIGDYSETEIRQLIKEINVIYQHFNLEYGEQEAVKKALIHFVNEINRRFGVITRTEWEKFQRIVQEARTMNDFGMMNQTNYSILPDEDGYTQS : 1050
P0CA03.1   : VIPEAAELYFRLPRLAEFYQKLFSFRDENVQISMLPELEGIFSGLIRIIFMRPIELINIGDYSETEIRQLIKEINVIYQHFNLEYGEQEATKKALIHFVNEINRRFGVITRTEWEKFQRIVQEARTMNDFGMMNQTNYSILPDEDGYTQS : 1050

                    1060          *        1080          *        1100          *        1120          *        1140          *        1160          *        1180          *        1200
YP_0099272 : SQLLPSDRFISPSTQPTPKWRPALYNIDSVDVQTGMLQPNSQWDLVQKFRKQLSEMFEDPSLQQELGKISYQELIRQAINELKKEHTDKIQIVSKLIQGSESLADTDVNKIFLFHETVITGLNLLSAIYVLLNNFRNNIKGLDLDTIQKS : 1200
AZP54139.1 : SQLLPSDRFISPSTQPTPKWRPALYNIDSVDVQTGMLQPNSQWDLVQKFRKQLSEMFEDPSLQQELGKISYQELIRQAINELKKEHTDKIQIVSKLIQGSESLADTDVNKIFLFHETVITGLNLLSAIYVLLNNFRNNIKGLDLDTIQKS : 1200
QIE06881.1 : SQLLPSDRFISPSTQPTPKWRPALYNIDSVDVQTGMLQPNSQWDLVQKFRKQLSEMFEDPSLQQELGKISYQELIRQAINELKKEHTDKIQIVSKLIQGSESLADTDVNKIFLFHETVITGLNLLSAIYVLLNNFRNNIKGLDLDTIQKS : 1200
QGJ83454.1 : SQLLPSDRFISPSTQPTPKWRPALYNIDSVDVQTGMLQPNSQWDLVQKFRKQLSEMFEDPSLQQELGKISYQELIRQAINELKKEHTDKIQIVSKLIQGSESLADTDVNKIFLFHETVITGLNLLSAIYVLLNNFRNNIKGLDLDTIQKS : 1200
AYW34063.1 : SQLLPSDRFISPSTQPTPKWRPALYNIDSVDVQTGMLQPNSQWDLVQKFRKQLSEMFEDPSLQQELGKISYQELIRQAINELKKEHTDKIQIVSKLIQGSESLADTDVNKIFLFHETVITGLNLLSAIYVLLNNFRNNIKGLDLDTIQKS : 1200
QLF78620.1 : SQLLPSDRFISPSTQPTPKWRPALYNIDSVDVQTGMLQPNSQWDLVQKFRKQLSEMFEDPSLQQELGKISYQELIRQAINELKKEHTDKIQIVSKLIQGSESLADTDVNKIFLFHETVITGLNLLSAIYVLLNNFRNNIKGLDLDTIQKS : 1200
QTP96400.1 : SQLLPSDRFISPSTQPTPKWRPALYNIDSVDVQTGMLQPNSQWDLVQKFRKQLSEMFEDPSLQQELGKISYQELIRQAINELKKEHTDKIQIVSKLIQGSESLADTDVNKIFLFHETVITGLNLLSAIYVLLNNFRNNIKGLDLDTIQKS : 1200
QBH90765.1 : SQLLPSDRFISPSTQPTPKWRPALYNIDSVDVQTGMLQPNSQWDLVQKFRKQLSEMFEDPSLQQELGKISYQELIRQAINELKKEHTDKIQIVSKLIQGSESLADTDVNKIFLFHETVITGLNLLSAIYVLLNNFRNNIKGLDLDTIQKS : 1200
QOY24398.1 : SQLLPSDRFISPSTQPTPKWRPALYNIDSVDVQTGMLQPNSQWDLVQKFRKQLSEMFEDPSLQQELGKISYQELIRQAINELKKEHTDKIQIVSKLIQGSESLADTDVNKIFLFHETVITGLNLLSAIYVLLNNFRNNIKGLDLDTIQKS : 1200
QSG73791.1 : SQLLPSDRFISPSTQPTPKWRPALYNIDSVDVQTGMLQPNSQWDLVQKFRKQLSEMFEDPSLQQELGKISYQELIRQAINELKKEHTDKIQIVSKLIQGSESLADTDVNKIFLFHETVITGLNLLSAIYVLLNNFRNNIKGLDLDTIQKS : 1200
QTE18752.1 : SQLLPSDRFISPSTQPTPKWRPALYNIDSVDVQTGMLQPNSQWDLVQKFRKQLSEMFEDPSLQQELGKISYQELIRQAINELKKEHTDKIQIVSKLIQGSESLADTDVNKIFLFHETVITGLNLLSAIYVLLNNFRNNIKGLDLDTIQKS : 1200
VFV48026.1 : SQLLPSDRFISPSTQPTPKWRPALYNIDSVDVQTGMLQPNSQWDLVQKFRKQLSEMFEDPSLQQELGKISYQELIRQAINELKKEHTDKIQIVSKLIQGSESLADTDVNKIFLFHETVITGLNLLSAIYVLLNNFRNNIKGLDLDTIQKS : 1200
QOW03225.1 : SQLLPSDRFISPSTQPTPKWRPALYNIDSVDVQTGMLQPNSQWDLVQKFRKQLSEMFEDPSLQQELGKISYQELIRQAINELKKEHTDKIQIVSKLIQGSESLADTDVNKIFLFHETVITGLNLLSAIYVLLNNFRNNIKGLDLDTIQKS : 1200
QOW03039.1 : SQLLPSDRFISPSTQPTPKWRPALYNIDSVDVQTGMLQPNSQWDLVQKFRKQLSEMFEDPSLQQELGKISYQELIRQAINELKKEHTDKIQIVSKLIQGSESLADTDVNKIFLFHETVITGLNLLSAIYVLLNNFRNNIKGLDLDTIQKS : 1200
VVW94185.1 : SQLLPSDRFISPSTQPTPKWRPALYNIDSVDVQTGMLQPNSQWDLVQKFRKQLSEMFEDPSLQQELGKISYQELIRQAINELKKEHTDKIQIVSKLIQGSESLADTDVNKIFLFHETVITGLNLLSAIYVLLNNFRNNIKGLDLDTIQKS : 1200
CAD0059543 : SQLLPSDRFISPSTQPTPKWRPALYNIDSVDVQTGMLQPNSQWDLVQKFRKQLSEMFEDPSLQQELGKISYQELIRQAINELKKEHTDKIQIVSKLIQGSESLADTDVNKIFLFHETVITGLNLLSAIYVLLNNFRNNIKGLDLDTIQKS : 1200
QXP50051.1 : SQLLPSDRFISPSTQPTPKWRPALYNIDSVDVQTGMLQPNSQWDLVQKFRKQLSEMFEDPSLQQELGKISYQELIRQAINELKKEHTDKIQIVSKLIQGSESLADTDVNKIFLFHETVITGLNLLSAIYVLLNNFRNNIKGLDLDTIQKS : 1200
CAD7112603 : SQLLPSDRFISPSTQPTPKWRPALYNIDSVDVQTGMLQPNSQWDLVQKFRKQLSEMFEDPSLQQELGKISYQELIRQAINELKKEHTDKIQIVSKLIQGSESLADTDVNKIFLFHETVITGLNLLSAIYVLLNNFRNNIKGLDLDTIQKS : 1200
AXZ96054.1 : SQLLPSDRFISPSTQPTPKWRPALYNIDSVDVQTGMLQPNSQWDLVQKFRKQLSEMFEDPSLQQELGKISYQELIRQAINELKKEHTDKIQIVSKLIQGSESLADTDVNKIFLFHETVITGLNLLSAIYVLLNNFRNNIKGLDLDTIQKS : 1200
QED90535.1 : SQLLPSDRFISPSTQPTPKWRPALYNIDSVDVQTGMLQPNSQWDLVQKFRKQLSEMFEDPSLQQELGKISYQELIRQAINELKKEHTDKIQIVSKLIQGSESLADTDVNKIFLFHETVITGLNLLSAIYVLLNNFRNNIKGLDLDTIQKS : 1200
VVW94190.1 : SQLLPSDRFISPSTQPTPKWRPALYNIDSVDVQTGMLQPNSQWDLVQKFRKQLSEMFEDPSLQQELGKISYQELIRQAINELKKEHTDKIQIVSKLIQGSESLADTDVNKIFLFHETVITGLNLLSAIYVLLNNFRNNIKGLDLDTIQKS : 1200
QEY87898.1 : SQLLPSDRFISPSTQPTPKWRPALYNIDSVDVQTGMLQPNSQWDLVQKFRKQLSEMFEDPSLQQELGKISYQELIRQAINELKKEHTDKIQIVSKLIQGSESLADTDVNKIFLFHETVITGLNLLSAIYVLLNNFRNNIKGLDLDTIQKS : 1200
QGV56805.1 : SQLLPSDRFISPSTQPTPKWRPALYNIDSVDVQTGMLQPNSQWDLVQKFRKQLSEMFEDPSLQQELGKISYQELIRQAINELKKEHTDKIQIVSKLIQGSESLADTDVNKIFLFHETVITGLNLLSAIYVLLNNFRNNIKGLDLDTIQKS : 1200
QPB67618.1 : SQLLPSDRFISPSTQPTPKWRPALYNIDSVDVQTGMLQPNSQWDLVQKFRKQLSEMFEDPSLQQELGKISYQELIRQAINELKKEHTDKIQIVSKLIQGSESLADTDVNKIFLFHETVITGLNLLSAIYVLLNNFRNNIKGLDLDTIQKS : 1200
```

```
QUQ60377.1 : SQLLPSDRFISPSTQPTPKWRPALYNIDSVDVQTGMLQPNSQWDLVQKFRKQLSEMFEDPSLQQELGKISYQELIRQAINELKKEHTDKIQIVSKLIQGSESLADTDVNKIFLFHETVITGLNLLSAIYVLLNNFRNNIKGLDLDTIQKS : 1200
QUQ60197.1 : SQLLPSDRFISPSTQPTPKWRPALYNIDSVDVQTGMLQPNSQWDLVQKFRKQLSEMFEDPSLQQELGKISYQELIRQAINELKKEHTDKIQIVSKLIQGSESLADTDVNKIFLFHETVITGLNLLSAIYVLLNNFRNNIKGLDLDTIQKS : 1200
SPS73515.1 : SQLLPSDRFISPSTQPTPKWRPALYNIDSVDVQTGMLQPNSQWDLVQKFRKQLSEMFEDPSLQQELGKISYQELIRQAINELKKEHTDKIQIVSKLIQGSESLADTDVNKIFLFHETVITGLNLLSAIYVLLNNFRNNIKGLDLDTIQKS : 1200
QDL88123.1 : SQLLPSDRFISPSTQPTPKWRPALYNIDSVDVQTGMLQPNSQWDLVQKFRKQLSEMFEDPSLQQELGKISYQELIRQAINELKKEHTDKIQIVSKLIQGSESLADTDVNKIFLFHETVITGLNLLSAIYVLLNNFRNNIKGLDLDTIQKS : 1200
QIA61506.1 : SQLLPSDRFISPSTQPTPKWRPALYNIDSVDVQTGMLQPNSQWDLVQKFRKQLSEMFEDPSLQQELGKISYQELIRQAINELKKEHTDKIQIVSKLIQGSESLADTDVNKIFLFHETVITGLNLLSAIYVLLNNFRNNIKGLDLDTIQKS : 1200
QTZ19752.1 : SQLLPSDRFISPSTQPTPKWRPALYNIDSVDVQTGMLQPNSQWDLVQKFRKQLSEMFEDPSLQQELGKISYQELIRQAINELKKEHTDKIQIVSKLIQGSESLADTDVNKIFLFHETVITGLNLLSAIYVLLNNFRNNIKGLDLDTIQKS : 1200
QIM07715.1 : SQLLPSDRFISPSTQPTPKWRPALYNIDSVDVQTGMLQPNSQWDLVQKFRKQLSEMFEDPSLQQELGKVSYQELIRQAINELKKEHTDKIQIVSKLIQGSESLADTDVNKIFLFHETVITGLNLLSAIYVLLNNFRNNIKGLDLDTIQKS : 1200
QIM07948.1 : SQLLPSDRFISPSTQPTPKWRPALYNIDSVDVQTGMLQPNSQWDLVQKFRKQLSEMFEDPSLQQELGKVSYQELIRQAINELKKEHTDKIQIVSKLIQGSESLADTDVNKIFLFHETVITGLNLLSAIYVLLNNFRNNIKGLDLDTIQKS : 1200
QIM08183.1 : SQLLPSDRFISPSTQPTPKWRPALYNIDSVDVQTGMLQPNSQWDLVQKFRKQLSEMFEDPSLQQELGKVSYQELIRQAINELKKEHTDKIQIVSKLIQGSESLADTDVNKIFLFHETVITGLNLLSAIYVLLNNFRNNIKGLDLDTIQKS : 1200
YP_0097024 : SQLLPSDRFISPSTQPTPKWRPALYNIDSVDVQTGMLQPNSQWDLVQKFRKQLSEMFEDPSLQQELGKVSYQELIRQAINELKKEHTDKIQIVSKLIQGSESLADTDVNKIFLFHETVITGLNLLSAIYVLLNNFRNNIKGLDLDTIQKS : 1200
YP_0097038 : SQLLPSDRFISPSTQPTPKWRPALYNIDSVDVQTGMLQPNSQWDLVQKFRKQLSEMFEDPSLQQELGKVSYQELIRQAINELKKEHTDKIQIVSKLIQGSESLADTDVNKIFLFHETVITGLNLLSAIYVLLNNFRNNIKGLDLDTIQKS : 1200
YP_0097023 : SQLLPSDRFISPSTQPTPKWRPALYNIDSVDVQTGMLQPNSQWDLVQKFRKQLSEMFEDPSLQQELGKVSYQELIRQAINELKKEHTDKIQIVSKLIQGSESLADTDVNKIFLFHETVITGLNLLSAIYVLLNNFRNNIKGLDLDTIQKS : 1200
YP_0097026 : SQLLPSDRFISPSTQPTPKWRPALYNIDSVDVQTGMLQPNSQWDLVQKFRKQLSEMFEDPSLQQELGKVSYQELIRQAINELKKEHTDKIQIVSKLIQGSESLADTDVNKIFLFHETVITGLNLLSAIYVLLNNFRNNIKGLDLDTIQKS : 1200
YP_0097035 : SQLLPSDRFISPSTQPTPKWRPALYNIDSVDVQTGMLQPNSQWDLVQKFRKQLSEMFEDPSLQQELGKVSYQELIRQAINELKKEHTDKIQIVSKLIQGSESLADTDVNKIFLFHETVITGLNLLSAIYVLLNNFRNNIKGLDLDTIQKS : 1200
QZK26792.1 : SQLLPSDRFISPSTQPTPKWRPALYNIDSVDVQTGMLQPNSQWDLVQKFRKQLSEMFEDPSLQQELGKISYQELIRQAINELKKEHTDKIQIVSKLIQGSESLADTDVNKIFLFHETVITGLNLLSAIYVLLNNFRNNIKGLDLDTIQKS : 1200
YP_0097036 : SQLLPSDRFISPSTQPTPKWRPALYNIDSVDVQTGMLQPNSQWDLVQKFRKQLSEMFEDPSLQQELGKVSYQELIRQAINELKKEHTDKIQIVSKLIQGSESLADTDVNKIFLFHETVITGLNLLSAIYVLLNNFRNNIKGLDLDTIQKS : 1200
QID21266.1 : SQLLPSDRFISPSTQPSPKWRPALYNIDSVDVQTGMLQPNSQWDLVQKFRKQLSEMFEDPSLQQELGKVSYQELIRQAINELKKEHTDKIQIVSKLIQGSESLADTDVNKIFLFHETVITGLNLLSAIYVLLNNFRNNIKGLDLDTIQKS : 1200
P0CA04.1   : SQLLPSDRFISPSSQPTPKWRPALYNIDSVDVQTGMLQPNSQWDLVQKFRKQLSEMFEDPSLQQELGKVSYQELIRQAINELKKDHTDKIQIVSKLIQGSESLADTDVNKIFLFHETVITGLNLLSAIYVLLNNFRNNIKGLDLDTIQKS : 1200
P0CA02.1   : SQLLPSDRFIGPSSQPTPKWRPALYNIDSVDVQTGMLQPNSQWDLVQKFRKQLSEMFEDPSLQQELGKISYQELIRQAINELKKEHTDKIQIVSKLIQGSESLADTDVNKIFLFHETVITGLNLLSAIYVLLNNFRNNIKGLDLDTIQKS : 1200
YP_0097029 : SQLLPSDRFIGPSSQPTPKWRPALYNIDSVDVQTGMLQPNSQWDLVQKFRKQLSEMFEDPSLQQELGKVSYQELIQQATNELKKEHTDKIQIVSKLIQGSESLADTDVNKIFLFHETVITGLNLLSAIYVLLNTFRNNIKALDLDMIQKS : 1200
AXB50010.1 : SQLLPSDRFIGPSSQPTPKWRPALYNIDSVDVQTGMLQPNSQWDLVQKFRKQLSEMFEDPSLQQELGKVSYQELIQQATNELKKEHTDKIQIVSKLIQGSESLADTDVNKIFLFHETVITGLNLLSAIYVLLNTFRNNIKALDLDMIQKS : 1200
AXB49666.1 : SQLLPSDRFIGPSSQPTPKWRPALYNIDSVDVQTGMLQPNSQWDLVQKFRKQLSEMFEDPSLQQELGKVSYQELIQQATNELKKEHTDKIQIVSKLIQGSESLADTDVNKIFLFHETVITGLNLLSAIYVLLNTFRNNIKALDLDMIQKS : 1200
CAD7112308 : SQLLPSDRFIGPSSQPTPKWRPALYNIDSVDVQTGMLQPNSQWDLVQKFRKQLSEMFEDPSLQQELGKVSYQELIQQATNELKKEHTDKIQIVSKLIQGSESLADTDVNKIFLFHETVITGLNLLSAIYVLLNTFRNNIKALDLDTIQKS : 1200
QRY19118.1 : SQLLPSDRFIGPSSQPTPKWRPALYNIDSVDVQTGMLQPNSQWDLVQKFRKQLSEMFEDPSLQQELGKVSYQELIQQATNELKKEHTDKIQIVSKLIQGSESLADTDVNKIFLFHETVITGLNLLSAIYVLLNTFRNNIKALDLDTIQKS : 1200
P0CA01.1   : SQLLPSDRFIGPSSQPTPKWRPALYNIDSVDVQTGMLQPNSQWDLVQKFRKQLSEMFEDPSLQQELGKVSYQELIQQATNELKKEHTDKIQIVSKLIQGSESLADTDVNKIFLFHETVITGLNLLSAIYVLLNTFRNNIKALDLDTIQKS : 1200
YP_0097028 : SQLLPSDRFIGPSSQPTPKWRPALYNIDSVDVQTGMLQPNSQWDLVQKFRKQLSEMFEDPSLQQELGKVSYQELIQQATNELKKEHTDKIQIVSKLIQGSESLADTDVNKIFLFHETVITGLNLLSAIYVLLNTFRNNIKALDLDTIQKS : 1200
P0CA03.1   : SQLLPSDRFISPSSQPTPKWRPALYNIDSVDVQTGMLQPNSQWDLVQKFRKQLSEMFEDPSLQQELGKVSYQELIRQAINELKKDHTDKIQIVSKLIQGSESLADTDVNKIFLFHETVITGLNLLSAIYVLLNNFRNNIKGLDLDTIQKS : 1200

                     *        1220         *        1240         *        1260         *        1280         *        1300         *        1320         *        1340         *
YP_0099272 : IIEWLRETQAANVNRANLIDWLGRKHGAISEIRNPGLVIKEINMRLSMVYPDPTTEAAAAAQDRNLTTETLFAWIVPYVGIPAGGGVRPEQELAARYLVDNQRIMQLLLTNIFEMTSSFNKMVQVRFPETSTAQVHLDFTGLISLIDSLM : 1350
AZP54139.1 : IIEWLRETQAANVNRANLIDWLGRKHGAISEIRNPGLVIKEINMRLSMVYPDPTTEAAAAAQDRNLTTETLFAWIVPYVGIPAGGGVRPEQELAARYLVDNQRIMQLLLTNIFEMTSSFNKMVQVRFPETSTAQVHLDFTGLISLIDSLM : 1350
QIE06881.1 : IIEWLRETQAANVNRANLIDWLGRKHGAISEIRNPGLVIKEINMRLSMVYPDPTTEAAAAAQDRNLTTETLFAWIVPYVGIPAGGGVRPEQELAARYLVDNQRIMQLLLTNIFEMTSSFNKMVQVRFPETSTAQVHLDFTGLISLIDSLM : 1350
QGJ83454.1 : IIEWLRETQAANVNRANLIDWLGRKHGAISEIRNPGLVIKEINMRLSMVYPDPTTEAAAAAQDRNLTTETLFAWIVPYVGIPAGGGVRPEQELAARYLVDNQRIMQLLLTNIFEMTSSFNKMVQVRFPETSTAQVHLDFTGLISLIDSLM : 1350
AYW34063.1 : IIEWLRETQAANVNRANLIDWLGRKHGAISEIRNPGLVIKEINMRLSMVYPDPTTEAAAAAQDRNLTTETLFAWIVPYVGIPAGGGVRPEQELAARYLVDNQRIMQLLLTNIFEMTSSFNKMVQVRFPETSTAQVHLDFTGLISLIDSLM : 1350
QLF78620.1 : IIEWLRETQAANVNRANLIDWLGRKHGAISEIRNPGLVIKEINMRLSMVYPDPTTEAAAAAQDRNLTTETLFAWIVPYVGIPAGGGVRPEQELAARYLVDNQRIMQLLLTNIFEMTSSFNKMVQVRFPETSTAQVHLDFTGLISLIDSLM : 1350
QTP96400.1 : IIEWLRETQAANVNRANLIDWLGRKHGAISEIRNPGLVIKEINMRLSMVYPDPTTEAAAAAQDRNLTTETLFAWIVPYVGIPAGGGVRPEQELAARYLVDNQRIMQLLLTNIFEMTSSFNKMVQVRFPETSTAQVHLDFTGLISLIDSLM : 1350
QBH90765.1 : IIEWLRETQAANVNRANLIDWLGRKHGAISEIRNPGLVIKEINMRLSMVYPDPTTEAAAAAQDRNLTTETLFAWIVPYVGIPAGGGVRPEQELAARYLVDNQRIMQLLLTNIFEMTSSFNKMVQVRFPETSTAQVHLDFTGLISLIDSLM : 1350
QOY24398.1 : IIEWLRETQAANVNRANLIDWLGRKHGAISEIRNPGLVIKEINMRLSMVYPDPTTEAAAAAQDRNLTTETLFAWIVPYVGIPAGGGVRPEQELAARYLVDNQRIMQLLLTNIFEMTSSFNKMVQVRFPETSTAQVHLDFTGLISLIDSLM : 1350
QSG73791.1 : IIEWLRETQAANVNRANLIDWLGRKHGAISEIRNPGLVIKEINMRLSMVYPDPTTEAAAAAQDRNLTTETLFAWIVPYVGIPAGGGVRPEQELAARYLVDNQRIMQLLLTNIFEMTSSFNKMVQVRFPETSTAQVHLDFTGLISLIDSLM : 1350
QTE18752.1 : IIEWLRETQAANVNRANLIDWLGRKHGAISEIRNPGLVIKEINMRLSMVYPDPTTEAAAAAQDRNLTTETLFAWIVPYVGIPAGGGVRPEQELAARYLVDNQRIMQLLLTNIFEMTSSFNKMVQVRFPETSTAQVHLDFTGLISLIDSLM : 1350
VFV48026.1 : IIEWLRETQAANVNRANLIDWLGRKHGAISEIRNPGLVIKEINMRLSMVYPDPTTEAAAAAQDRNLTTETLFAWIVPYVGIPAGGGVRPEQELAARYLVDNQRIMQLLLTNIFEMTSSFNKMVQVRFPETSTAQVHLDFTGLISLIDSLM : 1350
QOW03225.1 : IIEWLRETQAANVNRANLIDWLGRKHGAISEIRNPGLVIKEINMRLSMVYPDPTTEAAAAAQDRNLTTETLFAWIVPYVGIPAGGGVRPEQELAARYLVDNQRIMQLLLTNIFEMTSSFNKMVQVRFPETSTAQVHLDFTGLISLIDSLM : 1350
QOW03039.1 : IIEWLRETQAANVNRANLIDWLGRKHGAISEIRNPGLVIKEINMRLSMVYPDPTTEAAAAAQDRNLTTETLFAWIVPYVGIPAGGGVRPEQELAARYLVDNQRIMQLLLTNIFEMTSSFNKMVQVRFPETSTAQVHLDFTGLISLIDSLM : 1350
VVW94185.1 : IIEWLRETQAANVNRANLIDWLGRKHGAISEIRNPGLVIKEINMRLSMVYPDPTTEAAAAAQDRNLTTETLFAWIVPYVGIPAGGGVRPEQELAARYLVDNQRIMQLLLTNIFEMTSSFNKMVQVRFPETSTAQVHLDFTGLISLIDSLM : 1350
CAD0059543 : IIEWLRETQAANVNRANLIDWLGRKHGAISEIRNPGLVIKEINMRLSMVYPDPTTEAAAAAQDRNLTTETLFAWIVPYVGIPAGGGVRPEQELAARYLVDNQRIMQLLLTNIFEMTSSFNKMVQVRFPETSTAQVHLDFTGLISLIDSLM : 1350
QXP50051.1 : IIEWLRETQAANVNRANLIDWLGRKHGAISEIRNPGLVIKEINMRLSMVYPDPTTEAAAAAQDRNLTTETLFAWIVPYVGIPAGGGVRPEQELAARYLVDNQRIMQLLLTNIFEMTSSFNKMVQVRFPETSTAQVHLDFTGLISLIDSLM : 1350
CAD7112603 : IIEWLRETQAANVNRANLIDWLGRKHGAISEIRNPGLVIKEINMRLSMVYPDPTTEAAAAAQDRNLTTETLFAWIVPYVGIPAGGGVRPEQELAARYLVDNQRIMQLLLTNIFEMTSSFNKMVQVRFPETSTAQVHLDFTGLISLIDSLM : 1350
AXZ96054.1 : IIEWLRETQAANVNRANLIDWLGRKHGAISEIRNPGLVIKEINMRLSMVYPDPTTEAAAAAQDRNLTTETLFAWIVPYVGIPAGGGVRPEQELAARYLVDNQRIMQLLLTNIFEMTSSFNKMVQVRFPETSTAQVHLDFTGLISLIDSLM : 1350
QED90535.1 : IIEWLRETQAANVNRANLIDWLGRKHGAISEIRNPGLVIKEINMRLSMVYPDPTTEAAAAAQDRNLTTETLFAWIVPYVGIPAGGGVRPEQELAARYLVDNQRIMQLLLTNIFEMTSSFNKMVQVRFPETSTAQVHLDFTGLISLIDSLM : 1350
VVW94190.1 : IIEWLRETQAANVNRANLIDWLGRKHGAISEIRNPGLVIKEINMRLSMVYPDPTTEAAAAAQDRNLTTETLFAWIVPYVGIPAGGGVRPEQELAARYLVDNQRIMQLLLTNIFEMTSSFNKMVQVRFPETSTAQVHLDFTGLISLIDSLM : 1350
QEY87898.1 : IIEWLRETQAANVNRANLIDWLGRKHGAISEIRNPGLVIKEINMRLSMVYPDPTTEAAAAAQDRNLTTETLFAWIVPYVGIPAGGGVRPEQELAARYLVDNQRIMQLLLTNIFEMTSSFNKMVQVRFPETSTAQVHLDFTGLISLIDSLM : 1350
QGV56805.1 : IIEWLRETQAANVNRANLIDWLGRKHGAISEIRNPGLVIKEINMRLSMVYPDPTTEAAAAAQDRNLTTETLFAWIVPYVGIPAGGGVRPEQELAARYLVDNQRIMQLLLTNIFEMTSSFNKMVQVRFPETSTAQVHLDFTGLISLIDSLM : 1350
QPB67618.1 : IIEWLRETQAANVNRANLIDWLGRKHGAISEIRNPGLVIKEINMRLSMVYPDPTTEAAAAAQDRNLTTETLFAWIVPYVGIPAGGGVRPEQELAARYLVDNQRIMQLLLTNIFEMTSSFNKMVQVRFPETSTAQVHLDFTGLISLIDSLM : 1350
QUQ60377.1 : IIEWLRETQAANVNRANLIDWLGRKHGAISEIRNPGLVIKEINMRLSMVYPDPTTEAAAAAQDRNLTTETLFAWIVPYVGIPAGGGVRPEQELAARYLVDNQRIMQLLLTNIFEMTSSFNKMVQVRFPETSTAQVHLDFTGLISLIDSLM : 1350
QUQ60197.1 : IIEWLRETQAANVNRANLIDWLGRKHGAISEIRNPGLVIKEINMRLSMVYPDPTTEAAAAAQDRNLTTETLFAWIVPYVGIPAGGGVRPEQELAARYLVDNQRIMQLLLTNIFEMTSSFNKMVQVRFPETSTAQVHLDFTGLISLIDSLM : 1350
SPS73515.1 : IIEWLRETQAANVNRANLIDWLGRKHGAISEIRNPGLVIKEINMRLSMVYPDPTTEAAAAAQDRNLTTETLFAWIVPYVGIPAGGGVRPEQELAARYLVDNQRIMQLLLTNIFEMTSSFNKMVQVRFPETSTAQVHLDFTGLISLIDSLM : 1350
QDL88123.1 : IIEWLRETQAANVNRANLIDWLGRKHGAISEIRNPGLVIKEINMRLSMVYPDPTTEAAAAAQDRNLTTETLFAWIVPYVGIPAGGGVRPEQELAARYLVDNQRIMQLLLTNIFEMTSSFNKMVQVRFPETSTAQVHLDFTGLISLIDSLM : 1350
QIA61506.1 : IIEWLRETQAANVNRANLIDWLGRKHGAISEIRNPGLVIKEINMRLSMVYPDPTTEAAAAAQDRNLTTETLFAWIVPYVGIPAGGGVRPEQELAARYLVDNQRIMQLLLTNIFEMTSSFNKMVQVRFPETSTAQVHLDFTGLISLIDSLM : 1350
QTZ19752.1 : IIEWLRETQAANVNRANLIDWLGRKHGAISEIRNPGLVIKEINMRLSMVYPDPTTEAAAAAQDRNLTTETLFAWIVPYVGIPAGGGVRPEQELAARYLVDNQRIMQLLLTNIFEMTSSFNKMVQVRFPETSTAQVHLDFTGLISLIDSLM : 1350
QIM07715.1 : IIEWLRETQAANVNRANLIDWLGRKHGAISEIRNPGLVVKENDVRLSRVYPDPTTNATAPQDQNLV-TETLFAWIVPYVGIPAGGGVRAEQELAARYLVDNQRIMQLLLTNIFEMTSSFNKMVQVRFPETSTAQVHLDFTGLISLIDSLM : 1349
QIM07948.1 : IIEWLRETQAANVNRANLIDWLGRKHGAISEIRNPGLVVKENDVRLSRVYPDPTTNATAPQDQNLV-TETLFAWIVPYVGIPAGGGVRAEQELAARYLVDNQRIMQLLLTNIFEMTSSFNKMVQVRFPETSTAQVHLDFTGLISLIDSLM : 1349
QIM08183.1 : IIEWLRETQAANVNRANLIDWLGRKHGAISEIRNPGLVVKENDVRLSRVYPDPTTNATAPQDQNLV-TETLFAWIVPYVGIPAGGGVRAEQELAARYLVDNQRIMQLLLTNIFEMTSSFNKMVQVRFPETSTAQVHLDFTGLISLIDSLM : 1349
YP_0097024 : IIEWLRETQAANVNRANLIDWLGRKHGAISEIRNPGLVVKENDVRLSRVYPDPTTNATAPQDQNLV-TETLFAWIVPYVGIPAGGGVRAEQELAARYLVDNQRIMQLLLTNIFEMTSSFNKMVQVRFPETSTAQVHLDFTGLISLIDSLM : 1349
YP_0097038 : IIEWLRETQAANVNRANLIDWLGRKHGAISEIRNPGLVVKENDVRLSRVYPDPTTNATAPQDQNLV-TETLFAWIVPYVGIPAGGGVRAEQELAARYLVDNQRIMQLLLTNIFEMTSSFNKMVQVRFPETSTAQVHLDFTGLISLIDSLM : 1349
YP_0097023 : IIEWLRETQAANVNRANLIDWLGRKHGAISEIRNPGLVVKENDVRLSRVYPDPTTNATAPQDQNLV-TETLFAWIVPYVGIPAGGGVRAEQELAARYLVDNQRIMQLLLTNIFEMTSSFNKMVQVRFPETSTAQVHLDFTGLISLIDSLM : 1349
YP_0097026 : IIEWLRETQAANVNRANLIDWLGRKHGAISEIRNPGLVVKENDVRLSRVYPDPTTNATAPQDQNLV-TETLFAWIVPYVGIPAGGGVRAEQELAARYLVDNQRIMQLLLTNIFEMTSSFNKMVQVRFPETSTAQVHLDFTGLISLIDSLM : 1349
YP_0097035 : IIEWLRETQAANVNRANLIDWLGRKHGAISEIRNPGLVVKENDVRLSRVYPDPTTNATAPQDQNLV-TETLFAWIVPYVGIPAGGGVRAEQELAARYLVDNQRIMQLLLTNIFEMTSSFNKMVQVRFPETSTAQVHLDFTGLISLIDSLM : 1349
```

```
QZK26792.1 : IIEWLRETQAANVNRANLIDWLGRKHGAISEIRNPGLVIKEINMRLSMVYPDPTTEAATVQDRNLT-TETLFVWIVPYVGIPAGGGVRPEQELAARYLVDNQRIMQLLLTNIFEMTSSFNKMVQVRFPETSTAHVHLDFTGLISLIDSLM : 1349
YP_0097036 : IIEWLRETQAANVNRANLIDWLGRKHGAISEIRNPGLVVKENDVRLSRVYPDPTTNATAPQDQNLV-TETLFAWIVPYVGIPAGGGVRAEQELAARYLVDNQRIMQLLLTNIFEMTSSFNKMVQVRFPETSTAQVHLDFTGLISLIDSLM : 1349
QID21266.1 : IIEWLRETQAANVNRANLIDWLGRKHGAISEIRNPGLVVKENDARLSRVYPDPTTNATAPQDQNLV-TETLFAWIVPYVGIPAGGGVRAEQELAARYLVDNQRIMQLLLTNIFEMTSSFNKMVQVRFPETSTAQVHLDFTGLISLIDSLM : 1349
P0CA04.1   : IIEWLRETQAANVNHANLIDWLGRKHGAISEIRNPGLVVKENDARLSRVYPDPTTN-ATAPQDQNLVTETLFAWIVPYVGIPAGGGVRAEQELAARYLVDNQRIMQLLLTNIFEMTSSFNKMVQVRFPETSTAQVHLDFTGLISLIDSLM : 1349
P0CA02.1   : IIEWLRETQPPDVNHANLLDWLGRKHGAISEIRNPGLVVKANNARLSEVYPDPTTD-ANTPQDRNLTTETLFAWIVQYVGIPAGGGVRPEQELAARYLVDNQRIMQLLLTNIFEITSSFNKMIQVRFPETSTAHVHLDFTGLISLIDSLM : 1349
YP_0097029 : IVEWLRETQAANVNHANLIDWLGRRHGDISEIRNPGLVIKANDARLSMVYPDPTTDPTLPQDQNLT-TETLFAWIEQYVGIPAGGGIRPEQELAARYLVDNQRIMQLLLTNIFEMTSSFNKLVQVRFPETSTAHVHLDFTGLISLIDSLM : 1349
AXB50010.1 : IVEWLRETQAANVNHANLIDWLGRRHGDISEIRNPGLVIKANDARLSMVYPDPTTDPTLPQDQNLT-TETLFAWIEQYVGIPAGGGIRPEQELAARYLVDNQRIMQLLLTNIFEMTSSFNKLVQVRFPETSTAHVHLDFTGLISLIDSLM : 1349
AXB49666.1 : IVEWLRETQAANVNHANLIDWLGRRHGDISEIRNPGLVIKANDARLSMVYPDPTTDPTLPQDQNLT-TETLFAWIEQYVGIPAGGGIRPEQELAARYLVDNQRIMQLLLTNIFEMTSSFNKLVQVRFPETSTAHVHLDFTGLISLIDSLM : 1349
CAD7112308 : IIEWLRETQAANVNRANLIDWLGRRHGDISEIRNPGLVIKANDARLSEVYPDPTTDATAPLDRNLV-TETLFAWFTRFVGIPADGGVRPEQELAARYLVDNQRIMQLLLTNIFEMTSSFNKLVQVRFPETSTAHVHLDFTGLISLIDSLM : 1349
QRY19118.1 : IIEWLRETQAANVNRANLIDWLGRRHGDISEIRNPGLVIKANDARLSEVYPDPTTDATAPLDRNLV-TETLFAWFTRFVGIPADGGVRPEQELAARYLVDNQRIMQLLLTNIFEMTSSFNKLVQVRFPETSTAHVHLDFTGLISLIDSLM : 1349
P0CA01.1   : IIEWLRETQAANVNRANLIDWLGRRHGDISEIRNPGLVIKANDARLSEVYPDPTTDATAPLDRNLV-TETLFAWFTRFVGIPADGAVRPEQELAARYLVDNQRIMQLLLTNIFEMTSSFNKLVQVRFPETSTAHVHLDFTGLISLIDSLM : 1349
YP_0097028 : IIEWLRETQAANVNRANLIDWLGRRHGDISEIRNPGLVIKANDARLSEVYPDPTTDATAPLDRNLV-TETLFAWFTRFVGIPADGGVRPEQELAARYLVDNQRIMQLLLTNIFEMTSSFNKLVQVRFPETSTAHVHLDFTGLISLIDSLM : 1349
P0CA03.1   : IIEWLRET-PANVNHANLIDWLGRKHGAISEIRNPGLVIKEINMRLSEVYPDPTTE-ANVPQDRNLTTETLFAWIVPYVGIPAGGGVRAEQELAARYLVDNQRIMQLLLTNIFEMTSSFNKMVQVRFPETSTAQVHLDFTGLISLIDSLM : 1348

                 1360         *        1380         *        1400         *        1420         *        1440         *        1460         *        1480         *        1500
YP_0099272 : ADTKYFLDLLRPHIDKNIIQYYENRSNPGSFYWLEEHLIDKLIKPPTDAGGRPLPGGELGLEGVNQIINKTYTLLTKPYNVLQLRGGAQRRDAANIQINNNPQSSERFEQYGRVFSRLVFYDALENNSGLRVEQVALGDFRLSNLIRTNN : 1500
AZP54139.1 : ADTKYFLDLLRPHIDKNIIQYYENRSNPGSFYWLEEHLIDKLIKPPTDAGGRPLPGGELGLEGVNQIINKTYTLLTKPYNVLQLRGGAQRRDAANIQINNNPQSSERFEQYGRVFSRLVFYDALENNSGLRVEQVALGDFRLSNLIRTNN : 1500
QIE06881.1 : ADTKYFLDLLRPHIDKNIIQYYENRSNPGSFYWLEEHLIDKLIKPPTDAGGRPLPGGELGLEGVNQIINKTYTLLTKPYNVLQLRGGAQRRDAANIQINNNPQSSERFEQYGRVFSRLVFYDALENNSGLRVEQVALGDFRLSNLIRTNN : 1500
QGJ83454.1 : ADTKYFLDLLRPHIDKNIIQYYENRSNPGSFYWLEEHLIDKLIKPPTDAGGRPLPGGELGLEGVNQIINKTYTLLTKPYNVLQLRGGAQRRDAANIQINNNPQSSERFEQYGRVFSRLVFYDALENNSGLRVEQVALGDFRLSNLIRTNN : 1500
AYW34063.1 : ADTKYFLDLLRPHIDKNIIQYYENRSNPGSFYWLEEHLIDKLIKPPTDAGGRPLPGGELGLEGVNQIINKTYTLLTKPYNVLQLRGGAQRRDAANIQINNNPQSSERFEQYGRVFSRLVFYDALENNSGLRVEQVALGDFRLSNLIRTNN : 1500
QLF78620.1 : ADTKYFLDLLRPHIDKNIIQYYENRSNPGSFYWLEEHLIDKLIKPPTDAGGRPLPGGELGLEGVNQIINKTYTLLTKPYNVLQLRGGAQRRDAANIQINNNPQSSERFEQYGRVFSRLVFYDALENNSGLRVEQVALGDFRLSNLIRTNN : 1500
QTP96400.1 : ADTKYFLDLLRPHIDKNIIQYYENRSNPGSFYWLEEHLIDKLIKPPTDAGGRPLPGGELGLEGVNQIINKTYTLLTKSYNVLQLRGGAQRRDAANIQINNNPQSSERFEQYGRVFSRLVFYDALENNSGLRVEQVALGDFRLSNLIRTNN : 1500
QBH90765.1 : ADTKYFLDLLRPHIDKNIIQYYENRSNPGSFYWLEEHLIDKLIKPPTDAGGRPLPGGELGLEGVNQIINKTYTLLTKPYNVLQLRGGAQRRDAANIQINNNPQSSERFEQYGRVFSRLVFYDALENNSGLRVEQVALGDFRLSNLIRTNN : 1500
QOY24398.1 : ADTKYFLDLLRPHIDKNIIQYYENRSNPGSFYWLEEHLIDKLIKPPTDAGGRPLPGGELGLEGVNQIINKTYTLLTKPYNVLQLRGGAQRRDAANIQINNNPQSSERFEQYGRVFSRLVFYDALENNSGLRVEQVALGDFRLSNLIRTNN : 1500
QSG73791.1 : ADTKYFLDLLRPHIDKNIIQYYENRSNPGSFYWLEEHLIDKLIKPPTDAGGRPLPGGELGLEGVNQIINKTYTLLTKPYNVLQLRGGAQRRDAANIQINNNPQSSERFEQYGRVFSRLVFYDALENNSGLRVEQVALGDFRLSNLIRTNN : 1500
QTE18752.1 : ADTKYFLDLLRPHIDKNIIQYYENRSNPGSFYWLEEHLIDKLIKPPTDAGGRPLPGGELGLEGVNQIINKTYTLLTKPYNVLQLRGGAQRRDAANIQINNNPQSSERFEQYGRVFSRLVFYDALENNSGLRVEQVALGDFRLSNLIRTNN : 1500
VFV48026.1 : ADTKYFLDLLRPHIDKNIIQYYENRSNPGSFYWLEEHLIDKLIKPPTDAGGRPLPGGELGLEGVNQIINKTYTLLTKPYNVLQLRGGAQRRDAANIQINNNPQSSERFEQYGRVFSRLVFYDALENNSGLRVEQVALGDFRLSNLIRTNN : 1500
QOW03225.1 : ADTKYFLDLLRPHIDKNIIQYYENRSNPGSFYWLEEHLIDKLIKPPTDAGGRPLPGGELGLEGVNQIINKTYTLLTKPYNVLQLRGGAQRRDAANIQINNNPQSSERFEQYGRVFSRLVFYDALENNSGLRVEQVALGDFRLSNLIRTNN : 1500
QOW03039.1 : ADTKYFLDLLRPHIDKNIIQYYENRSNPGSFYWLEEHLIDKLIKPPTDAGGRPLPGGELGLEGVNQIINKTYTLLTKPYNVLQLRGGAQRRDAANIQINNNPQSSERFEQYGRVFSRLVFYDALENNSGLRVEQVALGDFRLSNLIRTNN : 1500
VVW94185.1 : ADTKYFLDLLRPHIDKNIIQYYENRSNPGSFYWLEEHLIDKLIKPPTDAGGRPLPGGELGLEGVNQIINKTYTLLTKPYNVLQLRGGAQRRDAANIQINNNPQSSERFEQYGRVFSRLVFYDALENNSGLRVEQVALGDFRLSNLIRTNN : 1500
CAD0059543 : ADTKYFLDLLRPHIDKNIIQYYENRSNPGSFYWLEEHLIDKLIKPPTDAGGRPLPGGELGLEGVNQIINKTYTLLTKPYNVLQLRGGAQRRDAANIQINNNPQSSERFEQYGRVFSRLVFYDALENNSGLRVEQVALGDFRLSNLIRTNN : 1500
QXP50051.1 : ADTKYFLDLLRPHIDKNIIQYYENRSNPGSFYWLEEHLIDKLIKPPTDAGGRPLPGGELGLEGVNQIINKTYTLLTKPYNVLQLRGGAQRRDAANIQINNNPQSSERFEQYGRVFSRLVFYDALENNSGLRVEQVALGDFRLSNLIRTNN : 1500
CAD7112603 : ADTKYFLDLLRPHIDKNIIQYYENRSNPGSFYWLEEHLIDKLIKPPTDAGGRPLPGGELGLEGVNQIINKTYTLLTKPYNVLQLRGGAQRRDAANIQINNNPQSSERFEQYGRVFSRLVFYDALENNSGLRVEQVALGDFRLSNLIRTNN : 1500
AXZ96054.1 : ADTKYFLDLLRPHIDKNIIQYYENRSNPGSFYWLEEHLIDKLIKPPTDAGGRPLPGGELGLEGVNQIINKTYTLLTKPYNVLQLRGGAQRRDAANIQINNNPQSSERFEQYGRVFSRLVFYDALENNSGLRVEQVALGDFRLSNLIRTNN : 1500
QED90535.1 : ADTKYFLDLLRPHIDKNIIQYYENRSNPGSFYWLEEHLIDKLIKPPTDAGGRPLPGGELGLEGVNQIINKTYTLLTKPYNVLQLRGGAQRRDAANIQINNNPQSSERFEQYGRVFSRLVFYDALENNSGLRVEQVALGDFRLSNLIRTNN : 1500
VVW94190.1 : ADTKYFLDLLRPHIDKNIIQYYENRSNPGSFYWLEEHLIDKLIKPPTDAGGRPLPGGELGLEGVNQIINKTYTLLTKPYNVLQLRGGAQRRDAANIQINNNPQSSERFEQYGRVFSRLVFYDALENNSGLRVEQVALGDFRLSNLIRTNN : 1500
QEY87898.1 : ADTKYFLDLLRPHIDKNIIQYYENRSNPGSFYWLEEHLIDKLIKPPTDAGGRPLPGGELGLEGVNQIINKTYTLLTKPYNVLQLRGGAQRRDAANIQINNNPQSSERFEQYGRVFSRLVFYDALENNSGLRVEQVALGDFRLSNLIRTNN : 1500
QGV56805.1 : ADTKYFLDLLRPHIDKNIIQYYENRSNPGSFYWLEEHLIDKLIKPPTDAGGRPLPGGELGLEGVNQIINKTYTLLTKPYNVLQLRGGAQRRDAANIQINNNPQSSERFEQYGRVFSRLVFYDALENNSGLRVEQVALGDFRLSNLIRTNN : 1500
QPB67618.1 : ADTKYFLDLLRPHIDKNIIQYYENRSNPGSFYWLEEHLIDKLIKPPTDAGGRPLPGGELGLEGVNQIINKTYTLLTKPYNVLQLRGGAQRRDAANIQINNNPQSSERFEQYGRVFSRLVFYDALENNSGLRVEQVALGDFRLSNLIRTNN : 1500
QUQ60377.1 : ADTKYFLDLLRPHIDKNIIQYYENRSNPGSFYWLEEHLIDKLIKPPTDAGGRPLPGGELGLEGVNQIINKTYTLLTKPYNVLQLRGGAQRRDAANIQINNNPQSSERFEQYGRVFSRLVFYDALENNSGLRVEQVALGDFRLSNLIRTNN : 1500
QUQ60197.1 : ADTKYFLDLLRPHIDKNIIQYYENRSNPGSFYWLEEHLIDKLIKPPTDAGGRPLPGGELGLEGVNQIINKTYTLLTKPYNVLQLRGGAQRRDAANIQINNNPQSSERFEQYGRVFSRLVFYDALENNSGLRVEQVALGDFRLSNLIRTNN : 1500
SPS73515.1 : ADTKYFLDLLRPHIDKNIIQYYENRSNPGSFYWLEEHLIDKLIKPPTDAGGRPLPGGELGLEGVNQIINKTYTLLTKPYNVLQLRGGAQRRDAANIQINNNPQSSERFEQYGRVFSRLVFYDALENNSGLRVEQVALGDFRLSNLIRTNN : 1500
QDL88123.1 : ADTKYFLDLLRPHIDKNIIQYYENRSNPGSFYWLEEHLIDKLIKPPTDAGGRPLPGGELGLEGVNQIINKTYTLLTKPYNVLQLRGGAQRRDAANIQINNNPQSSERFEQYGRVFSRLVFYDALENNSGLRVEQVALGDFRLSNLIRTNN : 1500
QIA61506.1 : ADTKYFLDLLRPHIDKNIIQYYENRSNPGSFYWLEEHLIDKLIKPPTDAGGRPLPGGELGLEGVNQIINKTYTLLTKPYNVLQLRGGAQRRDAANIQINNNPQSSERFEQYGRVFSRLVFYDALENNSGLRVEQVALGDFRLSNLIRTNN : 1500
QTZ19752.1 : ADTKYFLDLLRPHIDKNIIQYYENRSNPGSFYWLEEHLIDKLIKPPTDAGGRPLPGGELGLEGVNQIINKTYTLLTKPYNVLQLRGGAQRRDAANIQINNNPQSSERFEQYGRVFSRLVFYDALENNSGLRVEQVALGDFRLSNLIRTNN : 1500
QIM07715.1 : ADTKYFLNLLRPHIDKNIIQYYENRSNPGSFYWLEEHLIDKLIKPPTDAGGRPLPGGELGLEGVNQIINKTYILLTKPYNVLQLRGGVQRRDAANIQINNNPQPSERFEQYGRVFSRLVFYDALENNSGLRVEQVVLGDFRLSNLIRTNN : 1499
QIM07948.1 : ADTKYFLNLLRPHIDKNIIQYYENRSNPGSFYWLEEHLIDKLIKPPTDAGGRPLPGGELGLEGVNQIINKTYILLTKPYNVLQLRGGVQRRDAANIQINNNPQPSERFEQYGRVFSRLVFYDALENNSGLRVEQVVLGDFRLSNLIRTNN : 1499
QIM08183.1 : ADTKYFLNLLRPHIDKNIIQYYENRSNPGSFYWLEEHLIDKLIKPPTDAGGRPLPGGELGLEGVNQIINKTYILLTKPYNVLQLRGGVQRRDAANIQINNNPQPSERFEQYGRVFSRLVFYDALENNSGLRVEQVVLGDFRLSNLIRTNN : 1499
YP_0097024 : ADTKYFLNLLRPHIDKNIIQYYENRSNPGSFYWLEEHLIDKLIKPPTDAGGRPLPGGELGLEGVNQIINKTYILLTKPYNVLQLRGGVQRRDAANIQINNNPQPSERFEQYGRVFSRLVFYDALENNSGLRVEQVVLGDFRLSNLIRTNN : 1499
YP_0097038 : ADTKYFLNLLRPHIDKNIIQYYENRSNPGSFYWLEEHLIDKLIKPPTDAGGRPLPGGELGLEGVNQIINKTYILLTKPYNVLQLRGGVQRRDAANIQINNNPQPSERFEQYGRVFSRLVFYDALENNSGLRVEQVVLGDFRLSNLIRTNN : 1499
YP_0097023 : ADTKYFLNLLRPHIDKNIIQYYENRSNPGSFYWLEEHLIDKLIKPPTDAGGRPLPGGELGLEGVNQIINKTYILLTKPYNVLQLRGGVQRRDAANIQINNNPQPSERFEQYGRVFSRLVFYDALENNSGLRVEQVVLGDFRLSNLIRTNN : 1499
YP_0097026 : ADTKYFLNLLRPHIDKNIIQYYENRSNPGSFYWLEEHLIDKLIKPPTDAGGRPLPGGELGLEGVNQIINKTYILLTKPYNVLQLRGGVQRRDAANIQINNNPQPSERFEQYGRVFSRLVFYDALENNSGLRVEQVVLGDFRLSNLIRTNN : 1499
YP_0097035 : ADTKYFLNLLRPHIDKNIIQYYENRSNPGSFYWLEEHLIDKLIKPPTDAGGRPLPGGELGLEGVNQIINKTYILLTKPYNVLQLRGGVQRRDAANIQINNNPQPSERFEQYGRVFSRLVFYDALENNSGLRVEQVVLGDFRLSNLIRTNN : 1499
QZK26792.1 : ADTKYFLDLLRPHIDKNIIQYYENRSNPGSFYWLEEHLIDKLIKPPTDAGGRPLPGGELGLEGVNQIINKTYTLLTKPYNVLQLRGGAQRRDAANIQINNNPQSSERFEQYGRVFSRLVFYDALENNSGLRVEQVALGDFRLSNLIRTNN : 1499
YP_0097036 : ADTKYFLNLLRPHIDKNIIQYYENRSNPGSFYWLEEHLIDKLIKPPTDAGGRPLPGGELGLEGVNQIINKTYILLTKPYNVLQLRGGVQRRDAANIQINNNPQPSERFEQYGRVFSRLVFYDALENNSGLRVEQVVLGDFRLSNLIRTNN : 1499
QID21266.1 : ADTKYFLNLLRPHIDKNIIQYYENRSNPGSFYWLEEHLIDKLIKPPTDAGGRPLPGGELGLEGVNQIINKTYTLLTKPYNVLQLQGGAQRRDAANIQINNNPQPSERFEQYGRVFSRLVFYDALENNSGLRVEQVVLGDFRLSNLIRTNN : 1499
P0CA04.1   : ADTKYFLNLLRPHIDKNIIQYYENRSNPGSFYWLEEHLIDKLIKPPTDAGGRPLPGGELGLEGVNQIINKTYTLLTKPYNVLQLQGGAQRRDAANIQINNNPQPSERFEQYGRVFSRLVFYDALENNSGLRVEQVVLGDFRLSNLIRTNN : 1499
P0CA02.1   : ADTKYFLDLLRPHIDKNIIQYYENRSNPGSFYWLEEHLIDKLIKPPTDARGRPLPGGELGLEGVNQIINKTYTLLTKPYNVLQLRGGAQRRDAANIQINNNPQSSERFEQYGRVFSKLVFYDALENNSGLRVEQVALGDFRLSNLIRTNN : 1499
YP_0097029 : ADTKYFLDLLRPHIDKNIIQYYENRSNPGSFYWLEEHLIDKLIKPPTDAGGRPLPGGELGLEGVNQIINKTYILLTKPYNVLHLRGGAQRRDAANIQINNNPELSERYEQYGRVFSRLVFYDALDNNSGLRVEQVALGDFRLSNLIRTNN : 1499
AXB50010.1 : ADTKYFLDLLRPHIDKNIIQYYENRSNPGSFYWLEEHLIDKLIKPPTDAGGRPLPGGELGLEGVNQIINKTYILLTKPYNVLHLRGGAQRRDAANIQINNNPELSERYEQYGRVFSRLVFYDALDNNSGLRVEQVALGDFRLSNLIRTNN : 1499
AXB49666.1 : ADTKYFLDLLRPHIDKNIIQYYENRSNPGSFYWLEEHLIDKLIKPPTDAGGRPLPGGELGLEGVNQIINKTYILLTKPYNVLHLRGGAQRRDAANIQINNNPELSERYEQYGRVFSRLVFYDALDNNSGLRVEQVALGDFRLSNLIRTNN : 1499
CAD7112308 : ADTKYFLDLLRPHIDKNIIQYYENRSNPGSFYWLEEHLIDKLIKPPTDAGGRPLPGGELGLEGVNQIINKTYILLTKPYNVLQLRGGAQRGNAANIQINNNPELSERYEQYGRVFSRLVFYDALIENSGLRVEQVALGDFRLSNLVRTNN : 1499
QRY19118.1 : ADTKYFLDLLRPHIDKNIIQYYENRSNPGSFYWLEEHLIDKLIKPPTDAGGRPLPGGELGLEGVNQIINKTYILLTKPYNVLQLRGGAQRGNAANIQINNNPEHSERYEQYGRVFSRLVFYDALIENSGLRVEQVALGDFRLSNLVRTNN : 1499
P0CA01.1   : ADTKYFLDLLRPHIDKNIIQYYENRSNPGSFYWLEEHLIDKLIKPPTDAGGRPLPGGELGLEGVNQIINKTYILLTKPYNVLQLRGGAQRGNAANIQINNNPEFSERYEQYGRVFSRLVFYDALIENSGLRVEQVALGDFRLSNLIRTNN : 1499
YP_0097028 : ADTKYFLDLLRPHIDKNIIQYYENRSNPGSFYWLEEHLIDKLIKPPTDAGGRPLPGGELGLEGVNQIINKTYILLTKPYNVLQLRGGAQRGNAANIQINNNPELSERYEQYGRVFSRLVFYDALIENSGLRVEQVALGDFRLSNLVRTNN : 1499
P0CA03.1   : ADTKYFLNLLRPHIDKNIIQYYENRSNPGSFYWLEEHLIDKLIKPPTDAGGRPLPGGELGLEGVNQIINKTYTLLTKPYNVLQLRGGAQRRDAANIQINNNPQPSERFEQYGRVFSRLVFYDALENNSGLRVEQVVLGDFRLSNLIRTNN : 1498
```

```
                          *      1520         *       1540         *       1560         *       1580         *       1600         *       1620         *       1640         *
YP_0099272 : AQEENTLSYWDNIA--LRTYANVNDAANNLRRYRLYGSDYGIQNNRSMMMVFNQLIASYITRFYDAPSGKIYLNLINAFANGNFSQAVMEMGYAHPDLARNNNVFGHRGDPTEQSVLLLSLGLILQRLIKDTNRQGLSQHLISTLTEIPI : 1648
AZP54139.1 : AQEENTLSYWDNIA--LRTYANVNDAANNLRRYRLYGSDYGIQNNRSMMMVFNQLIASYITRFYDAPSGKIYLNLINAFANGNFSQAVMEMGYAHPDLARNNNVFGHRGDPTEQSVLLLSLGLILQRLIKDTNRQGLSQHLISTLTEIPI : 1648
QIE06881.1 : AQEENTLSYWDNIA--LRTYANVNDAANNLRRYRLYGSDYGIQNNRSMMMVFNQLIASYITRFYDAPSGKIYLNLINAFANGNFSQAVMEMGYAHPDLARNNNVFGHRGDPTEQSVLLLSLGLILQRLIKDTNRQGLSQHLISTLTEIPI : 1648
QGJ83454.1 : AQEENTLSYWDNIA--LRTYANVNDAANNLRRYRLYGSDYGIQNNRSMMMVFNQLIASYITRFYDAPSGKIYLNLINAFANGNFSQAVMEMGYAHPDLARNNNVFGHRGDPTEQSVLLLSLGLILQRLIKDTNRQGLSQHLISTLTEIPI : 1648
AYW34063.1 : AQEENTLSYWDNIA--LRTYANVNDAANNLRRYRLYGSDYGIQNNRSMMMVFNQLIASYITRFYDAPSGKIYLNLINAFANGNFSQAVMEMGYAHPDLARNNNVFGHRGDPTEQSVLLLSLGLILQRLIKDTNRQGLSQHLISTLTEIPI : 1648
QLF78620.1 : AQEENTLSYWDNIA--LRTYANVNDAANNLRRYRLYGSDYGIQNNRSMMMVFNQLIASYITRFYDAPSGKIYLNLINAFANGNFSQAVMEMGYAHPDLARNNNVFGHRGDPTEQSVLLLSLGLILQRLIKDTNRQGLSQHLISTLTEIPI : 1648
QTP96400.1 : AQEENTLSYWDNIA--LRTYANVNDAANNLRRYRLYGSDYGIQNNRSMMMVFNQLIASYITRFYDAPSGKIYLNLINAFANGNFSQAVMEMGYAHPDLARNNNVFGHRGDPTEQSVLLLSLGLILQRLIKDTNRQGLSQHLISTLTEIPV : 1648
QBH90765.1 : AQEENTLSYWDNIA--LRTYANVNDAANNLRRYRLYGSDYGIQNNRSMMMVFNQLIASYITRFYDAPSGKIYLNLINAFANGNFSQAVMEMGYAHPDLARNNNVFGHRGDPTEQSVLLLSLGLILQRLIKDTNRQGLSQHLISTLTEIPI : 1648
QOY24398.1 : AQEENTLSYWDNIA--LRTYANVNDAANNLRRYRLYGSDYGIQNNRSMMMVFNQLIASYITRFYDAPSGKIYLNLINAFANGNFSQAVMEMGYAHPDLARNNNVFGHRGDPTEQSVLLLSLGLILQRLIKDTNRQGLSQHLISTLTEIPI : 1648
QSG73791.1 : AQEENTLSYWDNIA--LRTYANVNDAANNLRRYRLYGSDYGIQNNRSMMMVFNQLIASYITRFYDAPSGKIYLNLINAFANGNFSQAVMEMGYAHPDLARNNNVFGHRGDPTEQSVLLLSLGLILQRLIKDTNRQGLSQHLISTLTEIPI : 1648
QTE18752.1 : AQEENTLSYWDNIA--LRTYANVNDAANNLRRYRLYGSDYGIQNNRSMMMVFNQLIASYITRFYDAPSGKIYLNLINAFANGNFSQAVMEMGYAHPDLARNNNVFGHRGDPTEQSVLLLSLGLILQRLIKDTNRQGLSQHLISTLTEIPI : 1648
VFV48026.1 : AQEENTLSYWDNIA--LRTYANVNDAANNLRRYRLYGSDYGIQNNRSMMMVFNQLIASYITRFYDAPSGKIYLNLINAFANGNFSQAVMEMGYAHPDLARNNNVFGHRGDPTEQSVLLLSLGLILQRLIKDTNRQGLSQHLISTLTEIPI : 1648
QOW03225.1 : AQEENTLSYWDNIA--LRTYANVNDAANNLRRYRLYGSDYGIQNNRSMMMVFNQLIASYITRFYDAPSGKIYLNLINAFANGNFSQAVMEMGYAHPDLARNNNVFGHRGDPTEQSVLLLSLGLILQRLIKDTNRQGLSQHLISTLTEIPI : 1648
QOW03039.1 : AQEENTLSYWDNIA--LRTYANVNDAANNLRRYRLYGSDYGIQNNRSMMMVFNQLIASYITRFYDAPSGKIYLNLINAFANGNFSQAVMEMGYAHPDLARNNNVFGHRGDPTEQSVLLLSLGLILQRLIKDTNRQGLSQHLISTLTEIPI : 1648
VVW94185.1 : AQEENTLSYWDNIA--LRTYANVNDAANNLRRYRLYGSDYGIQNNRSMMMVFNQLIASYITRFYDAPSGKIYLNLINAFANGNFSQAVMEMGYAHPDLARNNNVFGHRGDPTEQSVLLLSLGLILQRLIKDTNRQGLSQHLISTLTEIPI : 1648
CAD0059543 : AQEENTLSYWDNIA--LRTYANVNDAANNLRRYRLYGSDYGIQNNRSMMMVFNQLIASYITRFYDAPSGKIYLNLINAFANGNFSQAVMEMGYAHPDLARNNNVFGHRGDPTEQSVLLLSLGLILQRLIKDTNRQGLSQHLISTLTEIPI : 1648
QXP50051.1 : AQEENTLSYWDNIA--LRTYANVNDAANNLRRYRLYGSDYGIQNNRSMMMVFNQLIASYITRFYDAPSGKIYLNLINAFANGNFSQAVMEMGYAHPDLARNNNVFGHRGDPTEQSVLLLSLGLILQRLIKDTNRQGLSQHLISTLTEIPI : 1648
CAD7112603 : AQEENTLSYWDNIA--LRTYANVNDAANNLRRYRLYGSDYGIQNNRSMMMVFNQLIASYITRFYDAPSGKIYLNLINAFANGNFSQAVMEMGYAHPDLARNNNVFGHRGDPTEQSVLLLSLGLILQRLIKDTNRQGLSQHLISTLTEIPI : 1648
AXZ96054.1 : AQEENTLSYWDNIA--LRTYANVNDAANNLRRYRLYGSDYGIQNNRSMMMVFNQLIASYITRFYDAPSGKIYLNLINAFANGNFSQAVMEMGYAHPDLARNNNVFGHRGDPTEQSVLLLSLGLILQRLIKDTNRQGLSQHLISTLTEIPI : 1648
QED90535.1 : AQEENTLSYWDNIA--LRTYANVNDAANNLRRYRLYGSDYGIQNNRSMMMVFNQLIASYITRFYDAPSGKIYLNLINAFANGNFSQAVMEMGYAHPDLARNNNVFGHRGDPTEQSVLLLSLGLILQRLIKDTNRQGLSQHLISTLTEIPI : 1648
VVW94190.1 : AQEENTLSYWDNIA--LRTYANVNDAANNLRRYRLYGSDYGIQNNRSMMMVFNQLIASYITRFYDAPSGKIYLNLINAFANGNFSQAVMEMGYAHPDLARNNNVFGHRGDPTEQSVLLLSLGLILQRLIKDTNRQGLSQHLISTLTEIPI : 1648
QEY87898.1 : AQEENTLSYWDNIA--LRTYANVNDAANNLRRYRLYGSDYGIQNNRSMMMVFNQLIASYITRFYDAPSGKIYLNLINAFANGNFSQAVMEMGYAHPDLARNNNVFGHRGDPTEQSVLLLSLGLILQRLIKDTNRQGLSQHLISTLTEIPI : 1648
QGV56805.1 : AQEENTLSYWDNIA--LRTYANVNDAANNLRRYRLYGSDYGIQNNRSMMMVFNQLIASYITRFYDAPSGKIYLNLINAFANGNFSQAVMEMGYAHPDLARNNNVFGHRGDPTEQSVLLLSLGLILQRLIKDTNRQGLSQHLISTLTEIPI : 1648
QPB67618.1 : AQEENTLSYWDNIA--LRTYANVNDAANNLRRYRLYGSDYGIQNNRSMMMVFNQLIASYITRFYDAPSGKIYLNLINAFANGNFSQAVMEMGYAHPDLARNNNVFGHRGDPTEQSVLLLSLGLILQRLIKDTNRQGLSQHLISTLTEIPI : 1648
QUQ60377.1 : AQEENTLSYWDNIA--LRTYANVNDAANNLRRYRLYGSDYGIQNNRSMMMVFNQLIASYITRFYDAPSGKIYLNLINAFANGNFSQAVMEMGYAHPDLARNNNVFGHRGDPTEQSVLLLSLGLILQRLIKDTNRQGLSQHLISTLTEIPI : 1648
QUQ60197.1 : AQEENTLSYWDNIA--LRTYANVNDAANNLRRYRLYGSDYGIQNNRSMMMVFNQLIASYITRFYDAPSGKIYLNLINAFANGNFSQAVMEMGYAHPDLARNNNVFGHRGDPTEQSVLLLSLGLILQRLIKDTNRQGLSQHLISTLTEIPI : 1648
SPS73515.1 : AQEENTLSYWDNIA--LRTYANVNDAANNLRRYRLYGSDYGIQNNRSMMMVFNQLIASYITRFYDAPSGKIYLNLINAFANGNFSQAVMEMGYAHPDLARNNNVFGHRGDPTEQSVLLLSLGLILQRLIKDTNRQGLSQHLISTLTEIPI : 1648
QDL88123.1 : AQEENTLSYWDNIA--LRTYANVNDAANNLRRYRLYGSDYGIQNNRSMMMVFNQLIASYITRFYDAPSGKIYLNLINAFANGNFSQAVMEMGYAHPDLARNNNVFGHRGDPTEQSVLLLSLGLILQRLIKDTNRQGLSQHLISTLTEIPI : 1648
QIA61506.1 : AQEENTLSYWDNIA--LRTYANVNDAANNLRRYRLYGSDYGIQNNRSMMMVFNQLIASYITRFYDAPSGKIYLNLINAFANGNFSQAVMEMGYAHPDLARNNNVFGHRGDPTEQSVLLLSLGLILQRLIKDTNRQGLSQHLISTLTEIPI : 1648
QTZ19752.1 : AQEENTLSYWDNIA--LRTYANVNDAANNLRRYRLYGSDYGIQNNRSMMMVFNQLIASYITRFYDAPSGKIYLNLINAFANGNFSQAVMEMGYAHPDLARNNNVFGHRGDPTEQSVLLLSLGLILQRLIKDTNRQGLSQHLISTLTEIPI : 1648
QIM07715.1 : AQEENTLSYWDNMA--PRTYANVNDAANNLRRYRLYGSDYGIQNNRSMMMVFNQLVASYIARFYDAPSGKIYLNLINAFANGNFSQAVMELGYTHPDLARDNIAFGHRGDPTEQSVLLLSLGLMLQRLIKDTNRQGLSQHLISTLTEIPI : 1647
QIM07948.1 : AQEENTLSYWDNMA--PRTYANVNDAANNLRRYRLYGSDYGIQNNRSMMMVFNQLVASYIARFYDAPSGKIYLNLINAFANGNFSQAVMELGYTHPDLARDNIAFGHRGDPTEQSVLLLSLGLMLQRLIKDTNRQGLSQHLISTLTEIPI : 1647
QIM08183.1 : AQEENTLSYWDNMA--PRTYANVNDAANNLRRYRLYGSDYGIQNNRSMMMVFNQLVASYIARFYDAPSGKIYLNLINAFANGNFSQAVMELGYTHPDLARDNIAFGHRGDPTEQSVLLLSLGLMLQRLIKDTNRQGLSQHLISTLTEIPI : 1647
YP_0097024 : AQEENTLSYWDNMA--PRTYANVNDAANNLRRYRLYGSDYGIQNNRSMMMVFNQLVASYIARFYDAPSGKIYLNLINAFANGNFSQAVMELGYTHPDLARDNIAFGHRGDPTEQSVLLLSLGLMLQRLIKDTNRQGLSQHLISTLTEIPI : 1647
YP_0097038 : AQEENTLSYWDNMA--PRTYANVNDAANNLRRYRLYGSDYGIQNNRSMMMVFNQLVASYIARFYDAPSGKIYLNLINAFANGNFSQAVMELGYTHPDLARDNIAFGHRGDPTEQSVLLLSLGLMLQRLIKDTNRQGLSQHLISTLTEIPI : 1647
YP_0097023 : AQEENTLSYWDNMA--PRTYANVNDAANNLRRYRLYGSDYGIQNNRSMMMVFNQLVASYIARFYDAPSGKIYLNLINAFANGNFSQAVMELGYTHPDLARDNIAFGHRGDPTEQSVLLLSLGLMLQRLIKDTNRQGLSQHLISTLTEIPI : 1647
YP_0097026 : AQEENTLSYWDNMA--PRTYANVNDAANNLRRYRLYGSDYGIQNNRSMMMVFNQLVASYIARFYDAPSGKIYLNLINAFANGNFSQAVMELGYTHPDLARDNIAFGHRGDPTEQSVLLLSLGLMLQRLIKDTNRQGLSQHLISTLTEIPI : 1647
YP_0097035 : AQEENTLSYWDNMA--PRTYANVNDAANNLRRYRLYGSDYGIQNNRSMMMVFNQLVASYIARFYDAPSGKIYLNLINAFANGNFSQAVMELGYTHPDLARDNIAFGHRGDPTEQSVLLLSLGLMLQRLIKDTNRQGLSQHLISTLTEIPI : 1647
QZK26792.1 : AQEENALSYWDNIA--LRTYANVNDAANNLRRYRLYGSDYGIRNNRSMMMVFNQLVASYIARFYDAPSGKIYLNLINAFANGNFSQAVMELGYTHPDLARDNIAFGHRGDPTEQSVLLLSLGLMLQRLIKDTNRQGLSQHLISTLTEIPI : 1647
YP_0097036 : AQEENTLSYWDNMA--PRTYANVNDAANNLRRYRLYGSDYGIQNNRSMMMVFNQLVASYIARFYDAPSGKIYLNLINAFANGNFSQAVMELGYTHPDLARDNIAFGHRGDPTEQSVLLLSLGLMLQRLIKDTNRQGLSQHLISTLTEIPI : 1647
QID21266.1 : AQEENALSYWDNIA--LRTYANVNDAANNLRRYRLYGSDQGIQNNRSMMMVFNQLVASYIARFYDAPSGKIYLNLINAFANGNFSQAVMEMGYAHPDLARNNNAFGHRGDPTEQSVLLLSLGLILQRLIKDTNRQGLSQHLISTLTEIPI : 1647
P0CA04.1   : AQEENALSYWDNIA--LRTYANVNDAANNLRRYRLYGSDHGIQNNRSMMMVFNQLVASYIARFYDAPSGKIYLNLINAFANGNFSQAVMEMGYAHPDLARNNNAFGHRGDPTEQSVLLLSLGLILQRLIKDTNRQGLSQHLISTLTEIPI : 1647
P0CA02.1   : AQEENTLSYWDNIA--LRTYANVNDAANNLRRYRLYGSDYGIQNNRSMMMVFNQLIASYITRFYDAPSGKIYLNLINAFANGNFSQAVMEMGYAHPDLARNNNAFGHRGDPTEQSVLLLSLGLILQRLIKDTNRQGLSQHLISTLTEIPI : 1647
YP_0097029 : AQEENALSFWTAVAPAPRAYANVNNAANN-RRYRLYGSDYGIRNNRSMMMVFNQLVASYIARFYDAPSGKIYLNLINAFANGNFSQAVMELGYAHPDLARDNIAFGHRGDPTEQSVLLLSLGLMLQRLIKDTNRQGLSQHLISTLTEIPI : 1648
AXB50010.1 : AQEENALSFWTAVAPAPRAYANVNNAANNLRRYRLYGSDYGIRNNRSMMMVFNQLVASYIARFYDAPSGKIYLNLINAFANGNFSQAVMELGYAHPDLARDNIAFGHRGDPTEQSVLLLSLGLMLQRLIKDTNRQGLSQHLISTLTEIPI : 1649
AXB49666.1 : AQEENALSFWTAVAPAPRAYANVNNAANNLRRYRLYGSDYGIRNNRSMMMVFNQLVASYIARFYDAPSGKIYLNLINAFANGNFSQAVMELGYAHPDLARDNIAFGHRGDPTEQSVLLLSLGLMLQRLIKDTNRQGLSQHLISTLTEIPI : 1649
CAD7112308 : AQEENALSFWTAVA--PRAYANVNDAANNLRRYRLYGSDYGIRNNRSMMMVFNQLVASYIARFYDAPSGKIYLNLINAFANGNFSQAVMELGYAHPDLARDNTAFGHRGDPTEQSVLLLSLGLMLQRLIKDTNRQGLSQHLISTLTEIPI : 1647
QRY19118.1 : AQEENALSFWTAVA--PRAYANVNDAANNLRRYRLYGSDYGIRNNRSMMMVFNQLVASYIARFYDAPSGKIYLNLINTFANGNFSQAVMELGYAHPDLARDNTAFGHRGDPTEQSVLLLSLGLMLQRLIKDTNRQGLSQHLISTLTEIPI : 1647
P0CA01.1   : AQEENALSFWTAVA--PRAYANVNDAANNLRRYRLYGSDYGIRNNRSMMMVFNQLVASYIARFYDAPSGKIYLNLINTFANGNFSQAVMELGYAHPDLARDNTAFGHRGDPTEQSVLLLSLGLMLQRLIKDTNRQGLSQHLISTLTEIPI : 1647
YP_0097028 : AQEENALSFWTAVA--PRAYANVNDAANNLRRYRLYGSDYGIRNNRSMMMVFNQLVASYIARFYDAPSGKIYLNLINAFANGNFSQAVMELGYAHPDLARDNTAFGHRGDPTEQSVLLLSLGLMLQRLIKDTNRQGLSQHLISTLTEIPI : 1647
P0CA03.1   : AQEENALSYWDNIA--LRTYANVNDAANNLRRYRLYGSDHGIQNNRSMMMVFNQLVASYIARFYDAPSGKIYLNLINAFANGNFSQAVMEMGYAHPDLARNNNAFGHRGDPTEQSVLLLSLGLILQRLIKDTNRQGLSQHLISTLTEIPI : 1646
                                                                                   f

                  1660         *       1680         *       1700         *       1720         *       1740         *       1760         *       1780         *       1800
YP_0099272 : YLKENYRANLPLFNKMFNILISQGELLKQFIQYTNVQLARPNLTALLGANNDSVIYYNNN-NVPATGLSVGQAALRGIGGVFRPNVTLMPLGDAQNNTSDVVRKRLVAVIDGIIRGSHTLADSAMEVLHELTDHPIYLETEEHFIQNYMS : 1797
AZP54139.1 : YLKENYRANLPLFNKMFNILISQGELLKQFIQYTNVQLARPNLTALLGANNDSVIYYNNN-NVPATGLSVGQAALRGIGGVFRPNVTLMPLGDAQNNTSDVVRKRLVAVIDGIIRGSHTLADSAMEVLHELTDHPIYLETEEHFIQNYMS : 1797
QIE06881.1 : YLKENYRANLPLFNKMFNILISQGELLKQFIQYTNVQLARPNLTALLGANNDSVIYYNNN-NVPATGLSVGQAALRGIGGVFRPNVTLMPLGDAQNNTSDVVRKRLVAVIDGIIRGSHTLADSAMEVLHELTDHPIYLETEEHFIQNYMS : 1797
QGJ83454.1 : YLKENYRANLPLFNKMFNILISQGELLKQFIQYTNVQLARPNLTALLGANNDSVIYYNNN-NVPATGLSVGQAALRGIGGVFRPNVTLMPLGDAQNNTSDVVRKRLVAVIDGIIRGSHTLADSAMEVLHELTDHPIYLETEEHFIQNYMS : 1797
AYW34063.1 : YLKENYRANLPLFNKMFNILISQGELLKQFIQYTNVQLARPNLTALLGANNDSVIYYNNN-NVPATGLSVGQAALRGIGGVFRPNVTLMPLGDAQNNTSDVVRKRLVAVIDGIIRGSHTLADSAMEVLHELTDHPIYLETEEHFIQNYMS : 1797
QLF78620.1 : YLKENYRANLPLFNKMFNILISQGELLKQFIQYTNVQLARPNLTALLGANNDSVIYYNNN-NVPATGLSVGQAALRGIGGVFRPNVTLMPLGDAQNNTSDVVRKRLVAVIDGIIRGSHTLADSAMEVLHELTDHPIYLETEEHFIQNYMS : 1797
QTP96400.1 : YLKENYRANLPLFNKMFNILISQGELLKQFIQYTNVQLARPNLTALLGANNDSVIYYNNN-NVPATGLSVGQAALRGIGGVFRPNVTLMPLGDAQNNTSDVVRKRLVAVIDGIIRGSHTLADSAMEVLHELTDHPIYLETEEHFIQNYMS : 1797
QBH90765.1 : YLKENYRANLPLFNKMFNILISQGELLKQFIQYTNVQLARPNLTALLGANNDSVIYYNNN-NVPATGLSVGQAALRGIGGVFRPNVTLMPLGDAQNNTSDVVRKRLVAVIDGIIRGSHTLADSAMEVLHELTDHPIYLETEEHFIQNYMS : 1797
QOY24398.1 : YLKENYRANLPLFNKMFNILISQGELLKQFIQYTNVQLARPNLTALLGANNDSVIYYNNN-NVPATGLSVGQAALRGIGGVFRPNVTLMPLGDAQNNTSDVVRKRLVAVIDGIIRGSHTLADSAMEVLHELTDHPIYLETEEHFIQNYMS : 1797
QSG73791.1 : YLKENYRANLPLFNKMFNILISQGELLKQFIQYTNVQLARPNLTALLGANNDSVIYYNNN-NVPATGLSVGQAALRGIGGVFRPNVTLMPLGDAQNNTSDVVRKRLVAVIDGIIRGSHTLADSAMEVLHELTDHPIYLETEEHFIQNYMS : 1797
QTE18752.1 : YLKENYRANLPLFNKMFNILISQGELLKQFIQYTNVQLARPNLTALLGANNDSVIYYNNN-NVPATGLSVGQAALRGIGGVFRPNVTLMPLGDAQNNTSDVVRKRLVAVIDGIIRGSHTLADSAMEVLHELTDHPIYLETEEHFIQNYMS : 1797
VFV48026.1 : YLKENYRANLPLFNKMFNILISQGELLKQFIQYTNVQLARPNLTALLGANNDSVIYYNNN-NVPATGLSVGQAALRGIGGVFRPNVTLMPLGDAQNNTSDVVRKRLVAVIDGIIRGSHTLADSAMEVLHELTDHPIYLETEEHFIQNYMS : 1797
```

```
QOW03225.1 : YLKENYRANLPLFNKMFNILISQGELLKQFIQYTNVQLARPNLTALLGANNDSVIYYNNN-NVPATGLSVGQAALRGIGGVFRPNVTLMPLGDAQNNTSDVVRKRLVAVIDGIIRGSHTLADSAMEVLHELTDHPIYLETEEHFIQNYMS : 1797
QOW03039.1 : YLKENYRANLPLFNKMFNILISQGELLKQFIQYTNVQLARPNLTALLGANNDSVIYYNNN-NVPATGLSVGQAALRGIGGVFRPNVTLMPLGDAQNNTSDVVRKRLVAVIDGIIRGSHTLADSAMEVLHELTDHPIYLETEEHFIQNYMS : 1797
VVW94185.1 : YLKENYRANLPLFNKMFNILISQGELLKQFIQYTNVQLARPNLTALLGANNDSVIYYNNN-NVPATGLSVGQAALRGIGGVFRPNVTLMPLGDAQNNTSDVVRKRLVAVIDGIIRGSHTLADSAMEVLHELTDHPIYLETEEHFIQNYMS : 1797
CAD0059543 : YLKENYRANLPLFNKMFNILISQGELLKQFIQYTNVQLARPNLTALLGANNDSVIYYNNN-NVPATGLSVGQAALRGIGGVFRPNVTLMPLGDAQNNTSDVVRKRLVAVIDGIIRGSHTLADSAMEVLHELTDHPIYLETEEHFIQNYMS : 1797
QXP50051.1 : YLKENYRANLPLFNKMFNILISQGELLKQFIQYTNVQLARPNLTALLGANNDSVIYYNNN-NVPATGLSVGQAALRGIGGVFRPNVTLMPLGDAQNNTSDVVRKRLVAVIDGIIRGSHTLADSAMEVLHELTDHPIYLETEEHFIQNYMS : 1797
CAD7112603 : YLKENYRANLPLFNKMFNILISQGELLKQFIQYTNVQLARPNLTALLGANNDSVIYYNNN-NVPATGLSVGQAALRGIGGVFRPNVTLMPLGDAQNNTSDVVRKRLVAVIDGIIRGSHTLADSAMEVLHELTDHPIYLETEEHFIQNYMS : 1797
AXZ96054.1 : YLKENYRANLPLFNKMFNILISQGELLKQFIQYTNVQLARPNLTALLGANNDSVIYYNNN-NVPATGLSVGQAALRGIGGVFRPNVTLMPLGDAQNNTSDVVRKRLVAVIDGIIRGSHTLADSAMEVLHELTDHPIYLETEEHFIQNYMS : 1797
QED90535.1 : YLKENYRANLPLFNKMFNILISQGELLKQFIQYTNVQLARPNLTALLGANNDSVIYYNNN-NVPATGLSVGQAALRGIGGVFRPNVTLMPLGDAQNNTSDVVRKRLVAVIDGIIRGSHTLADSAMEVLHELTDHPIYLETEEHFIQNYMS : 1797
VVW94190.1 : YLKENYRANLPLFNKMFNILISQGELLKQFIQYTNVQLARPNLTALLGANNDSVIYYNNN-NVPATGLSVGQAALRGIGGVFRPNVTLMPLGDAQNNTSDVVRKRLVAVIDGIIRGSHTLADSAMEVLHELTDHPIYLETEEHFIQNYMS : 1797
QEY87898.1 : YLKENYRANLPLFNKMFNILISQGELLKQFIQYTNVQLARPNLTALLGANNDSVIYYNNN-NVPATGLSVGQAALRGIGGVFRPNVTLMPLGDAQNNTSDVVRKRLVAVIDGIIRGSHTLADSAMEVLHELTDHPIYLETEEHFIQNYMS : 1797
QGV56805.1 : YLKENYRANLPLFNKMFNILISQGELLKQFIQYTNVQLARPNLTALLGANNDSVIYYNNN-NVPATGLSVGQAALRGIGGVFRPNVTLMPLGDAQNNTSDVVRKRLVAVIDGIIRGSHTLADSAMEVLHELTDHPIYLETEEHFIQNYMS : 1797
QPB67618.1 : YLKENYRANLPLFNKMFNILISQGELLKQFIQYTNVQLARPNLTALLGANNDSVIYYNNN-NVPATGLSVGQAALRGIGGVFRPNVTLMPLGDAQNNTSDVVRKRLVAVIDGIIRGSHTLADSAMEVLHELTDHPIYLETEEHFIQNYMS : 1797
QUQ60377.1 : YLKENYRANLPLFNKMFNILISQGELLKQFIQYTNVQLARPNLTALLGANNDSVIYYNNN-NVPATGLSVGQAALRGIGGVFRPNVTLMPLGDAQNNTSDVVRKRLVAVIDGIIRGSHTLADSAMEVLHELTDHPIYLETEEHFIQNYMS : 1797
QUQ60197.1 : YLKENYRANLPLFNKMFNILISQGELLKQFIQYTNVQLARPNLTALLGANNDSVIYYNNN-NVPATGLSVGQAALRGIGGVFRPNVTLMPLGDAQNNTSDVVRKRLVAVIDGIIRGSHTLADSAMEVLHELTDHPIYLETEEHFIQNYMS : 1797
SPS73515.1 : YLKENYRANLPLFNKMFNILISQGELLKQFIQYTNVQLARPNLTALLGANNDSVIYYNNN-NVPATGLSVGQAALRGIGGVFRPNVTLMPLGDAQNNTSDVVRKRLVAVIDGIIRGSHTLADSAMEVLHELTDHPIYLETEEHFIQNYMS : 1797
QDL88123.1 : YLKENYRANLPLFNKMFNILISQGELLKQFIQYTNVQLARPNLTALLGANNDSVIYYNNN-NVPATGLSVGQAALRGIGGVFRPNVTLMPLGDAQNNTSDVVRKRLVAVIDGIIRGSHTLADSAMEVLHELTDHPIYLETEEHFIQNYMS : 1797
QIA61506.1 : YLKENYRANLPLFNKMFNILISQGELLKQFIQYTNVQLARPNLTALLGANNDSVIYYNNN-NVPATGLSVGQAALRGIGGVFRPNVTLMPLGDAQNNTSDVVRKRLVAVIDGIIRGSHTLADSAMEVLHELTDHPIYLETEEHFIQNYMS : 1797
QTZ19752.1 : YLKENYRANLPLFNKMFNILISQGELLKQFIQYTNVQLARPNLTALLGANNDSVIYYNNN-NVPATGLSVGQAALRGIGGVFRPNVTLMPLGDAQNNTSDVVRKRLVAVIDGIIRGSHTLADSAMEVLHELTDHPIYLETEEHFIQNYMS : 1797
QIM07715.1 : YLKENYRANLPLFNKMFNILISQGELLKQFIQYTNVQLARPNLMGLLGANNDSVIYYNNNINVPMTGLSVGQAALRGIGGVFRPNVTLMPLGDAQNNTSDVVRKRLVAVIDGIIRGSHTLADSAMEVLHELTDHPIYLETEEHFIQNYMS : 1797
QIM07948.1 : YLKENYRANLPLFNKMFNILISQGELLKQFIQYTNVQLARPNLMGLLGANNDSVIYYNNNINVPMTGLSVGQAALRGIGGVFRPNVTLMPLGDAQNNTSDVVRKRLVAVIDGIIRGSHTLADSAMEVLHELTDHPIYLETEEHFIQNYMS : 1797
QIM08183.1 : YLKENYRANLPLFNKMFNILISQGELLKQFIQYTNVQLARPNLMGLLGANNDSVIYYNNNINVPMTGLSVGQAALRGIGGVFRPNVTLMPLGDAQNNTSDVVRKRLVAVIDGIIRGSHTLADSAMEVLHELTDHPIYLETEEHFIQNYMS : 1797
YP_0097024 : YLKENYRANLPLFNKMFNILISQGELLKQFIQYTNVQLARPNLMGLLGANNDSVIYYNNNINVPMTGLSVGQAALRGIGGVFRPNVTLMPLGDAQNNTSDVVRKRLVAVIDGIIRGSHTLADSAMEVLHELTDHPIYLETEEHFIQNYMS : 1797
YP_0097038 : YLKENYRANLPLFNKMFNILISQGELLKQFIQYTNVQLARPNLMGLLGANNDSVIYYNNNINVPMTGLSVGQAALRGIGGVFRPNVTLMPLGDAQNNTSDVVRKRLVAVIDGIIRGSHTLADSAMEVLHELTDHPIYLETEEHFIQNYMS : 1797
YP_0097023 : YLKENYRANLPLFNKMFNILISQGELLKQFIQYTNVQLARPNLMGLLGANNDSVIYYNNNINVPMTGLSVGQAALRGIGGVFRPNVTLMPLGDAQNNTSDVVRKRLVAVIDGIIRGSHTLADSAMEVLHELTDHPIYLETEEHFIQNYMS : 1797
YP_0097026 : YLKENYRANLPLFNKMFNILISQGELLKQFIQYTNVQLARPNLMGLLGANNDSVIYYNNNINVPMTGLSVGQAALRGIGGVFRPNVTLMPLGDAQNNTSDVVRKRLVAVIDGIIRGSHTLADSAMEVLHELTDHPIYLETEEHFIQNYMS : 1797
YP_0097035 : YLKENYRANLPLFNKMFNILISQGELLKQFIQYTNVQLARPNLMGLLGANNDSVIYYNNNINVPMTGLSVGQAALRGIGGVFRPNVTLMPLGDAQNNTSDVVRKRLVAVIDGIIRGSHTLADSAMEVLHELTDHPIYLETEEHFIQNYMS : 1797
QZK26792.1 : YLKENYRANLPLFNKMFNILISQGELLKQFIQYTNVQLARPNLTALLGANNDSVIYYNNNINVPTTGLSVGQAALRGIGGVFRPNVTLMPLGDAQNNTSDIVRKRLVAVIDGIIRGSHTLADSAMEVLHELTDHPIYLETEEHFIQNYMS : 1797
YP_0097036 : YLKENYRANLPLFNKMFNILISQGELLKQFIQYTNVQLARPNLMGLLGANNDSVIYYNNNINVPMTGLSVGQAALRGIGGVFRPNVTLMPLGDAQNNTSDVVRKRLVAVIDGIIRGSHTLADSAMEVLHELTDHPIYLETEEHFIQNYMS : 1797
QID21266.1 : YLKENYRANLPLFNKMFNILISQGELLKQFIQYTNVQLARPNLMGLLGANNDSIIYYNNN-NVPTTGLSVGQAALRGIGGVFRPNVTLMPLGDAQNNTSDIVRKRLVAVIDGIIRGSHTLADSAMEVLHELTDHPIYLETEEHFIQNYMS : 1796
P0CA04.1   : YLKENYRANLPLFNKMFNILISQGELLKQFIQYTNVQLARPNLMGLLGANNDSIIYYNNN-NVPTTGLSVGQAALRGIGGVFRPNVTLMPLGDAQNNTSDIVRKRLVAVIDGIIRGSHTLADSAMEVLHELTDHPIYLETEEHFIQNYMS : 1796
P0CA02.1   : YLKENYRANLPLFNKMFNILISQGELLKQFIQYTNVQLARPNLTALLRANNDSVIYYNNN-NVPMTGLSIGQAAMRGIGGVFRPNVTLMPLGDAQNNTSDIVRKRLVAVIDGIIRGSHTLADSAMEVLHELTDHPIYLETEEHFIQNYMS : 1796
YP_0097029 : YLKENYRANLPLYNKMFNILISQGELLKQFIQYTKVQLARPNLMALLGANNDAIIYYNN-INVPNTGLSIGQAALRGIGSVFRPDITLMPLGDAQNNTNDIVRKRLIAVINGVIRGSLTLANSAMEVLHELTDHPIYLETEEHFIQNYMS : 1797
AXB50010.1 : YLKENYRANLPLYNKMFNILISQGELLKQFIQYTKVQLARPNLMALLGANNDAIIYYNN-INVPNTGLSIGQAALRGIGSVFRPDITLMPLGDAQNNTNDIVRKRLIAVINGVIRGSLTLANSAMEVLHELTDHPIYLETEEHFIQNYMS : 1798
AXB49666.1 : YLKENYRANLPLYNKMFNILISQGELLKQFIQYTKVQLARPNLMALLGANNDAIIYYNN-INVPNTGLSIGQAALRGIGSVFRPDITLMPLGDAQNNTNDIVRKRLIAVINGVIRGSLTLANSAMEVLHELTDHPIYLETEEHFIQNYMS : 1798
CAD7112308 : YLKENYRANLPLFNKMFNILISQGELLKQFIQYTKVQLARPNLTALLGANNDSIIYYNNN-NVPNTGLTIGQAALRGIGSVFRPDITLMPLGNAQNNTNDVVRKRLIAVINGIIRGSLTLANSAMEVLHELTDHPIYFETEEHFIQNYMS : 1796
QRY19118.1 : YLKENYRANLPLFNKMFNILISQGELLKQFIQYTKVQLARPNLTALLGANNDSIIYYNNN-NVPNTGLTVGQAALRGIGSVFRPDITLMPLGNAQNNTNDVVRKRLIAVINGIIRGSLTLANSAMEVLHELTDHPIYFETEEHFIQNYMS : 1796
P0CA01.1   : YLKENYRANLPLFNKMFNILISQGELLKQFIQYTKVQLARPNLTALLGANNDSIIYYNNN-NVPNTGLTVGQAALRGIGSVFRPDITLMPLGNAQNNTNDVVRKRLIAVINGIIRGSLTLANSAMEVLHELTDHPIYFETEEHFIQNYMS : 1796
YP_0097028 : YLKENYRANLPLFNKMFNILISQGELLKQFIQYTKVQLARPNLTALLGANNDSIIYYNNN-NVPNTGLTVGQAALRGIGSVFRPDITLMPLGNAQNNTNDVVRKRLIAVINGIIRGSLTLANSAMEVLHELTDHPIYFETEEHFIQNYMS : 1796
P0CA03.1   : YLKENYRANLPLFNKMFNILISQGELLKQFIQYTNVQLARPNLTALLGANNDSVIYYNNNINVPMTGLSVGQAAMRGIGGVFRPNVTLMPLGDAQSNTSDIVRKRLVAVIDGIIRGSHTLADSAMEVLHELTDHPIYLETEEHFIQNYMS : 1796
                                                                                         i

                      *      1820         *      1840         *      1860         *      1880         *      1900         *      1920         *      1940         *
YP_0099272 : RYNKEPLMPFSLSLYYLHDLRIENNEVYDPLLYPNLESGSPEFKLLYGTRKLLGNDPVQLSDMPGVQLIMKNYNETVVAREQITPTRFEHFYTHAIQALRFIINIRSFKTVMMYNENTFGGVNLISENRDDKPIITAGIGMNAVYSLRKT : 1947
AZP54139.1 : RYNKEPLMPFSLSLYYLHDLRIENNEVYDPLLYPNLESGSPEFKLLYGTRKLLGNDPVQLSDMPGVQLIMKNYNETVVAREQITPTRFEHFYTHAIQALRFIINIRSFKTVMMYNENTFGGVNLISENRDDKPIITAGIGMNAVYSLRKT : 1947
QIE06881.1 : RYNKEPLMPFSLSLYYLHDLRIENNEVYDPLLYPNLESGSPEFKLLYGTRKLLGNDPVQLSDMPGVQLIMKNYNETVVAREQITPTRFEHFYTHAIQALRFIINIRSFKTVMMYNENTFGGVNLISENRDDKPIITAGIGMNAVYSLRKT : 1947
QGJ83454.1 : RYNKEPLMPFSLSLYYLHDLRIENNEVYDPLLYPNLESGSPEFKLLYGTRKLLGNDPVQLSDMPGVQLIMKNYNETVVAREQITPTRFEHFYTHAIQALRFIINIRSFKTVMMYNENTFGGVNLISENRDDKPIITAGIGMNAVYSLRKT : 1947
AYW34063.1 : RYNKEPLMPFSLSLYYLHDLRIENNEVYDPLLYPNLESGSPEFKLLYGTRKLLGNDPVQLSDMPGVQLIMKNYNETVVAREQITPTRFEHFYTHAIQALRFIINIRSFKTVMMYNENTFGGVNLISENRDDKPIITAGIGMNAVYSLRKT : 1947
QLF78620.1 : RYNKEPLMPFSLSLYYLHDLRIENNEVYDPLLYPNLESGSPEFKLLYGTRKLLGNDPVQLSDMPGVQLIMKNYNETVVAREQITPTRFEHFYTHAIQALRFIINIRSFKTVMMYNENTFGGVNLISENRDDKPIITAGIGMNAVYSLRKT : 1947
QTP96400.1 : RYNKEPLMPFSLSLYYLHDLRIENNEVYDPLLYPNLESGSPEFKLLYGTRKLLGNDPVQLSDMPGVQLIMKNYNETVVAREQITPTRFEHFYTHAIQALRFIINIRSFKTVMMYNENTFGGVNLISENRDDKPIITAGIGMNAVYSLRKT : 1947
QBH90765.1 : RYNKEPLMPFSLSLYYLHDLRIENNEVYDPLLYPNLESGSPEFKLLYGTRKLLGNDPVQLSDMPGVQLIMKNYNETVVAREQITPTRFEHFYTHAIQALRFIINIRSFKTVMMYNENTFGGVNLISENRDDKPIITAGIGMNAVYSLRKT : 1947
QOY24398.1 : RYNKEPLMPFSLSLYYLHDLRIENNEVYDPLLYPNLESGSPEFKLLYGTRKLLGNDPVQLSDMPGVQLIMKNYNETVVAREQITPTRFEHFYTHAIQALRFIINIRSFKTVMMYNENTFGGVNLISENRDDKPIITAGIGMNAVYSLRKT : 1947
QSG73791.1 : RYNKEPLMPFSLSLYYLHDLRIENNEVYDPLLYPNLESGSPEFKLLYGTRKLLGNDPVQLSDMPGVQLIMKNYNETVVAREQITPTRFEHFYTHAIQALRFIINIRSFKTVMMYNENTFGGVNLISENRDDKPIITAGIGMNAVYSLRKT : 1947
QTE18752.1 : RYNKEPLMPFSLSLYYLHDLRIENNEVYDPLLYPNLESGSPEFKLLYGTRKLLGNDPVQLSDMPGVQLIMKNYNETVVAREQITPTRFEHFYTHAIQALRFIINIRSFKTVMMYNENTFGGVNLISENRDDKPIITAGIGMNAVYSLRKT : 1947
VFV48026.1 : RYNKEPLMPFSLSLYYLHDLRIENNEVYDPLLYPNLESGSPEFKLLYGTRKLLGNDPVQLSDMPGVQLIMKNYNETVVAREQITPTRFEHFYTHAIQALRFIINIRSFKTVMMYNENTFGGVNLISENRDDKPIITAGIGMNAVYSLRKT : 1947
QOW03225.1 : RYNKEPLMPFSLSLYYLHDLRIENNEVYDPLLYPNLESGSPEFKLLYGTRKLLGNDPVQLSDMPGVQLIMKNYNETVVAREQITPTRFEHFYTHAIQALRFIINIRSFKTVMMYNENTFGGVNLISENRDDKPIITAGIGMNAVYSLRKT : 1947
QOW03039.1 : RYNKEPLMPFSLSLYYLHDLRIENNEVYDPLLYPNLESGSPEFKLLYGTRKLLGNDPVQLSDMPGVQLIMKNYNETVVAREQITPTRFEHFYTHAIQALRFIINIRSFKTVMMYNENTFGGVNLISENRDDKPIITAGIGMNAVYSLRKT : 1947
VVW94185.1 : RYNKEPLMPFSLSLYYLHDLRIENNEVYDPLLYPNLESGSPEFKLLYGTRKLLGNDPVQLSDMPGVQLIMKNYNETVVAREQITPTRFEHFYTHAIQALRFIINIRSFKTVMMYNENTFGGVNLISENRDDKPIITAGIGMNAVYSLRKT : 1947
CAD0059543 : RYNKEPLMPFSLSLYYLHDLRIENNEVYDPLLYPNLESGSPEFKLLYGTRKLLGNDPVQLSDMPGVQLIMKNYNETVVAREQITPTRFEHFYTHAIQALRFIINIRSFKTVMMYNENTFGGVNLISENRDDKPIITAGIGMNAVYSLRKT : 1947
QXP50051.1 : RYNKEPLMPFSLSLYYLHDLRIENNEVYDPLLYPNLESGSPEFKLLYGTRKLLGNDPVQLSDMPGVQLIMKNYNETVVAREQITPTRFEHFYTHAIQALRFIINIRSFKTVMMYNENTFGGVNLISENRDDKPIITAGIGMNAVYSLRKT : 1947
CAD7112603 : RYNKEPLMPFSLSLYYLHDLRIENNEVYDPLLYPNLESGSPEFKLLYGTRKLLGNDPVQLSDMPGVQLIMKNYNETVVAREQITPTRFEHFYTHAIQALRFIINIRSFKTVMMYNENTFGGVNLISENRDDKPIITAGIGMNAVYSLRKT : 1947
AXZ96054.1 : RYNKEPLMPFSLSLYYLHDLRIENNEVYDPLLYPNLESGSPEFKLLYGTRKLLGNDPVQLSDMPGVQLIMKNYNETVVAREQITPTRFEHFYTHAIQALRFIINIRSFKTVMMYNENTFGGVNLISENRDDKPIITAGIGMNAVYSLRKT : 1947
QED90535.1 : RYNKEPLMPFSLSLYYLHDLRIENNEVYDPLLYPNLESGSPEFKLLYGTRKLLGNDPVQLSDMPGVQLIMKNYNETVVAREQITPTRFEHFYTHAIQALRFIINIRSFKTVMMYNENTFGGVNLISENRDDKPIITAGIGMNAVYSLRKT : 1947
VVW94190.1 : RYNKEPLMPFSLSLYYLHDLRIENNEVYDPLLYPNLESGSPEFKLLYGTRKLLGNDPVQLSDMPGVQLIMKNYNETVVAREQITPTRFEHFYTHAIQALRFIINIRSFKTVMMYNENTFGGVNLISENRDDKPIITAGIGMNAVYSLRKT : 1947
QEY87898.1 : RYNKEPLMPFSLSLYYLHDLRIENNEVYDPLLYPNLESGSPEFKLLYGTRKLLGNDPVQLSDMPGVQLIMKNYNETVVAREQITPTRFEHFYTHAIQALRFIINIRSFKTVMMYNENTFGGVNLISENRDDKPIITAGIGMNAVYSLRKT : 1947
QGV56805.1 : RYNKEPLMPFSLSLYYLHDLRIENNEVYDPLLYPNLESGSPEFKLLYGTRKLLGNDPVQLSDMPGVQLIMKNYNETVVAREQITPTRFEHFYTHAIQALRFIINIRSFKTVMMYNENTFGGVNLISENRDDKPIITAGIGMNAVYSLRKT : 1947
QPB67618.1 : RYNKEPLMPFSLSLYYLHDLRIENNEVYDPLLYPNLESGSPEFKLLYGTRKLLGNDPVQLSDMPGVQLIMKNYNETVVAREQITPTRFEHFYTHAIQALRFIINIRSFKTVMMYNENTFGGVNLISENRDDKPIITAGIGMNAVYSLRKT : 1947
QUQ60377.1 : RYNKEPLMPFSLSLYYLHDLRIENNEVYDPLLYPNLESGSPEFKLLYGTRKLLGNDPVQLSDMPGVQLIMKNYNETVVAREQITPTRFEHFYTHAIQALRFIINIRSFKTVMMYNENTFGGVNLISENRDDKPIITAGIGMNAVYSLRKT : 1947
QUQ60197.1 : RYNKEPLMPFSLSLYYLHDLRIENNEVYDPLLYPNLESGSPEFKLLYGTRKLLGNDPVQLSDMPGVQLIMKNYNETVVAREQITPTRFEHFYTHAIQALRFIINIRSFKTVMMYNENTFGGVNLISENRDDKPIITAGIGMNAVYSLRKT : 1947
```

```
SPS73515.1 : RYNKEPLMPFSLSLYYLHDLRIENNEVYDPLLYPNLESGSPEFKLLYGTRKLLGNDPVQLSDMPGVQLIMKNYNETVVAREQITPTRFEHFYTHAIQALRFIINIRSFKTVMMYNENTFGGVNLISENRDDKPIITAGIGMNAVYSLRKT : 1947
QDL88123.1 : RYNKEPLMPFSLSLYYLHDLRIENNEVYDPLLYPNLESGSPEFKLLYGTRKLLGNDPVQLSDMPGVQLIMKNYNETVVAREQITPTRFEHFYTHAIQALRFIINIRSFKTVMMYNENTFGGVNLISENRDDKPIITAGIGMNAVYSLRKT : 1947
QIA61506.1 : RYNKEPLMPFSLSLYYLHDLRIENNEVYDPLLYPNLESGSPEFKLLYGTRKLLGNDPVQLSDMPGVQLIMKNYNETVVAREQITPTRFEHFYTHAIQALRFIINIRSFKTVMMYNENTFGGVNLISENRDDKPIITAGIGMNAVYSLRKT : 1947
QTZ19752.1 : RYNKEPLMPFSLSLYYLHDLRIENNEVYDPLLYPNLESGSPEFKLLYGTRKLLGNDPVQLSDMPGVQLIMKNYNETVVAREQITPTRFEHFYTHAIQALRFIINIRSFKTVMMYNENTFGGVNLISENRDDKPIITAGIGMNAVYSLRKT : 1947
QIM07715.1 : RYNKEPLMPFSLSLYYLRDLRIENNEVYDPLLYPNLESGSPEFKLLYGTRKLLGNDPVQLSDMPGVQLIMKNYNETVVAHEQITPTRFEHFYTHAIQALRFIVNIRSFKTVMMYNENTFGGVNLISENRDDKPIITAGIGMNAVYSLRKT : 1947
QIM07948.1 : RYNKEPLMPFSLSLYYLRDLRIENNEVYDPLLYPNLESGSPEFKLLYGTRKLLGNDPVQLSDMPGVQLIMKNYNETVVAHEQITPTRFEHFYTHAIQALRFIVNIRSFKTVMMYNENTFGGVNLISENRDDKPIITAGIGMNAVYSLRKT : 1947
QIM08183.1 : RYNKEPLMPFSLSLYYLRDLRIENNEVYDPLLYPNLESGSPEFKLLYGTRKLLGNDPVQLSDMPGVQLIMKNYNETVVAHEQITPTRFEHFYTHAIQALRFIVNIRSFKTVMMYNENTFGGVNLISENRDDKPIITAGIGMNAVYSLRKT : 1947
YP_0097024 : RYNKEPLMPFSLSLYYLRDLRIENNEVYDPLLYPNLESGSPEFKLLYGTRKLLGNDPVQLSDMPGVQLIMKNYNETVVAREQITPTRFEHFYTHAIQALRFIVNIRSFKTVMMYNENTFGGVNLISENRDDKPIITAGIGMNAVYSLRKT : 1947
YP_0097038 : RYNKEPLMPFSLSLYYLRDLRIENNEVYDPLLYPNLESGSPEFKLLYGTRKLLGNDPVQLSDMPGVQLIMKNYNETVVAHEQITPTRFEHFYTHAIQALRFIVNIRSFKTVMMYNENTFGGVNLISENRDDKPIITAGIGMNAVYSLRKT : 1947
YP_0097023 : RYNKEPLMPFSLSLYYLRDLRIENNEVYDPLLYPNLESGSPEFKLLYGTRKLLGNDPVQLSDMPGVQLIMKNYNETVVAHEQITPTRFEHFYTHAIQALRFIVNIRSFKTVMMYNENTFGGVNLISENRDDKPIITAGIGMNAVYSLRKT : 1947
YP_0097026 : RYNKEPLMPFSLSLYYLRDLRIENNEVYDPLLYPNLESGSPEFKLLYGTRKLLGNDPVQLSDMPGVQLIMKNYNETVVAREQITPTRFEHFYTHAIQALRFIVNIRSFKTVMMYNENTFGGVNLISENRDDKPIITAGIGMNAVYSLRKT : 1947
YP_0097035 : RYNKEPLMPFSLSLYYLRDLRIENNEVYDPLLYPNLESGSPEFKLLYGTRKLLGNDPVQLSDMPGVQLIMKNYNETVVAHEQITPTRFEHFYTHAIQALRFIVNIRSFKTVMMYNENTFGGVNLISENRDDKPIITAGIGMNAVYSLRKT : 1947
QZK26792.1 : RYNKEPLMPFSLSLYYLRDLRIENNEVYDPLLYPNLESGSPEFKLLYGTRKLLGDDPVQLSDMPGVQLIMKNYNETVVAREQITPTRFEHFYTHAIQALRFIINIRSFKTVMMYNENTFGGVNLISENRDDKPIITAGIGMNAVYSLRKT : 1947
YP_0097036 : RYNKEPLMPFSLSLYYLRDLRIENNEVYDPLLYPNLESGSPEFKLLYGTRKLLGNDPVQLSDMPGVQLIMKNYNETVVAREQITPTRFEHFYTHAIQALRFIVNIRSFKTVMMYNENTFGGVNLISENRDDKPIITAGIGMNAVYSLRKT : 1947
QID21266.1 : RYNKEPLMPFSLSLYYLRDLRIENNEVYDPLLYPNLESGSPEFKLLYGTRKLLGNDPVQLSDMPGVQLIMKNYNETVVAREQITPTRFEHFYTHAIQALRFIINIRSFKTVMMYNENTFGGVNLISENRDDKPIITAGIGMNAVYSLRKT : 1946
P0CA04.1   : RYNKEPLMPFSLSLYYLRDLRIENNEVYDPLLYPNLESGSPEFKLLYGTRKLLGNDPVQLSDMPGVQLIMKNYNETVVAREQITPTRFEHFYTHAIQALRFIINIRSFKTVMMYNENTFGGVNLISENRDDKPIITAGIGMNAVYSLRKT : 1946
P0CA02.1   : RYNKEPLMPFSLSLYYLRDLRIENNEVYDPLLYPNLESGSPEFKLLYGTRKLLGNDPVQLSDMPGVQLIMKNYNETVVAREQITPTRFEHFYTHAIQALRFIVNIRSFKTVMTYNENTFGGVNLISEDRNDKPIITAGIGMNAVYSLRKT : 1946
YP_0097029 : RYNKEPLMPFSLSLYYLRDLRMENNEVYDPLLYPNLESGSPEFKILYGTRKLLGNDPVQLSDMPGVQLIMKNYNETVVAREQITPTRFEHFYIHAIQALRFIINIRSFKTVMTYNENTFGGVNLISENRDDKPIITAGIGMNAVYSLRKT : 1947
AXB50010.1 : RYNKEPLMPFSLSLYYLRDLRMENNEVYDPLLYPNLESGSPEFKILYGTRKLLGNDPVQLSDMPGVQLIMKNYNETVVAREQITPTRFEHFYIHAIQALRFIINIRSFKTVMTYNENTFGGVNLISENRDDKPIITAGIGMNAVYSLRKT : 1948
AXB49666.1 : RYNKEPLMPFSLSLYYLRDLRMENNEVYDPLLYPNLESGSPEFKILYGTRKLLGNDPVQLSDMPGVQLIMKNYNETVVAREQITPTRFEHFYIHAIQALRFIINIRSFKTVMTYNENTFGGVNLISENRDDKPIITAGIGMNAVYSLRKT : 1948
CAD7112308 : RYNKEPLMPFSLSLYYLRDLRIENNEVYDPLLYPNLESGSPEFKILYGTRKLLGNDPVQLSDMPGVQLIMKNYNETVVAREQITPTRFEHFYIHAIQALRFIINIRSFKTVMTYNENTFGGVNLIGEDRDDKPIITEGIGMNAVYSLRKT : 1946
QRY19118.1 : RYNKEPLMPFSLSLYYLRDLRIENNEVYDPLLYPNLESGSPEFKILYGTRKLLGNDPVQLSDMPGVQLIMKNYNETVVAREQITPTRFEHFYIHAIQALRFIINIRSFKTVMTYNENTFGGVNLIGEDRDDKPIITEGIGMNAVYSLRKT : 1946
P0CA01.1   : RYNKEPLMPFSLSLYYLRDLRIENNEVYDPLLYPNLESGSPEFKILYGTRKLLGNDPVQLSDMPGVQLIMKNYNETVVAREQITPTRFEHFYIHAIQALRFIINIRSFKTVMTYNENTFGGVNLIGEDRDDKPIITEGIGMNAVYSLRKT : 1946
YP_0097028 : RYNKEPLMPFSLSLYYLRDLRIENNEVYDPLLYPNLESGSPEFKILYGTRKLLGNDPVQLSDMPGVQLIMKNYNETVVAREQITPTRFEHFYIHAIQALRFIINIRSFKTVMTYNENTFGGVNLISEDRDDKPIITEGIGMNAVYSLRKT : 1946
P0CA03.1   : RYNKEPLMPFSLSLYYLRDLRIENNEVYDPLLYPNLESGSPEFKLLYGTRKLLGNDPVQLSDMPGVQLIMKNYNETVVAREQITPTRFEHFYTHAIQALRFIINIRSFKTVMMYNENTFGGVNLISENRDDKPIITAGIGMNAVYSLRKT : 1946

                     1960        *       1980        *       2000        *       2020        *       2040        *       2060        *       2080        *       2100
YP_0099272 : LQDVISFVESSYQEEQINHIHKIVSPKGQTRTLGSNRERERIFNLFDMNIIPINVNALMRSIPLANIYNYDYSFEEIACLMYGISAEKVRSLDTTAPQPDVAEVLNIPNRPPINTREFMLKLLINPYVSVSITQYGNELLSKGNAGYMSR : 2097
AZP54139.1 : LQDVISFVESSYQEEQINHIHKIVSPKGQTRTLGSNRERERIFNLFDMNIIPINVNALMRSIPLANIYNYDYSFEEIACLMYGISAEKVRSLDTTAPQPDVAEVLNIPNRPPINTREFMLKLLINPYVSVSITQYGNELLSKGNAGYMSR : 2097
QIE06881.1 : LQDVISFVESSYQEEQINHIHKIVSPKGQTRTLGSNRERERIFNLFDMNIIPINVNALMRSIPLANIYNYDYSFEEIACLMYGISAEKVRSLDTTAPQPDVAEVLNIPNRPPINTREFMLKLLINPYVSVSITQYGNELLSKGNAGYMSR : 2097
QGJ83454.1 : LQDVISFVESSYQEEQINHIHKIVSPKGQTRTLGSNRERERIFNLFDMNIIPINVNALMRSIPLANIYNYDYSFEEIACLMYGISAEKVRSLDTTAPQPDVAEVLNIPNRPPINTREFMLKLLINPYVSVSITQYGNELLSKGNAGYMSR : 2097
AYW34063.1 : LQDVISFVESSYQEEQINHIHKIVSPKGQTRTLGSNRERERIFNLFDMNIIPINVNALMRSIPLANIYNYDYSFEEIACLMYGISAEKVRSLDTTAPQPDVAEVLNIPNRPPINTREFMLKLLINPYVSVSITQYGNELLSKGNAGYMSR : 2097
QLF78620.1 : LQDVISFVESSYQEEQINHIHKIVSPKGQTRTLGSNRERERIFNLFDMNIIPINVNALMRSIPLANIYNYDYSFEEIACLMYGISAEKVRSLDTTAPQPDVAEVLNIPNRPPINTREFMLKLLINPYVSVSITQYGNELLSKGNAGYMSR : 2097
QTP96400.1 : LQDVISFVESSYQEEQINHIHKIVSPKGQTRTLGSNRERERIFNLFDMNIIPINVNALMRSIPLANIYNYDYSFEEIACLMYGISAEKVRSLDTTAPQPDVAEVLNIPNRPPINTREFMLKLLINPYVSVSITQYGNELLSKGNAGYMSR : 2097
QBH90765.1 : LQDVISFVESSYQEEQINHIHKIVSPKGQTRTLGSNRERERIFNLFDMNIIPINVNALMRSIPLANIYNYDYSFEEIACLMYGISAEKVRSLDTTAPQPDVAEVLNIPNRPPINTREFMLKLLINPYVSVSITQYGNELLSKGNAGYMSR : 2097
QOY24398.1 : LQDVISFVESSYQEEQINHIHKIVSPKGQTRTLGSNRERERIFNLFDMNIIPINVNALMRSIPLANIYNYDYSFEEIACLMYGISAEKVRSLDTTAPQPDVAEVLNIPNRPPINTREFMLKLLINPYVSVSITQYGNELLSKGNAGYMSR : 2097
QSG73791.1 : LQDVISFVESSYQEEQINHIHKIVSPKGQTRTLGSNRERERIFNLFDMNIIPINVNALMRSIPLANIYNYDYSFEEIACLMYGISAEKVRSLDTTAPQPDVAEVLNIPNRPPINTREFMLKLLINPYVSVSITQYGNELLSKGNAGYMSR : 2097
QTE18752.1 : LQDVISFVESSYQEEQINHIHKIVSPKGQTRTLGSNRERERIFNLFDMNIIPINVNALMRSIPLANIYNYDYSFEEIACLMYGISAEKVRSLDTTAPQPDVAEVLNIPNRPPINTREFMLKLLINPYVSVSITQYGNELLSKGNAGYMSR : 2097
VFV48026.1 : LQDVISFVESSYQEEQINHIHKIVSPKGQTRTLGSNRERERIFNLFDMNIIPINVNALMRSIPLANIYNYDYSFEEIACLMYGISAEKVRSLDTTAPQPDVAEVLNIPNRPPINTREFMLKLLINPYVSVSITQYGNELLSKGNAGYMSR : 2097
QOW03225.1 : LQDVISFVESSYQEEQINHIHKIVSPKGQTRTLGSNRERERIFNLFDMNIIPINVNALMRSIPLANIYNYDYSFEEIACLMYGISAEKVRSLDTTAPQPDVAEVLNIPNRPPINTREFMLKLLINPYVSVSITQYGNELLSKGNAGYMSR : 2097
QOW03039.1 : LQDVISFVESSYQEEQINHIHKIVSPKGQTRTLGSNRERERIFNLFDMNIIPINVNALMRSIPLANIYNYDYSFEEIACLMYGISAEKVRSLDTTAPQPDVAEVLNIPNRPPINTREFMLKLLINPYVSVSITQYGNELLSKGNAGYMSR : 2097
VVW94185.1 : LQDVISFVESSYQEEQINHIHKIVSPKGQTRTLGSNRERERIFNLFDMNIIPINVNALMRSIPLANIYNYDYSFEEIACLMYGISAEKVRSLDTTAPQPDVAEVLNIPNRPPINTREFMLKLLINPYVSVSITQYGNELLSKGNAGYMSR : 2097
CAD0059543 : LQDVISFVESSYQEEQINHIHKIVSPKGQTRTLGSNRERERIFNLFDMNIIPINVNALMRSIPLANIYNYDYSFEEIACLMYGISAEKVRSLDTTAPQPDVAEVLNIPNRPPINTREFMLKLLINPYVSVSITQYGNELLSKGNAGYMSR : 2097
QXP50051.1 : LQDVISFVESSYQEEQINHIHKIVSPKGQTRTLGSNRERERIFNLFDMNIIPINVNALMRSIPLANIYNYDYSFEEIACLMYGISAEKVRSLDTTAPQPDVAEVLNIPNRPPINTREFMLKLLINPYVSVSITQYGNELLSKGNAGYMSR : 2097
CAD7112603 : LQDVISFVESSYQEEQINHIHKIVSPKGQTRTLGSNRERERIFNLFDMNIIPINVNALMRSIPLANIYNYDYSFEEIACLMYGISAEKVRSLDTTAPQPDVAEVLNIPNRPPINTREFMLKLLINPYVSVSITQYGNELLSKGNAGYMSR : 2097
AXZ96054.1 : LQDVISFVESSYQEEQINHIHKIVSPKGQTRTLGSNRERERIFNLFDMNIIPINVNALMRSIPLANIYNYDYSFEEIACLMYGISAEKVRSLDTTAPQPDVAEVLNIPNRPPINTREFMLKLLINPYVSVSITQYGNELLSKGNAGYMSR : 2097
QED90535.1 : LQDVISFVESSYQEEQINHIHKIVSPKGQTRTLGSNRERERIFNLFDMNIIPINVNALMRSIPLANIYNYDYSFEEIACLMYGISAEKVRSLDTTAPQPDVAEVLNIPNRPPINTREFMLKLLINPYVSVSITQYGNELLSKGNAGYMSR : 2097
VVW94190.1 : LQDVISFVESSYQEEQINHIHKIVSPKGQTRTLGSNRERERIFNLFDMNIIPINVNALMRSIPLANIYNYDYSFEEIACLMYGISAEKVRSLDTTAPQPDVAEVLNIPNRPPINTREFMLKLLINPYVSVSITQYGNELLSKGNAGYMSR : 2097
QEY87898.1 : LQDVISFVESSYQEEQINHIHKIVSPKGQTRTLGSNRERERIFNLFDMNIIPINVNALMRSIPLANIYNYDYSFEEIACLMYGISAEKVRSLDTTAPQPDVAEVLNIPNRPPINTREFMLKLLINPYVSVSITQYGNELLSKGNAGYMSR : 2097
QGV56805.1 : LQDVISFVESSYQEEQINHIHKIVSPKGQTRTLGSNRERERIFNLFDMNIIPINVNALMRSIPLANIYNYDYSFEEIACLMYGISAEKVRSLDTTAPQPDVAEVLNIPNRPPINTREFMLKLLINPYVSVSITQYGNELLSKGNAGYMSR : 2097
QPB67618.1 : LQDVISFVESSYQEEQINHIHKIVSPKGQTRTLGSNRERERIFNLFDMNIIPINVNALMRSIPLANIYNYDYSFEEIACLMYGISAEKVRSLDTTAPQPDVAEVLNIPNRPPINTREFMLKLLINPYVSVSITQYGNELLSKGNAGYMSR : 2097
QUQ60377.1 : LQDVISFVESSYQEEQINHIHKIVSPKGQTRTLGSNRERERIFNLFDMNIIPINVNALMRSIPLANIYNYDYSFEEIACLMYGISAEKVRSLDTTAPQPDVAEVLNIPNRPPINTREFMLKLLINPYVSVSITQYGNELLSKGNAGYMSR : 2097
QUQ60197.1 : LQDVISFVESSYQEEQINHIHKIVSPKGQTRTLGSNRERERIFNLFDMNIIPINVNALMRSIPLANIYNYDYSFEEIACLMYGISAEKVRSLDTTAPQPDVAEVLNIPNRPPINTREFMLKLLINPYVSVSITQYGNELLSKGNAGYMSR : 2097
SPS73515.1 : LQDVISFVESSYQEEQINHIHKIVSPKGQTRTLGSNRERERIFNLFDMNIIPINVNALMRSIPLANIYNYDYSFEEIACLMYGISAEKVRSLDTTAPQPDVAEVLNIPNRPPINTREFMLKLLINPYVSVSITQYGNELLSKGNAGYMSR : 2097
QDL88123.1 : LQDVISFVESSYQEEQINHIHKIVSPKGQTRTLGSNRERERIFNLFDMNIIPINVNALMRSIPLANIYNYDYSFEEIACLMYGISAEKVRSLDTTAPQPDVAEVLNIPNRPPINTREFMLKLLINPYVSVSITQYGNELLSKGNAGYMSR : 2097
QIA61506.1 : LQDVISFVESSYQEEQINHIHKIVSPKGQTRTLGSNRERERIFNLFDMNIIPINVNALMRSIPLANIYNYDYSFEEIACLMYGISAEKVRSLDTTAPQPDVAEVLNIPNRPPINTREFMLKLLINPYVSVSITQYGNELLSKGNAGYMSR : 2097
QTZ19752.1 : LQDVISFVESSYQEEQINHIHKIVSPKGQTRTLGSNRERERIFNLFDMNIIPINVNALMRSIPLANIYNYDYSFEEIACLMYGISAEKVRSLDTTAPQPDVAEVLNIPNRPPINTREFMLKLLINPYVSVSITQYGNELLSKGNAGYMSR : 2097
QIM07715.1 : LQDVISFVESSYQEEQINHIHKIVSPKGQTRTLGSNRERERIFNLFDMNIIPINVNALMRSIPLANIYNYDYSFEEIACLMYGISAEKVRSLNTAAPQPDIAEVLNIPNRPPMNTREFMLKLLINPYVSVSITQYGNELMSKGSAGYMSR : 2097
QIM07948.1 : LQDVISFVESSYQEEQINHIHKIVSPKGQTRTLGSNRERERIFNLFDMNIIPINVNALMRSIPLANIYNYDYSFEEIACLMYGISAEKVRSLNTAAPQPDIAEVLNIPNRPPMNTREFMLKLLINPYVSVSITQYGNELMSKGSAGYMSR : 2097
QIM08183.1 : LQDVISFVESSYQEEQINHIHKIVSPKGQTRTLGSNRERERIFNLFDMNIIPINVNALMRSIPLANIYNYDYSFEEIACLMYGISAEKVRSLNTAAPQPDIAEVLNIPNRPPMNTREFMLKLLINPYVSVSITQYGNELMSKGSAGYMSR : 2097
YP_0097024 : LQDVISFVESSYQEEQINHIHKIVSPKGQTRTLGSNRERERIFNLFDMNIIPINVNALMRSIPLANIYNYDYSFEEIACLMYGISAEKVRSLNTAAPQPDIAEVLNIPNRPPMNTREFMLKLLINPYVSVSITQYGNELMSKGSAGYMSR : 2097
YP_0097038 : LQDVISFVESSYQEEQINHIHKIVSPKGQTRTLGSNRERERIFNLFDMNIIPINVNALMRSIPLANIYNYDYSFEEIACLMYGISAEKVRSLNTAAPQPDIAEVLNIPNRPPMNTREFMLKLLINPYVSVSITQYGNELMSKGSAGYMSR : 2097
YP_0097023 : LQDVISFVESSYQEEQINHIHKIVSPKGQTRTLGSNRERERIFNLFDMNIIPINVNALMRSIPLANIYNYDYSFEEIACLMYGISAEKVRSLNTAAPQPDIAEVLNIPNRPPMNTREFMLKLLINPYVSVSITQYGNELMSKGSAGYMSR : 2097
YP_0097026 : LQDVISFVESSYQEEQINHIHKIVSPKGQTRTLGSNRERERIFNLFDMNIIPINVNALMRSIPLANIYNYDYSFEEIACLMYGISAEKVRSLNTAAPQPDIAEVLNIPNRPPMNTREFMLKLLINPYVSVSITQYGNELMSKGSAGYMSR : 2097
YP_0097035 : LQDVISFVESSYQEEQINHIHKIVSPKGQTRTLGSNRERERIFNLFDMNIIPINVNALMRSIPLANIYNYDYSFEEIACLMYGISAEKVRSLNTAAPQPDIAEVLNIPNRPPMNTREFMLKLLINPYVSVSITQYGNELMSKGSAGYMSR : 2097
QZK26792.1 : LQDVISFVESSYQEEQINHIHKIVSPKGQTRTLGSNRERERIFNLFDMNIIPINVNALMRSIPLANIYNYDYSFEEIACLMYGISAEKVRSLNTAAPQPDVAEVLNIPNRPPMNTREFMLKLLINPYVSVSITQYGNELLSKGNAGYMSR : 2097
YP_0097036 : LQDVISFVESSYQEEQINHIHKIVSPKGQTRTLGSNRERERIFNLFDMNIIPINVNALMRSIPLANIYNYDYSFEEIACLMYGISAEKVRSLNTAAPQPDIAEVLNIPNRPPMNTREFMLKLLINPYVSVSITQYGNELMSKGSAGYMSR : 2097
```

```
QID21266.1 : LQDVISFVESSYQEEQINHIHKIVSPKGQTRTLGSNRERERIFNLFDMNIIPINVNALMRSIPLANIYNYDYSFEEIACLMYGISAEKVRSLNTAAPQPDVAEVLNIPNRPPMNTREFMLKLLINPYVSVSITQYGNELLSKGNAGYMSR : 2096
P0CA04.1   : LQDVISFVESSYQEEQINHIHKIVSPKGQTRTLGSNRERERIFNLFDMNIIPINVNALMRSIPLANIYNYDYSFEEIACLMYGISAEKVRSLDTTAPQPDVAEVLNIPNRPPINTREFMLKLLINPYVSVSITQYGNELLSKGNAGYMSR : 2096
P0CA02.1   : LQDVISFVESSYQEEQINNIHKIVSPKGQTRTLGSNRERERIFNLFDMNIIPINVNALMRSIPLANIYNYDYSFEEIACLMYGISAEKVRSLNTAAPQPDVAEVLNIPNRPPINTREFMLKLLINPYVSVSITQYGNELLSKGNAGYMSR : 2096
YP_0097029 : LQDVISFVESSYQEEQINNIHKIVSPKSQTRSLGSNRERERIFNLFDMNIMPINVNALMRSIPLANIYNYDYSFEEIACLMYGISAEKVRSLDTGAPQPDVAVVLNIPNRPPMNTREFMLKLLINPYVTVSISQYGNELLSRGNAGYMSR : 2097
AXB50010.1 : LQDVISFVESSYQEEQINNIHKIVSPKSQTRSLGSNRERERIFNLFDMNIMPINVNALMRSIPLANIYNYDYSFEEIACLMYGISAEKVRSLDTGAPQPDVAVVLNIPNRPPMNTREFMLKLLINPYVTVSISQYGNELLSRGNAGYMSR : 2098
AXB49666.1 : LQDVISFVESSYQEEQINNIHKIVSPKSQTRSLGSNRERERIFNLFDMNIMPINVNALMRSIPLANIYNYDYSFEEIACLMYGISAEKVRSLDTGAPQPDVAVVLNIPNRPPMNTREFMLKLLINPYVTVSISQYGNELLSRGNAGYMSR : 2098
CAD7112308 : LQDVISFVESSYQEEQINNIHKIVSPRSQTRSLGSNRERERIFNLFDMNIMPINVNALMRSIPLANIYNYDYSFEEIACLMYGISAEKVRSLDTAAPQPDVAQVLNIPNRPPMNTREFMLKLLINPYVTVSITQYGNELLFRGNTGYMSR : 2096
QRY19118.1 : LQDVISFVESSYQEEQINNIHKIVSPRSQTRSLGSNRERERIFNLFDMNIMPINVNALMRSIPLANIYNYDYSFEEIACLMYGISAEKVRSLDTAAPQPDVAQVLNIPNRPPMNTREFMLKLLINPYVTVSITQYGNELLFRGNTGYMSR : 2096
P0CA01.1   : LQDVISFVESSYQEEQINNIHKIVSPRSQTRSLGSNRERERIFNLFDMNIMPINVNALMRSIPLANIYNYDYSFEEIACLMYGISAEKVRSLDTAAPQPDVAQVLNIPNRPPMNTREFMLKLLINPYVTVSITQYGNELLFRGNAGYMSR : 2096
YP_0097028 : LQDVISFVESSYQEEQINNIHKIVSPRSQTRSLGSNRERERIFNLFDMNIMPINVNALMRSIPLANIYNYDYSFEEIACLMYGISAEKVRSLDTAAPQPDVAQVLNIPNRPPMNTREFMLKLLINPYVTVSITQYGNELLFRGNTGYMSR : 2096
P0CA03.1   : LQDVISFVESSYQEEQINHIHKIVSPKGQTRTLGSNRERERIFNLFDMNIIPINVNALMRSIPLANIYNYDYSFEEIACLMYGISAEKVRSLNTAAPQPDIAEVLNIPNRPPMNTREFMLKLLINPYVSVSITQYGNELLSKGNAGYMSR : 2096
                                                                                                                                       l

                     *        2120        *        2140        *        2160        *        2180        *        2200        *        2220        *        2240        *
YP_0099272 : IFRGDNALNMGRPKFLSDQIFNKVLFGSLYPTQFDYDEAGPSLAAGIQRGRERWGHPMSIYINQALHEIVRTIR-LAETVRGLRNVIDRNQIIGELNAFRTQLEDTRREVNNLIQTPEIQNNPTPEIIAAIQNWVQQYRGQITNLIDLIG : 2246
AZP54139.1 : IFRGDNALNMGRPKFLSDQIFNKVLFGSLYPTQFDYDEAGPSLAAGIQRGRERWGHPMSIYINQALHEIVRTIR-LAETVRGLRNVIDRNQIIGELNAFRTQLEDTRREVNNLIQTPEIQNNPTPEIIAAIQNWVQQYRGQITNLIDLIG : 2246
QIE06881.1 : IFRGDNALNMGRPKFLSDQIFNKVLFGSLYPTQFDYDEAGPSLAAGIQRGRERWGHPMSIYINQALHEIVRTIR-LAETVRGLRNVIDRNQIIGELNAFRTQLEDTRREVNNLIQTPEIQNNPTPEIIAAIQNWVQQYRGQITNLIDLIG : 2246
QGJ83454.1 : IFRGDNALNMGRPKFLSDQIFNKVLFGSLYPTQFDYDEAGPSLAAGIQRGRERWGHPMSIYINQALHEIVRTIR-LAETVRGLRNVIDRNQIIGELNAFRTQLEDTRREVNNLIQTPEIQNNPTPEIIAAIQNWVQQYRGQITNLIDLIG : 2246
AYW34063.1 : IFRGDNALNMGRPKFLSDQIFNKVLFGSLYPTQFDYDEAGPSLAAGIQRGRERWGHPMSIYINQALHEIVRTIR-LAETVRGLRNVIDRNQIIGELNAFRTQLEDTRREVNNLIQTPEIQNNPTPEIIAAIQNWVQQYRGQITNLIDLIG : 2246
QLF78620.1 : IFRGDNALNMGRPKFLSDQIFNKVLFGSLYPTQFDYDEAGPSLAAGIQRGRERWGHPMSIYINQALHEIVRTIR-LAETVRGLRNVIDRNQIIGELNAFRTQLEDTRREVNNLIQTPEIQNNPTPEIIAAIQNWVQQYRGQITNLIDLIG : 2246
QTP96400.1 : IFRGDNALNMGRPKFLSDQIFNKVLFGSLYPTQFDYDEAGPSLAAGIQRGRERWGHPMSIYINQALHEIVRTIR-LAETVRGLRNVIDRNQIIGELNAFRTQLEDTRREVNNLIQTPEIQNNPTPEIIAAIQNWVQQYRGQITNLIDLIG : 2246
QBH90765.1 : IFRGDNALNMGRPKFLSDQIFNKVLFGSLYPTQFDYDEAGPSLAAGIQRGRERWGHPMSIYINQALHEIVRTIR-LAETVRGLRNVIDRNQIIGELNAFRTQLEDTRREVNNLIQTPEIQNNPTPEIIAAIQNWVQQYRGQITNLIDLIG : 2246
QOY24398.1 : IFRGDNALNMGRPKFLSDQIFNKVLFGSLYPTQFDYDEAGPSLAAGIQRGRERWGHPMSIYINQALHEIVRTIR-LAETVRGLRNVIDRNQIIGELNAFRTQLEDTRREVNNLIQTPEIQNNPTPEIIAAIQNWVQQYRGQITNLIDLIG : 2246
QSG73791.1 : IFRGDNALNMGRPKFLSDQIFNKVLFGSLYPTQFDYDEAGPSLAAGIQRGRERWGHPMSIYINQALHEIVRTIR-LAETVRGLRNVIDRNQIIGELNAFRTQLEDTRREVNNLIQTPEIQNNPTPEIIAAIQNWVQQYRGQITNLIDLIG : 2246
QTE18752.1 : IFRGDNALNMGRPKFLSDQIFNKVLFGSLYPTQFDYDEAGPSLAAGIQRGRERWGHPMSIYINQALHEIVRTIR-LAETVRGLRNVIDRNQIIGELNAFRTQLEDTRREVNNLIQTPEIQNNPTPEIIAAIQNWVQQYRGQITNLIDLIG : 2246
VFV48026.1 : IFRGDNALNMGRPKFLSDQIFNKVLFGSLYPTQFDYDEAGPSLAAGIQRGRERWGHPMSIYINQALHEIVRTIR-LAETVRGLRNVIDRNQIIGELNAFRTQLEDTRREVNNLIQTPEIQNNPTPEIIAAIQNWVQQYRGQITNLIDLIG : 2246
QOW03225.1 : IFRGDNALNMGRPKFLSDQIFNKVLFGSLYPTQFDYDEAGPSLAAGIQRGRERWGHPMSIYINQALHEIVRTIR-LAETVRGLRNVIDRNQIIGELNAFRTQLEDTRREVNNLIQTPEIQNNPTPEIIAAIQNWVQQYRGQITNLIDLIG : 2246
QOW03039.1 : IFRGDNALNMGRPKFLSDQIFNKVLFGSLYPTQFDYDEAGPSLAAGIQRGRERWGHPMSIYINQALHEIVRTIR-LAETVRGLRNVIDRNQIIGELNAFRTQLEDTRREVNNLIQTPEIQNNPTPEIIAAIQNWVQQYRGQITNLIDLIG : 2246
VVW94185.1 : IFRGDNALNMGRPKFLSDQIFNKVLFGSLYPTQFDYDEAGPSLAAGIQRGRERWGHPMSIYINQALHEIVRTIR-LAETVRGLRNVIDRNQIIGELNAFRTQLEDTRREVNNLIQTPEIQNNPTPEIIAAIQNWVQQYRGQITNLIDLIG : 2246
CAD0059543 : IFRGDNALNMGRPKFLSDQIFNKVLFGSLYPTQFDYDEAGPSLAAGIQRGRERWGHPMSIYINQALHEIVRTIR-LAETVRGLRNVIDRNQIIGELNAFRTQLEDTRREVNNLIQTPEIQNNPTPEIIAAIQNWVQQYRGQITNLIDLIG : 2246
QXP50051.1 : IFRGDNALNMGRPKFLSDQIFNKVLFGSLYPTQFDYDEAGPSLAAGIQRGRERWGHPMSIYINQALHEIVRTIR-LAETVRGLRNVIDRNQIIGELNAFRTQLEDTRREVNNLIQTPEIQNNPTPEIIAAIQNWVQQYRGQITNLIDLIG : 2246
CAD7112603 : IFRGDNALNMGRPKFLSDQIFNKVLFGSLYPTQFDYDEAGPSLAAGIQRGRERWGHPMSIYINQALHEIVRTIR-LAETVRGLRNVIDRNQIIGELNAFRTQLEDTRREVNNLIQTPEIQNNPTPEIIAAIQNWVQQYRGQITNLIDLIG : 2246
AXZ96054.1 : IFRGDNALNMGRPKFLSDQIFNKVLFGSLYPTQFDYDEAGPSLAAGIQRGRERWGHPMSIYINQALHEIVRTIR-LAETVRGLRNVIDRNQIIGELNAFRTQLEDTRREVNNLIQTPEIQNNPTPEIIAAIQNWVQQYRGQITNLIDLIG : 2246
QED90535.1 : IFRGDNALNMGRPKFLSDQIFNKVLFGSLYPTQFDYDEAGPSLAAGIQRGRERWGHPMSIYINQALHEIVRTIR-LAETVRGLRNVIDRNQIIGELNAFRTQLEDTRREVNNLIQTPEIQNNPTPEIIAAIQNWVQQYRGQITNLIDLIG : 2246
VVW94190.1 : IFRGDNALNMGRPKFLSDQIFNKVLFGSLYPTQFDYDEAGPSLAAGIQRGRERWGHPMSIYINQALHEIVRTIR-LAETVRGLRNVIDRNQIIGELNAFRTQLEDTRREVNNLIQTPEIQNNPTPEIIAAIQNWVQQYRGQITNLIDLIG : 2246
QEY87898.1 : IFRGDNALNMGRPKFLSDQIFNKVLFGSLYPTQFDYDEAGPSLAAGIQRGRERWGHPMSIYINQALHEIVRTIR-LAETVRGLRNVIDRNQIIGELNAFRTQLEDTRREVNNLIQTPEIQNNPTPEIIAAIQNWVQQYRGQITNLIDLIG : 2246
QGV56805.1 : IFRGDNALNMGRPKFLSDQIFNKVLFGSLYPTQFDYDEAGPSLAAGIQRGRERWGHPMSIYINQALHEIVRTIR-LAETVRGLRNVIDRNQIIGELNAFRTQLEDTRREVNNLIQTPEIQNNPTPEIIAAIQNWVQQYRGQITNLIDLIG : 2246
QPB67618.1 : IFRGDNALNMGRPKFLSDQIFNKVLFGSLYPTQFDYDEAGPSLAAGIQRGRERWGHPMSIYINQALHEIVRTIR-LAETVRGLRNVIDRNQIIGELNAFRTQLEDTRREVNNLIQTPEIQNNPTPEIIAAIQNWVQQYRGQITNLIDLIG : 2246
QUQ60377.1 : IFRGDNALNMGRPKFLSDQIFNKVLFGSLYPTQFDYDEAGPSLAAGIQRGRERWGHPMSIYINQALHEIVRTIR-LAETVRGLRNVIDRNQIIGELNAFRTQLEDTRREVNNLIQTPEIQNNPTPEIIAAIQNWVQQYRGQITNLIDLIG : 2246
QUQ60197.1 : IFRGDNALNMGRPKFLSDQIFNKVLFGSLYPTQFDYDEAGPSLAAGIQRGRERWGHPMSIYINQALHEIVRTIR-LAETVRGLRNVIDRNQIIGELNAFRTQLEDTRREVNNLIQTPEIQNNPTPEIIAAIQNWVQQYRGQITNLIDLIG : 2246
SPS73515.1 : IFRGDNALNMGRPKFLSDQIFNKVLFGSLYPTQFDYDEAGPSLAAGIQRGRERWGHPMSIYINQALHEIVRTIR-LAETVRGLRNVIDRNQIIGELNAFRTQLEDTRREVNNLIQTPEIQNNPTPEIIAAIQNWVQQYRGQITNLIDLIG : 2246
QDL88123.1 : IFRGDNALNMGRPKFLSDQIFNKVLFGSLYPTQFDYDEAGPSLAAGIQRGRERWGHPMSIYINQALHEIVRTIR-LAETVRGLRNVIDRNQIIGELNAFRTQLEDTRREVNNLIQTPEIQNNPTPEIIAAIQNWVQQYRGQITNLIDLIG : 2246
QIA61506.1 : IFRGDNALNMGRPKFLSDQIFNKVLFGSLYPTQFDYDEAGPSLAAGIQRGRERWGHPMSIYINQALHEIVRTIR-LAETVRGLRNVIDRNQIIGELNAFRTQLEDTRREVNNLIQTPEIQNNPTPEIIAAIQNWVQQYRGQITNLIDLIG : 2246
QTZ19752.1 : IFRGDNALNMGRPKFLSDQIFNKVLFGSLYPTQFDYDEAGPSLAAGIQRGRERWGHPMSIYINQALHEIVRTIR-LAETVRGLRNVIDRNQIIGELNAFRTQLEDTRREVNNLIQTPEIQNNPTPEIIAAIQNWVQQYRGQITNLIDLIG : 2246
QIM07715.1 : IFRGDNALNMGRPKFLSDQIFNKVLFGSLYPTQFDYDEAGPGLAAGIQRGREQWGQPLSEYINQALHELVRTIR-IPQKLRVLRNIIVKNQLIADLTTIREQLVSMRREVENMIQTPEIQNNPTPEVIAAAQNWTQQYRARVDTLINFIG : 2246
QIM07948.1 : IFRGDNALNMGRPKFLSDQIFNKVLFGSLYPTQFDYDEAGPGLAAGIQRGREQWGQPLSEYINQALHELVRTIR-IPQKLRVLRNIIVKNQLIADLTTIREQLVSMRREVENMIQTPEIQNNPTPEVIAAAQNWTQQYRARVDTLINFIG : 2246
QIM08183.1 : IFRGDNALNMGRPKFLSDQIFNKVLFGSLYPTQFDYDEAGPGLAAGIQRGREQWGQPLSEYINQALHELVRTIR-IPQKLRVLRNIIVKNQLIADLTTIREQLVSMRREVENMIQTPEIQNNPTPEVIAAAQNWTQQYRARVDTLINFIG : 2246
YP_0097024 : IFRGDNALNMGRPKFLSDQIFNKVLFGSLYPTQFDYDEAGPGLAAGIQRGREQWGQPLSEYINQALHELVRTIR-IPQKLRVLRNIIVKNQLIADLTTIREQLVSMRREVENMIQTPEIQNNPTPEVIAAAQNWTQQYRARVDTLINFIG : 2246
YP_0097038 : IFRGDNALNMGRPKFLSDQIFNKVLFGSLYPTQFDYDEAGPGLAAGIQRGREQWGQPLSEYINQALHELVRTIR-IPQKLRVLRNIIVKNQLIADLTTIREQLVSMRREVENMIQTPEIQNNPTPEVIAAAQNWTQQYRARVDTLINFIG : 2246
YP_0097023 : IFRGDNALNMGRPKFLSDQIFNKVLFGSLYPTQFDYDEAGPGLAAGIQRGREQWGQPLSEYINQALHELVRTIR-IPQKLRVLRNIIVKNQLIADLTTIREQLVSMRREVENMIQTPEIQNNPTPEVIAAAQNWTQQYRARVDTLINFIG : 2246
YP_0097026 : IFRGDNALNMGRPKFLSDQIFNKVLFGSLYPTQFDYDEAGPGLAAGIQRGREQWGQPLSEYINQALHELVRTIR-IPQKLRVLRNIIVKNQLIADLTTIREQLVSMRREVENMIQTPEIQNNPTPEVIAAAQNWTQQYRARVDTLINFIG : 2246
YP_0097035 : IFRGDNALNMGRPKFLSDQIFNKVLFGSLYPTQFDYDEAGPGLAAGIQRGREQWGQPLSEYINQALHELVRTIR-IPQKLRVLRNIIVKNQLIADLTTIREQLVSMRREVENMIQTPEIQNNPTPEVIAAAQNWTQQYRARVDTLINFIG : 2246
QZK26792.1 : IFRGDNALNMGRPKFLSDQIFNKVLFGSLYPTQFDYDEAGPGLAAGIQRGRERWGHPMSIYINQALHEIVRTIR-LAETVRGLRNVIDRNQIVGELNAFRTQLENTRREVNNLVQTPEIQNNPTPEIIAAVQNWGQQYRGQITNLIDLIG : 2246
YP_0097036 : IFRGDNALNMGRPKFLSDQIFNKVLFGSLYPTQFDYDEAGPGLAAGIQRGREQWGQPLSEYINQALHELVRTIR-IPQKLRVLRNIIVKNQLIADLTTIREQLVSMRREVENMIQTPEIQNNPTPEVIAAAQNWTQQYRARVDTLINFIG : 2246
QID21266.1 : IFRGDNALNMGRPKFLSDQIFNKVLFGSLYPTQFDYDEAGPGLAAGIQRGRERWGHPMSIYINQALHEIVRTIR-LAETVRGLRNVIDRNQIVGELNAFRTQLENTRREVNNLVQTPEIQNNPTPEIIAAVQNWGQQYRGQITNLIDLIG : 2245
P0CA04.1   : IFRGDNALNMGRPKFLSDQIFNKVLFGSLYPTQFDYDEAGPGLAAGIQRGRERWGHPMSIYINQALHEIVRTIR-LAETVRGLRNVIDRNQIIGELNAFRTQLEDTRREVNNLIQTPEIQNNPTPEIIAAIQNWVQQYRGQITNLIDLIG : 2245
P0CA02.1   : IFRGDNALNMGRPKFLSDQIFNKVLFGSLYPTQFDYDEAGPSLAAGIQRGRERWGHPMSIYINQALHEIVRTIR-LAETVRGLRNVIDRNQIIGELNAFRTQLEDTLREVNNLVQTPEIQNNPTPEIIAAVQNWGQQYRAQITDLIDLIG : 2245
YP_0097029 : IFRGDNALNMGRPKFLSDQIFNKVLFGSLYPTQFDYDEAGPGLAAGIQRGRERWGHPMSIYINQALHEVVRTIR-LAETVRGLRNVIDRNQIIDELNAFRTQLEDTRREVDNLLQTPEIQNNVIPEIIAAVQNWGQQYRGQITDLIDLIG : 2246
AXB50010.1 : IFRGDNALNMGRPKFLSDQIFNKVLFGSLYPTQFDYDEAGPGLAAGIQRGRERWGHPMSIYINQALHEVVRTIR-LAETVRGLRNVIDRNQIIDELNAFRTQLEDTRREVDNLLQTPEIQNNVIPEIIAAVQNWGQQYRGQITDLIDLIG : 2247
AXB49666.1 : IFRGDNALNMGRPKFLSDQIFNKVLFGSLYPTQFDYDEAGPGLAAGIQRGRERWGHPMSIYINQALHEVVRTIR-LAETVRGLRNVIDRNQIIDELNAFRTQLEDTRREVDNLLQTPEIQNNVIPEIIAAVQNWGQQYRGQITDLIDLIG : 2247
CAD7112308 : IFRGDNALNMGRPKFLSDQIFNKVLFGSLYPTQFDYDEAGPGLAAGIQRGREQWGQPFSDYINQALHELVRTIR-IPQNLRVLRNIMVKNQLIADLAAIREQLVRMRREVENMVQTPEIQNNPTPEVIAAAQTWAQQYRARVDFLINFIG : 2245
QRY19118.1 : IFRGDNALNMGRPKFLSDQIFNKVLFGSLYPTQFDYDEAGPGLAAGIQRGREQWGQPFSDYINQALHELVRTIR-IPQNLRVLRNIMVKNQLIADLAAIREQLVRMRREVENMVQTPEIQNNPTPEVIAAAQTWAQQYRARVDFLINFIG : 2245
P0CA01.1   : IFRGDNALNMGRPKFLSDQIFNKVLFGSLYPTQFDYDEAGPGLAAGIQRGREQWGQPLSDYINQALHELVRTIRIIPQNIRVLRNIMVKNQLIADLAAIREQLVRMRREVENMVQTPEIQNNPTPEVIAAAQTWTQQYRARVDFLINFIG : 2246
YP_0097028 : IFRGDNALNMGRPKFLSDQIFNKVLFGSLYPTQFDYDEAGPGLAAGIQRGREQWGQPFSDYINQALHELVRTIR-IPQNLRVLRNIMVKNQLIADLAAIREQLVRMRREVENMVQTPEIQNNPTPEVIAAAQTWAQQYRARVDFLINFIG : 2245
P0CA03.1   : IFRGDNALNMGRPKFLSDQIFNKVLFGSLYPTQFDYDEAGPSLAAGIQRGRERWGHPMSIYINQALHEIVRTIR-LAETVRGLRNVIDRNQIIGELNAFRTQLEDTRREVNNLIQTPEIRNNPTPEIIAAVQNWGQQYRGQITDLIDLIG : 2245
```

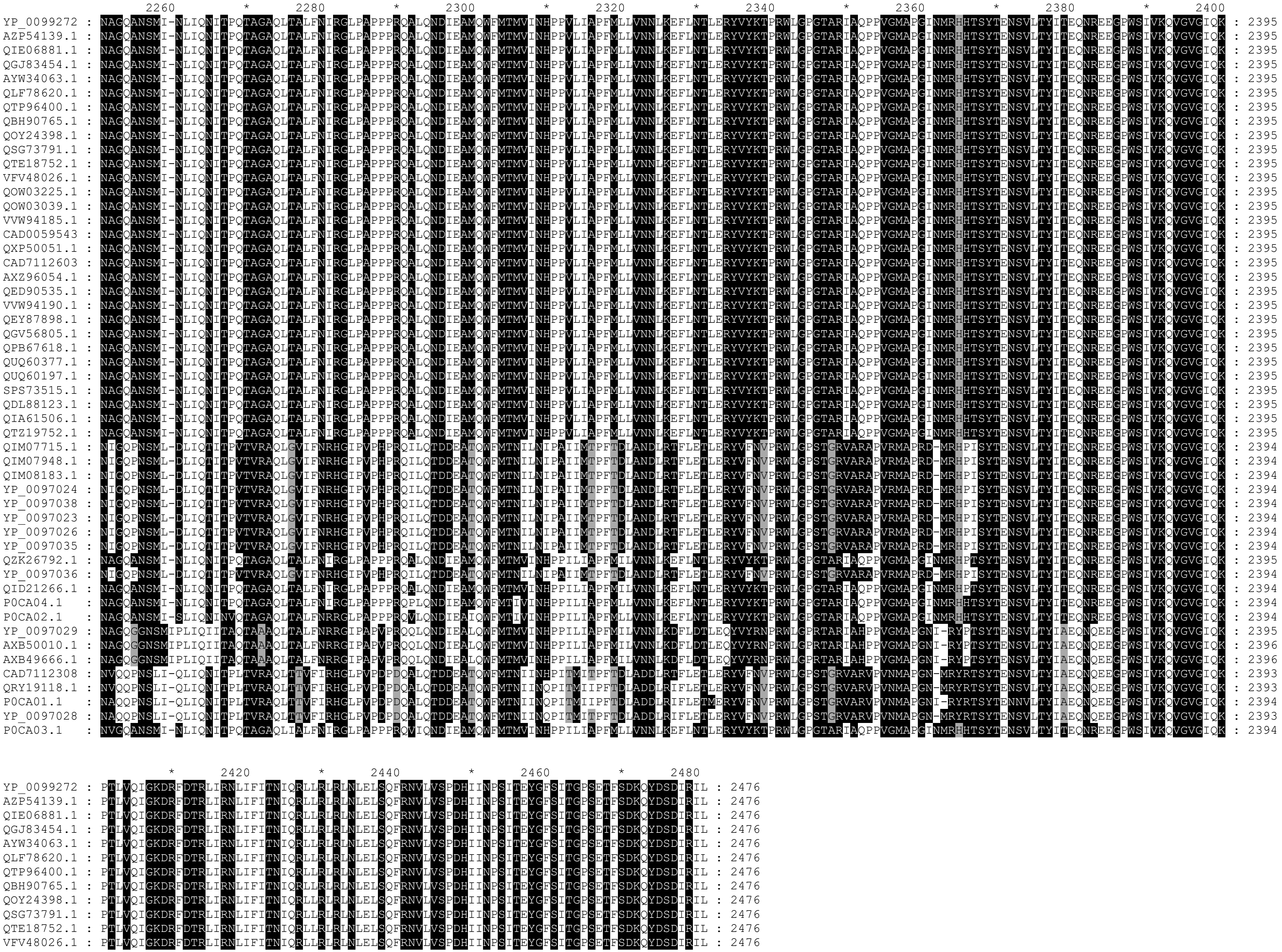

```
QOW03225.1 : PTLVQIGKDRFDTRLIRNLIFITNIQRLLRLRLNLELSQFRNVLVSPDHIINPSITEYGFSITGPSETFSDKQYDSDIRIL : 2476
QOW03039.1 : PTLVQIGKDRFDTRLIRNLIFITNIQRLLRLRLNLELSQFRNVLVSPDHIINPSITEYGFSITGPSETFSDKQYDSDIRIL : 2476
VVW94185.1 : PTLVQIGKDRFDTRLIRNLIFITNIQRLLRLRLNLELSQFRNVLVSPDHIINPSITEYGFSITGPSETFSDKQYDSDIRIL : 2476
CAD0059543 : PTLVQIGKDRFDTRLIRNLIFITNIQRLLRLRLNLELSQFRNVLVSPDHIINPSITEYGFSITGPSETFSDKQYDSDIRIL : 2476
QXP50051.1 : PTLVQIGKDRFDTRLIRNLIFITNIQRLLRLRLNLELSQFRNVLVSPDHIINPSITEYGFSITGPSETFSDKQYDSDIRIL : 2476
CAD7112603 : PTLVQIGKDRFDTRLIRNLIFITNIQRLLRLRLNLELSQFRNVLVSPDHIINPSITEYGFSITGPSETFSDKQYDSDIRIL : 2476
AXZ96054.1 : PTLVQIGKDRFDTRLIRNLIFITNIQRLLRLRLNLELSQFRNVLVSPDHIINPSITEYGFSITGPSETFSDKQYDSDIRIL : 2476
QED90535.1 : PTLVQIGKDRFDTRLIRNLIFITNIQRLLRLRLNLELSQFRNVLVSPDHIINPSITEYGFSITGPSETFSDKQYDSDIRIL : 2476
VVW94190.1 : PTLVQIGKDRFDTRLIRNLIFITNIQRLLRLRLNLELSQFRNVLVSPDHIINPSITEYGFSITGPSETFSDKQYDSDIRIL : 2476
QEY87898.1 : PTLVQIGKDRFDTRLIRNLIFITNIQRLLRLRLNLELSQFRNVLVSPDHIINPSITEYGFSITGPSETFSDKQYDSDIRIL : 2476
QGV56805.1 : PTLVQIGKDRFDTRLIRNLIFITNIQRLLRLRLNLELSQFRNVLVSPDHIINPSITEYGFSITGPSETFSDKQYDSDIRIL : 2476
QPB67618.1 : PTLVQIGKDRFDTRLIRNLIFITNIQRLLRLRLNLELSQFRNVLVSPDHIINPSITEYGFSITGPSETFSDKQYDSDIRIL : 2476
QUQ60377.1 : PTLVQIGKDRFDTRLIRNLIFITNIQRLLRLRLNLELSQFRNVLVSPDHIINPSITEYGFSITGPSETFSDKQYDSDIRIL : 2476
QUQ60197.1 : PTLVQIGKDRFDTRLIRNLIFITNIQRLLRLRLNLELSQFRNVLVSPDHIINPSITEYGFSITGPSETFSDKQYDSDIRIL : 2476
SPS73515.1 : PTLVQIGKDRFDTRLIRNLIFITNIQRLLRLRLNLELSQFRNVLVSPDHIINPSITEYGFSITGPSETFSDKQYDSDIRIL : 2476
QDL88123.1 : PTLVQIGKDRFDTRLIRNLIFITNIQRLLRLRLNLELSQFRNVLVSPDHIINPSITEYGFSITGPSETFSDKQYDSDIRIL : 2476
QIA61506.1 : PTLVQIGKDRFDTRLIRNLIFITNIQRLLRLRLNLELSQFRNVLVSPDHIINPSITEYGFSITGPSETFSDKQYDSDIRIL : 2476
QTZ19752.1 : PTLVQIGKDRFDTRLIRNLIFITNIQRLLRLRLNLELSQFRNVLVSPDHIINPSITEYGFSITGPSETFSDKQYDSDIRIL : 2476
QIM07715.1 : PTLVQIGKDRFDTRLIRNLIFITNIQRLLRLRLNLELSQFRNVLVSPDHIINPSITEYGFSITGPSETFSDKQYDSDIRIL : 2475
QIM07948.1 : PTLVQIGKDRFDTRLIRNLIFITNIQRLLRLRLNLELSQFRNVLVSPDHIINPSITEYGFSITGPSETFSDKQYDSDIRIL : 2475
QIM08183.1 : PTLVQIGKDRFDTRLIRNLIFITNIQRLLRLRLNLELSQFRNVLVSPDHIINPSITEYGFSITGPSETFSDKQYDSDIRIL : 2475
YP_0097024 : PTLVQIGKDRFDTRLIRNLIFITNIQRLLRLRLNLELSQFRNVLVSPDHIINPSITEYGFSITGPSETFSDKQYDSDIRIL : 2475
YP_0097038 : PTLVQIGKDRFDTRLIRNLIFITNIQRLLRLRLNLELSQFRNVLVSPDHIINPSITEYGFSITGPSETFSDKQYDSDIRIL : 2475
YP_0097023 : PTLVQIGKDRFDTRLIRNLIFITNIQRLLRLRLNLELSQFRNVLVSPDHIINPSITEYGFSITGPSETFSDKQYDSDIRIL : 2475
YP_0097026 : PTLVQIGKDRFDTRLIRNLIFITNIQRLLRLRLNLELSQFRNVLVSPDHIINPSITEYGFSITGPSETFSDKQYDSDIRIL : 2475
YP_0097035 : PTLVQIGKDRFDTRLIRNLIFITNIQRLLRLRLNLELSQFRNVLVSPDHIINPSITEYGFSITGPSETFSDKQYDSDIRIL : 2475
QZK26792.1 : PTLVQIGKDRFDTRLIRNLIFITNIQRLLRLRLNLELSQFRNVLVSPDHIINPSITEYGFSITGPSETFSDKQYDSDIRIL : 2476
YP_0097036 : PTLVQIGKDRFDTRLIRNLIFITNIQRLLRLRLNLELSQFRNVLVSPDHIINPSITEYGFSITGPSETFSDKQYDSDIRIL : 2475
QID21266.1 : PTLVQIGKDRFDTRLIRNLIFITNIQRLLRLRLNLELSQFRNVLVSPDHIINPSITEYGFSITGPSETFSDKQYDSDIRIL : 2475
P0CA04.1   : PTLVHIGKDRFDTRLIRNLIFITNIQRLLRLRLNLELSQFRNVLVSPDHIINPSITEYGFSITGPSETFSDKQYDSDIRIL : 2475
P0CA02.1   : PTLVQIGKDRFDTRLIRNLIFITNVQRLLRLRLNLELSQFRNVLVSPDHIINPSITEYGFSITGPSETFSDKQYDSDIRIL : 2475
YP_0097029 : PALIQIGKDRFDTRLIRNLIFITNIQRLLRLRLNLELSQFRNVLVSPDHIINPSITEYGFSITGPSETFSDKQYDSDIRIL : 2476
AXB50010.1 : PALIQIGKDRFDTRLIRNLIFITNIQRLLRLRLNLELSQFRNVLVSPDHIINPSITEYGFSITGPSETFSDKQYDSDIRIL : 2477
AXB49666.1 : PALIQIGKDRFDTRLIRNLIFITNIQRLLRLRLNLELSQFRNVLVSPDHIINPSITEYGFSITGPSETFSDKQYDSDIRIL : 2477
CAD7112308 : PALIQIGKDRFDTRLIRNLIFITNIQRLLRLRLNLELSQFRNVLVSPDHIINPSITEYGFSITGPSETFSDKQYDSDIRIL : 2474
QRY19118.1 : PALIQIGKDRFDTRLIRNLIFITNIQRLLRLRLNLELSQFRNVLVSPDHIINPSITEYGFSITGPSETFSDKQYDSDIRIL : 2474
P0CA01.1   : PALIQIGKDRFDTRLIRNLIFITNIQRLLRLRLNLELSQFRNVLVSPNHIINPSITEYGFSITGPSETFSDKQYDSDIRIL : 2475
YP_0097028 : PALIQIGKDRFDTRLIRNLIFITNIQRLLRLRLNLELSQFRNVLVSPDHIINPSITEYGFSITGPSETFSDKQYDSDIRIL : 2474
P0CA03.1   : PTLVQIGKDRFDTRLIRNLIFITNIQRLLRLRLNLELSQFRNVLVSPDHIINPSITEYGFSITGPSETFSDKQYDSDIRIL : 2475
                              i
```

Supplementary Table 1: List of ASFV isolates used as the source of the pp220 sequences (https://www.ncbi.nlm.nih.gov/) and aligned in the supplementary Figure 1.

Accession #	Isolate; Country	Genotype; Source of isolation	References
NC_044959.2 YP_009927216/ CAD2068454	ASFV Georgia 2007/1 Georgia	Genotype II Domestic Pig	(Chapman et al., 2011)
MH910496.1 AZP54139.1	Georgia 2008/2 Georgia	Genotype II; Domestic Pig	(Farlow et al., 2018)
MN393476.1 QIE06881.1	ASFV Wuhan 2019-1 China	Genotype II Domestic pig	(Xiong, Zhang, Yu, & Wei, 2019)
MN172368.1 QGJ83454	China/CAS19-01/2019 China	Genotype II Domestic pig	(Jia et al., 2020)
MK128995.1 AYW34063.1	China/2018/AnhuiXCGQ China	Genotype II Domestic pig	(Bao et al., 2019)
MT496893.1 QLF78620.1	GZ201801 China	Genotype II Domestic pig - serum	(Tran et al., 2022)
MW521382.1 QTP96400.1	HuB20 China	Genotype II Domestic pig	(Tran et al., 2022)
MK333181.1 QBH90765.1	DB/LN/2018 China	Genotype II Domestic Pig - Blood	(Wen et al., 2019)
MT180393.1 QOY24398.1	ASFV_NgheAn_2019 Vietnam	Genotype II Domestic pig	(Nguyen et al., 2021)
MW465755.1 QSG73791.1	VNUA-ASFV-05L1/HaNam/VN/2020 Vietnam	Genotype II Domestic pig -spleen	(Truong et al., 2021)
MW396979.1 QTE18752.1	ASFV/Timor-Leste/2019/1 Timor-Leste	Genotype II Domestic pig	(Mileto et al., 2021)
LR536725.1 VFV48026.1	ASFV Belgium 2018/1 Belgium	Genotype II Domestic Pig	(Jan H. Forth et al., 2019)
MT847623.2 QOW03225.1	Pol19_53050_C1959/19 Poland	Genotype II Domestic pig	(Mazur-Panasiuk, 2020)
MT847622.1 QOW03039.1	Pol17_31177_O81 Poland	Genotype II Domestic pig	(Mazur-Panasiuk, 2020)
LR722599.1 VVW94185.1	ASFV Moldova 2017/1 Moldova	Genotype II Domestic pig - spleen	(J. H. Forth et al., 2019)
LR813622.1 CAD0059543.1	Tanzania/Rukwa/2017/1 SW Tanzania	Genotype II Domestic pig	(Njau et al., 2021)
MW856068.1 QXP50051.1	MAL/19/Karonga Malawi	Genotype II Domestic pig	(Hakizimana et al., 2020)
LR899193.1 CAD7112603.1	ASFV Germany 2020/1 Germany	Genotype II Wild Boar	(Sauter-Louis, 2021)

MG939587.1<br>AXZ96054.1	Pol17_03029_C201<br>Poland	Genotype II<br>Wild boar	(Mazur-Panasiuk, Woźniakowski, & Niemczuk, 2019)
MK543947.1<br>QED90535	Belgium/Etalle/wb/2018<br>Belgium	Genotype II<br>Wild Boar	(Garigliany et al., 2019; Gilliaux et al., 2019)
LR722600.1<br>VVW94190.1	ASFV CzechRepublic 2017/1<br>Czech Republic	Genotype II<br>Wild Boar	(Forth, 2020)
MK628478.1<br>QEY87898.1	ASFV/LT14/1490<br>Lithuania	Genotype II<br>Wild Boar - Blood	(Gallardo et al., 2014)
MN715134.1<br>QGV56805.1	ASFV_HU_2018<br>Hungary	Genotype II<br>Wild Boar - alveolar macrophages	(Olasz et al., 2019)
MT459800.1<br>QPB67618.1	ASFV/Kabardino-Balkaria 19/WB-964<br>Russia	Genotype II<br>Wild Boar - spleen	(Malogolovkin, Yelsukova, Gallardo, Tsybanov, & Kolbasov, 2012; Mazloum et al., 2021)
MW306191.1<br>QUQ60377.1	ASFV/Primorsky 19/WB-6723<br>Russia	Genotype II<br>Wild Boar - spleen	(Mazloum et al., 2021)
MW306190.1<br>QUQ60197.1	ASFV/Amur 19/WB-6905<br>Russia	Genotype II<br>Wild Boar - spleen	(Mazloum et al., 2021)
LS478113.1<br>SPS73515.1	Estonia 2014<br>Estonia	Genotype II<br>Wild Boar	(Nurmoja et al., 2020; Zani et al., 2018)
MK645909.1<br>QDL88123.1	ASFV-wbBS01<br>China	Genotype II<br>Wild Boar	(Bao et al., 2019);(Njau et al., 2021)
MK940252.1<br>QIA61506.1	CN/2019/InnerMongolia-AES01-<br>China	Genotype II<br>Wild Boar	(Tran et al., 2022)
MW701371.1<br>QTZ19752.1	ASFV-G-deltaI177LdeltaLVR<br>Plum Island	synthetic construct – deletion mutant of Georgia2007//1	(Borca, 2021)
MN270973.1<br>QIM07715.1	85/Ca/1985<br>Italy: Cagliari, Sardinia	Genotype I<br>Domestic pig	(Torresi et al., 2020)
MN270974.1<br>QIM07948.1	141/Nu/1990<br>Italy: Nuoro, Sardinia	Genotype I<br>Domestic pig	(Nix, Gallardo, Hutchings, Blanco, & Dixon, 2006; Torresi et al., 2020)
MN270975.1<br>QIM08183.1	142/Nu/1995<br>Italy: Nuoro, Sardinia	Genotype I<br>Domestic pig	(Torresi et al., 2020)
NC_044942.1<br>YP_009702497.1	BA71V<br>Spain: Badajoz	Genotype I<br>Domestic pig - spleen	(Yanez, 1995)
NC_044958.1<br>YP_009703860.1	E75<br>Spain	Genotype I<br>Domestic pig - spleen	(de Villiers et al., 2010)
NC_044941.1<br>YP_009702339.1	L60<br>Portugal	Genotype I<br>Domestic pig	(Bastos et al., 2003)
NC_044943.1<br>YP_009702658.1	NHV<br>Portugal	Genotype I<br>Domestic pig	(Portugal et al., 2015)
NC_044956.1<br>YP_009703544.1/<br>CAN10191	Benin 97/1<br>Benin	Genotype I<br>Domestic Pig	(Bastos et al., 2003)

MZ202520.1<br>QZK26792.1	K49<br>Zaire - Katanga	Genotype I<br>Serogroup 2	(Bastos et al., 2003)
NC_044957.1<br>YP_009703699.1	OURT 88/3<br>Portugal	Genotype I<br>Tick	(Boinas, Hutchings, Dixon, & Wilkinson, 2004)
MN913970.1<br>QID21266.1	Liv13/33 (OmLF2)<br>Zambia, Livingstone	Genotype I<br>Tick	(Chastagner et al., 2020)
gi|229891462<br>AY261366.1<br>P0CA04.1	Namibia/Wart80/1980<br>Namibia	Genotype IV<br>Warthog	(Bastos et al., 2003; Zsak et al., 2005)
AY261361.1<br>P0CA02.1	Malawi LIL 20/1<br>Malawi	Genotype VIII; Haplotype 8a<br>Ticks	(Haresnape, 1989)
NC_044946.1<br>YP_009702990.1	Ken06.Bus<br>Kenya	Genotype IX<br>Domestic Pig	(Bishop et al., 2015; Gallardo et al., 2009)
MH025920.1<br>AXB50010.1	R35<br>Uganda: Tororo district	Genotype IX<br>Domestic pig<br>Blood	(Masembe et al., 2018)
MH025918.1<br>AXB49666.1	R25<br>Uganda: Tororo district	Genotype IX<br>Domestic pig<br>Blood	(Masembe et al., 2018)
LR899131.1<br>CAD7112308.1	ASFV Ken.rie1	Genotype X<br>Domestic Pig - Blood	(Forth et al., 2020)
MT956648.1<br>QRY19118.1	Uvira B53<br>Kivu – DR Congo	Genotype X<br>Serogroup 7<br>Domestic pig - spleen	(Bisimwa et al., 2020)
AY261360.1<br>P0CA01.1gi|229891459	KEN-50/1950<br>Kenya	Genotype X<br>Domestic Pig	Kutish G.F. and Rock D.L, 2003
NC_044945.1<br>YP_009702825.1<br>HM745253	Ken05/Tk1<br>Kenya	Genotype X<br>Ticks	(Bishop et al., 2015; Gallardo et al., 2009)
gi|229891461<br>AY261363.1<br>P0CA03.1	Pretoriuskop Pr4/1996<br>South Africa	Genotype XX<br>Haplotype 20a<br>Ticks	(Kleiboeker, Burrage, Scoles, Fish, & Rock, 1998)

Supplementary Table 2. Defined IFN-γ-inducing pp220 peptides are predicted to bind to multiple *SLA-I* alleles

Peptide	Sequence	Aff(nM)	Bind Level	*SLA-I* Allele
$p34^{161\text{-}169}$	LTHGLRAEY	1486.83	SB	SLA-1*02:01
$p34^{161\text{-}169}$	LTHGLRAEY	1486.83	SB	SLA-1*02:02
$p34^{161\text{-}169}$	LTHGLRAEY	270.87	SB	SLA-1*07:01
$p34^{161\text{-}169}$	LTHGLRAEY	270.87	SB	SLA-1*07:02
$p34^{161\text{-}169}$	LTHGLRAEY	1495.02	SB	SLA-1*08:01
$p34^{161\text{-}169}$	LTHGLRAEY	538.61	SB	SLA-1*LWH
$p34^{161\text{-}169}$	LTHGLRAEY	2054.49	SB	SLA-2*01:01
$p34^{161\text{-}169}$	LTHGLRAEY	1397.84	SB	SLA-2*01:02
$p34^{161\text{-}169}$	LTHGLRAEY	2046.26	SB	SLA-2*03:02
$p34^{161\text{-}169}$	LTHGLRAEY	306.17	SB	SLA-2*04:01
$p34^{161\text{-}169}$	LTHGLRAEY	1828.05	SB	SLA-2*04:02
$p34^{161\text{-}169}$	LTHGLRAEY	1903.42	SB	SLA-2*10:01
$p34^{161\text{-}169}$	LTHGLRAEY	1005.50	SB	SLA-2*10:02
$p34^{161\text{-}169}$	LTHGLRAEY	451.91	SB	SLA-2*HB:01
$p34^{161\text{-}169}$	LTHGLRAEY	4407.40	SB	SLA-2*LWH:AA
$p34^{161\text{-}169}$	LTHGLRAEY	3744.41	SB	SLA-2*TPK:AA
$p37^{859\text{-}867}$	KSMAAKIFI	1177.68	SB	SLA-2*05:01
$p37^{859\text{-}867}$	KSMAAKIFI	7913.59	SB	SLA-2*12:01
$p37^{859\text{-}867}$	KSMAAKIFI	1586.28	SB	SLA-2*YC:AA
$p37^{859\text{-}867}$	KSMAAKIFI	1201.01	SB	SLA-3*01:01
$p37^{859\text{-}867}$	KSMAAKIFI	2335.79	SB	SLA-3*06:02
$p150^{1363\text{-}1371}$	HIDKNIIQY	9776.79	SB	SLA-1*01:01
$p150^{1363\text{-}1371}$	HIDKNIIQY	520.01	SB	SLA-1*02:01
$p150^{1363\text{-}1371}$	HIDKNIIQY	520.01	SB	SLA-1*02:02
$p150^{1363\text{-}1371}$	HIDKNIIQY	13.25	SB	SLA-1*04:01
$p150^{1363\text{-}1371}$	HIDKNIIQY	926.75	SB	SLA-1*06:01
$p150^{1363\text{-}1371}$	HIDKNIIQY	420.20	SB	SLA-1*07:01
$p150^{1363\text{-}1371}$	HIDKNIIQY	420.20	SB	SLA-1*07:02
$p150^{1363\text{-}1371}$	HIDKNIIQY	554.29	SB	SLA-1*08:01
$p150^{1363\text{-}1371}$	HIDKNIIQY	3350.95	SB	SLA-1*12:01
$p150^{1363\text{-}1371}$	HIDKNIIQY	303.44	SB	SLA-1*13:01
$p150^{1363\text{-}1371}$	HIDKNIIQY	2759.77	SB	SLA-1*HB:01
$p150^{1363\text{-}1371}$	HIDKNIIQY	2038.59	SB	SLA-1*HB:02
$p150^{1363\text{-}1371}$	HIDKNIIQY	2038.59	SB	SLA-1*HB:03
$p150^{1363\text{-}1371}$	HIDKNIIQY	2759.77	SB	SLA-1*HB:04
$p150^{1363\text{-}1371}$	HIDKNIIQY	1181.03	SB	SLA-1*LWH

Peptide	Sequence	Aff(nM)	Bind Level	*SLA-I* Allele
p150$^{1363\text{-}1371}$	HIDKNIIQY	3227.75	SB	SLA-1*YC
p150$^{1363\text{-}1371}$	HIDKNIIQY	13.25	SB	SLA-1*YDL:01
p150$^{1363\text{-}1371}$	HIDKNIIQY	7612.26	SB	SLA-2*01:01
p150$^{1363\text{-}1371}$	HIDKNIIQY	3376.90	SB	SLA-2*01*02
p150$^{1363\text{-}1371}$	HIDKNIIQY	1348.91	SB	SLA-2*03:02
p150$^{1363\text{-}1371}$	HIDKNIIQY	6358.65	SB	SLA-2*04:01
p150$^{1363\text{-}1371}$	HIDKNIIQY	5207.98	SB	SLA-2*10:01
p150$^{1363\text{-}1371}$	HIDKNIIQY	220.50	SB	SLA-2*10:02
p150$^{1363\text{-}1371}$	HIDKNIIQY	213.00	SB	SLA-2*HB:01
p150$^{1363\text{-}1371}$	HIDKNIIQY	8411.18	SB	SLA-2*LWH:AA
p150$^{1363\text{-}1371}$	HIDKNIIQY	14014.36	SB	SLA-2*TPK:AA
p150$^{1363\text{-}1371}$	HIDKNIIQY	13.25	SB	SLA-2*YDL:AA
p150$^{1363\text{-}1371}$	HIDKNIIQY	13.25	SB	SLA-2*YDL:02
p150$^{1363\text{-}1371}$	HIDKNIIQY	20574.55	SB	SLA-3*03:02
p150$^{1363\text{-}1371}$	HIDKNIIQY	2759.77	SB	SLA-3*LWH
p150$^{1463\text{-}1471}$	RVFSRLVFY	168.64	SB	SLA-1*02:01
p150$^{1463\text{-}1471}$	RVFSRLVFY	168.64	SB	SLA-1*02:02
p150$^{1463\text{-}1471}$	RVFSRLVFY	157.58	SB	SLA-1*04:01
p150$^{1463\text{-}1471}$	RVFSRLVFY	1041.78	SB	SLA-1*05:01
p150$^{1463\text{-}1471}$	RVFSRLVFY	1255.39	SB	SLA-1*06:01
p150$^{1463\text{-}1471}$	RVFSRLVFY	84.92	SB	SLA-1*07:01
p150$^{1463\text{-}1471}$	RVFSRLVFY	84.92	SB	SLA-1*07:02
p150$^{1463\text{-}1471}$	RVFSRLVFY	402.78	SB	SLA-1*08:01
p150$^{1463\text{-}1471}$	RVFSRLVFY	319.31	SB	SLA-1*12:01
p150$^{1463\text{-}1471}$	RVFSRLVFY	1212.39	SB	SLA-1*13:01
p150$^{1463\text{-}1471}$	RVFSRLVFY	1181.63	SB	SLA-1*HB:01
p150$^{1463\text{-}1471}$	RVFSRLVFY	808.38	SB	SLA-1*HB:02
p150$^{1463\text{-}1471}$	RVFSRLVFY	808.38	SB	SLA-1*HB:03
p150$^{1463\text{-}1471}$	RVFSRLVFY	1181.63	SB	SLA-1*HB:04
p150$^{1463\text{-}1471}$	RVFSRLVFY	248.15	SB	SLA-1*LWH
p150$^{1463\text{-}1471}$	RVFSRLVFY	813.92	SB	SLA-1*YC
p150$^{1463\text{-}1471}$	RVFSRLVFY	157.58	SB	SLA-1*YDL:01
p150$^{1463\text{-}1471}$	RVFSRLVFY	548.67	SB	SLA-2*01:01
p150$^{1463\text{-}1471}$	RVFSRLVFY	130.67	SB	SLA-2*01:02
p150$^{1463\text{-}1471}$	RVFSRLVFY	463.66	SB	SLA-2*03:02
p150$^{1463\text{-}1471}$	RVFSRLVFY	314.89	SB	SLA-2*04:01
p150$^{1463\text{-}1471}$	RVFSRLVFY	1264.22	SB	SLA-2*04:02
p150$^{1463\text{-}1471}$	RVFSRLVFY	730.71	SB	SLA-2*10:01
p150$^{1463\text{-}1471}$	RVFSRLVFY	108.09	SB	SLA-2*10:02
p150$^{1463\text{-}1471}$	RVFSRLVFY	61.50	SB	SLA-2*HB:01

Peptide	Sequence	Aff(nM)	Bind Level	*SLA-I* Allele
p150$^{1463\text{-}1471}$	RVFSRLVFY	1138.60	SB	SLA-2*LWH:AA
p150$^{1463\text{-}1471}$	RVFSRLVFY	5781.27	SB	SLA-2*TPK:AA
p150$^{1463\text{-}1471}$	RVFSRLVFY	157.58	SB	SLA-2*YDL:AA
p150$^{1463\text{-}1471}$	RVFSRLVFY	814.61	SB	SLA-2*YDY:AA
p150$^{1463\text{-}1471}$	RVFSRLVFY	157.58	SB	SLA-2*YDL:02
p150$^{1463\text{-}1471}$	RVFSRLVFY	2479.14	SB	SLA-3*03:01
p150$^{1463\text{-}1471}$	RVFSRLVFY	3692.27	SB	SLA-3*03:02
p150$^{1463\text{-}1471}$	RVFSRLVFY	2479.14	SB	SLA-3*03:03
p150$^{1463\text{-}1471}$	RVFSRLVFY	3223.21	SB	SLA-*:03:04
p150$^{1463\text{-}1471}$	RVFSRLVFY	1229.98	SB	SLA-3*04:01
p150$^{1463\text{-}1471}$	RVFSRLVFY	814.61	SB	SLA-3*06:01
p150$^{1463\text{-}1471}$	RVFSRLVFY	4138.02	SB	SLA-3*06:02
p150$^{1463\text{-}1471}$	RVFSRLVFY	1871.42	SB	SLA-3*07:01
p150$^{1463\text{-}1471}$	RVFSRLVFY	814.61	SB	SLA-3*CDY
p150$^{1463\text{-}1471}$	RVFSRLVFY	1181.63	SB	SLA-3*LWH
p150$^{1463\text{-}1471}$	RVFSRLVFY	1301.51	SB	SLA-3*YC
p150$^{1463\text{-}1471}$	RVFSRLVFY	814.61	SB	SLA-3*YDY:01
p150$^{1463\text{-}1471}$	RVFSRLVFY	814.61	SB	SLA-3*YDY:02
p150$^{1463\text{-}1471}$	RVFSRLVFY	1871.42	SB	SLA-3*YTH

The mapped IFN-γ-inducing nanomer peptides from ASFV pp220 (Georgia 2007/1) were predicted for *SLA-I* binding using NetMHCpan version 4.1 (https://services.healthtech.dtu.dk/service.php?NetMHCpan-4.1). Input type used was "Peptide" with the threshold for strong binders set at less than 0.5% Rank and for weak binders less than 2% Rank, but greater than 0.5% as are the default binding thresholds proven for MHC class I. Binding affinity and sorting were applied to filter for strong (SB) allele binders shown in the above table.
